# Defining the characteristics of interferon-alpha–stimulated human genes: insight from expression data and machine learning

**DOI:** 10.1093/gigascience/giac103

**Published:** 2022-11-18

**Authors:** Haiting Chai, Quan Gu, David L Robertson, Joseph Hughes

**Affiliations:** MRC-University of Glasgow Centre for Virus Research, Sir Michael Stoker Building, Garscube Campus, Campus, 464 Bearsden Road, Glasgow, G61 1QH, Scotland, UK; MRC-University of Glasgow Centre for Virus Research, Sir Michael Stoker Building, Garscube Campus, Campus, 464 Bearsden Road, Glasgow, G61 1QH, Scotland, UK; MRC-University of Glasgow Centre for Virus Research, Sir Michael Stoker Building, Garscube Campus, Campus, 464 Bearsden Road, Glasgow, G61 1QH, Scotland, UK; MRC-University of Glasgow Centre for Virus Research, Sir Michael Stoker Building, Garscube Campus, Campus, 464 Bearsden Road, Glasgow, G61 1QH, Scotland, UK

**Keywords:** antiviral response, interferon, interferon-stimulated genes, omics data analyses, machine learning

## Abstract

**Background:**

A virus-infected cell triggers a signalling cascade, resulting in the secretion of interferons (IFNs), which in turn induces the upregulation of the IFN-stimulated genes (ISGs) that play a role in antipathogen host defence. Here, we conducted analyses on large-scale data relating to evolutionary gene expression, sequence composition, and network properties to elucidate factors associated with the stimulation of human genes in response to IFN-α.

**Results:**

We find that ISGs are less evolutionary conserved than genes that are not significantly stimulated in IFN experiments (non-ISGs). ISGs show obvious depletion of GC content in the coding region. This influences the representation of some compositions following the translation process. IFN-repressed human genes (IRGs), downregulated genes in IFN experiments, can have similar properties to the ISGs. Additionally, we design a machine learning framework integrating the support vector machine and novel feature selection algorithm that achieves an area under the receiver operating characteristic curve (AUC) of 0.7455 for ISG prediction. Its application in other IFN systems suggests the similarity between the ISGs triggered by type I and III IFNs.

**Conclusions:**

ISGs have some unique properties that make them different from the non-ISGs. The representation of some properties has a strong correlation with gene expression following IFN-α stimulation, which can be used as a predictive feature in machine learning. Our model predicts several genes as putative ISGs that so far have shown no significant differential expression when stimulated with IFN-α in the cell/tissue types in the available databases. A web server implementing our method is accessible at http://isgpre.cvr.gla.ac.uk/. The docker image at https://hub.docker.com/r/hchai01/isgpre can be downloaded to reproduce the prediction.

## Introduction

**Figure 1:**
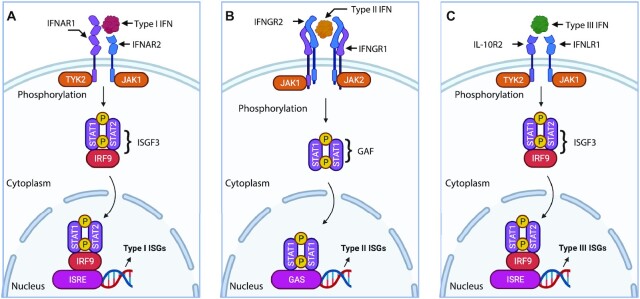
Illustration of signalling cascade triggered by different IFNs. (A) Type I IFN signals through IFNAR, Janus kinase 1 (JAK1), tyrosine kinase 2 (TYK2), STAT, and IFN regulatory factor 9 (IRF9) to form IFN stimulated gene factor 3 complex (ISGF3) and binds to IFN-stimulated response elements (ISRE) to induce the expression of type I ISGs. (B) Type II IFN signals through IFNGR, JAK1, and JAK2 to form IFN-γ activation factor (GAF) and binds to gamma-activated sequence promoter elements (GAS) to induce the expression of type II ISGs. (C) Type III IFN signals through IFNLR1, IL-10R2, JAK1, TYK2, STAT, and IRF9 to form ISGF3 and then binds to ISRE to induce the expression of type III ISGs. Figure created using the BioRender (https://biorender.com/).

Interferons (IFNs) are a family of cytokines defined for their capacity to interfere with viral replication. They are secreted from host cells after an infection by pathogens such as bacteria or viruses to trigger the innate immune response with the aim of inhibiting viral spread by “warning” uninfected cells [1]. The response induced by IFNs is rapid and feedforward, to synthesize new IFNs, which guarantees a full response even if the initial activation is limited [2]. In humans, several IFNs have been discovered (e.g., IFN-α/β/ε/κ/ω/γ/λ [3–8]). IFN-α, IFN-β, IFN-ε, IFN-κ, and IFN-ω are grouped into type I IFNs for signalling through the common IFN-α receptor (IFNAR) complex present on target cells [3–6] (Fig. 1A). IFN-α comprises 13 subtypes in humans while the remaining type I IFNs are encoded by a specific gene [9]. IFN-λ targets IFN-λ receptor 1 (IFNLR1)/interleukin-10 receptor 2 (IL-10R2) and was classified as type III IFN following its discovery in 2003 [8] (Fig. 1C). Similar to type I IFNs, IFN-λ also exerts antiviral properties but functions less intensely [10–12]. IFN-γ is classified as type II IFN and manifests its biological effects by interacting with IFN-γ receptor (IFNGR) [7] (Fig. 1B). In contrast to type I and III IFNs, IFN-γ is also antipathogen, immunomodulatory, and proinflammatory but more focused on establishing cell immunity [3, 7, 11, 13].

All 3 types of IFNs are capable of activating the Janus kinase/signal transducer and activator of transcription (JAK-STAT) pathway and inducing the transcriptional upregulation of approximately 10% of human genes that prime cells for stronger pathogen detections and defence [9, 14, 15]. These upregulated human genes are referred to as IFN-stimulated genes (ISGs). They play an important role in the establishment of the cellular antiviral state, inhibition of viral infection, and return to cellular homeostasis [3, 9, 14, 16]. For example, the ectopic expression of heparinase (HPSE) can inhibit the attachment of multiple viruses [17, 18], interferon-induced transmembrane proteins (IFITM) can impair the entry of multiple viruses and traffic viral particles to degradative lysosomes [19, 20], and MX dynamin-like GTPase proteins (MX) can effectively block early steps of multiple viral replication cycles [21]. Abnormality in the IFN-signalling cascade, for example, the absence of signal transducer and activator of transcription 1 (STAT1), will lead to the failure of activating ISGs, making the host cell highly susceptible to virus infections [22].

Most research on ISGs has focused on elucidating their role in antiviral activities or discovering new ISGs within or across species [3, 9, 14, 19, 23, 24]. The identification of ISGs can be achieved via various approaches. Associating gene expression with suppression of viral infection is a reasonable strategy to identify ISGs with obvious antiviral performance, exemplified by the influenza inhibitor, MX dynamin like GTPase 1 (MX1), and the human immunodeficiency virus 1 inhibitor, MX dynamin-like GTPase 2 (MX2) [21]. CRISPR screening is a loss-of-function experimental approach to identify ISGs required for IFN-mediated inhibition to viruses. It enabled the discovery of tripartite motif containing 5 (TRIM5), MX2, and bone marrow stromal cell antigen 2 (BST2) [25]. Monitoring the ectopic expression of ISGs is another instrumental way to identify ISGs that are individually sufficient for viral suppression [26], for example, interferon-stimulated exonuclease gene 20 (ISG20) and ISG15 ubiquitin-like modifier (ISG15). Using RNA sequencing [27] and fold change–based criteria to measure whether a target human gene is induced by IFN signalling is routinely used [24, 28, 29]. In most cases, a gene is defined as IFN stimulated (upregulated) when its expression value is increased in the presence of IFNs (fold change >2) [3, 24, 30].

There are several online databases to support IFN- or ISG-related research. For example, Interferome [24] provides an excellent resource by compiling *in vivo* and *in vitro* gene expression profiles in the context of IFN stimulation [24]. The Orthologous Clusters of Interferon-stimulated Genes [3, 24] demonstrates an evolutionary comparative approach of genes differentially expressed in the type I IFN system for 10 different species [3].

Experimental data in the Interferome database indicate that a human gene may show differential responses to different IFNs in different tissues or cells [24]. Despite some well-investigated ISGs, the majority of classified ISGs have limited expression following IFN stimulation [3, 24]. This means that the difference between ISGs and those human genes not significantly upregulated in the presence of IFNs (non-ISGs) may not be obvious especially when being assessed more generally. It should also be noted that, within non-ISGs, there are a group of genes downregulated during IFN stimulations. We refer to them as interferon-repressed human genes (IRGs), and they constitute another major part of the IFN regulation system [3, 31]. Collectively, the complex nature of the IFN-stimulated system results in knowledge that is far from comprehensive.

**Figure 2:**
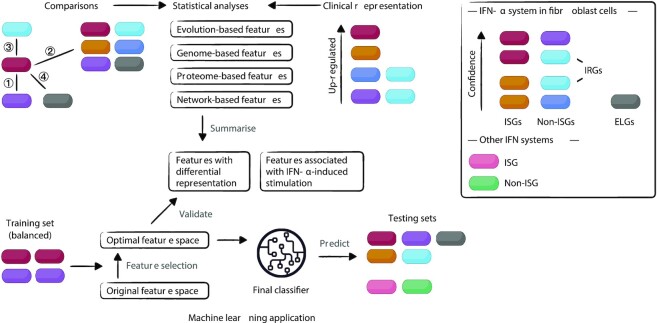
Diagrammatic representation of the project pipeline. Human genes used in analyses and machine learning modelling are classified based on their clinical representations following IFN-α treatment in human fibroblast cells. ISGs (pink block) and non-ISGs (green block) in other IFN systems are only used for testing. The figure is created using images from Wikimedia Commons, https://commons.wikimedia.org.

In this study, we try to associate the inherent properties of human genes with their expression following IFN-α stimulation. We show that it is feasible to make ISG predictions on human genes with a model only compiled from the knowledge of IFN-α responses in the human fibroblast cells. To achieve this, we first constructed a refined high-confidence dataset consisting of 620 ISGs and 874 non-ISGs by checking the genes across multiple databases, including OCISG [3], Interferome [24], and Reference Sequence (RefSeq) [32]. The analyses were conducted primarily on our refined data using genome- and proteome-based features that were likely to influence the expression of human genes in the presence of IFN-α (Fig. 2). Based on the calculated features, we designed a machine learning framework with an optimised feature selection strategy for the prediction of putative ISGs in different IFN systems. Finally, we also developed an online web server and Docker application to implement our machine learning method.

## Results

### Evolutionary characteristics of ISGs

In this study, we constructed dataset S2 from 10,836 well-annotated human genes (dataset S1). It consists of 620 ISGs and 874 non-ISGs with high confidence based on their records in both the OCISG [3] and Interferome [24]. Dataset S1 was used as the background set. Human genes in this set were evolutionarily unrelated to each other as they were retrieved from the OCISG [3]. Detailed information about our compiled datasets is provided in Table 5 and Supplementary Data S1.

**Figure 3:**
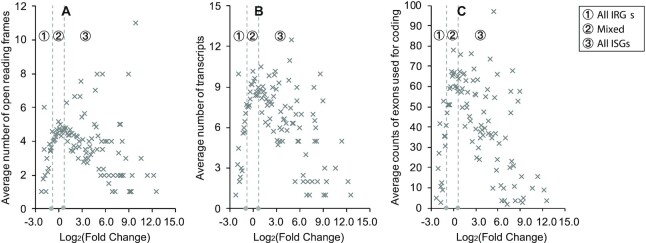
The average representation of alternative splicing features associated with IFN-α stimulations in experiments. (A) The numbers of ORFs and (B) transcripts are used as measurements of the diversity of the alternative splicing process. (C) The count of exons used for coding is used as a measurement of the complexity of alternative splicing process. These 3 plots are drawn based on the expression data of 8,619 human genes with valid fold change in the IFN-α experiments (Supplementary Data S1). The 0.1-length sliding window is adopted to divide the data into 126 bins with different log_2_(fold change). Vertical dashed lines x = −0.871 and x = 0.686 are used to divide the plot into 3 regions. Data points in the left and right regions are produced by IRGs and ISGs, respectively. Data points in the middle region come from ISGs or non-ISGs (including IRGs). A total of 2,217 human genes are not shown in these figures as they had insufficient read coverage to determine a fold change in the experiments (Table 5). Points in the scatterplot are located based on the average feature representation of genes with similar expression performance in experiments.

Here, we explored features relating to alternative splicing [33], duplication [34], and mutation [35]. We found that more highly upregulated human genes tended to have fewer open reading frames (ORFs) (Pearson's correlation coefficient [PCC] = −0.287, Fig. 3A), transcripts (PCC = −0.407, Fig. 3B), and protein-coding exons (PCC = −0.441, Fig. 3C). These results illustrate that alternative splicing may be linked to IFN-α upregulation. Particularly, the data points of IRGs are generally placed below those of non-ISGs, suggesting these 3 features (number of ORFs, number of transcripts, and the usage of protein-coding exons) are all differentially represented in some IRGs compared to the remaining non-ISGs. This distribution also indicates that some IRGs have similar feature properties to ISGs, especially to those highly upregulated in the presence of IFN-α (right part of the scatterplots in Fig. 3A–C).

**Figure 4:**
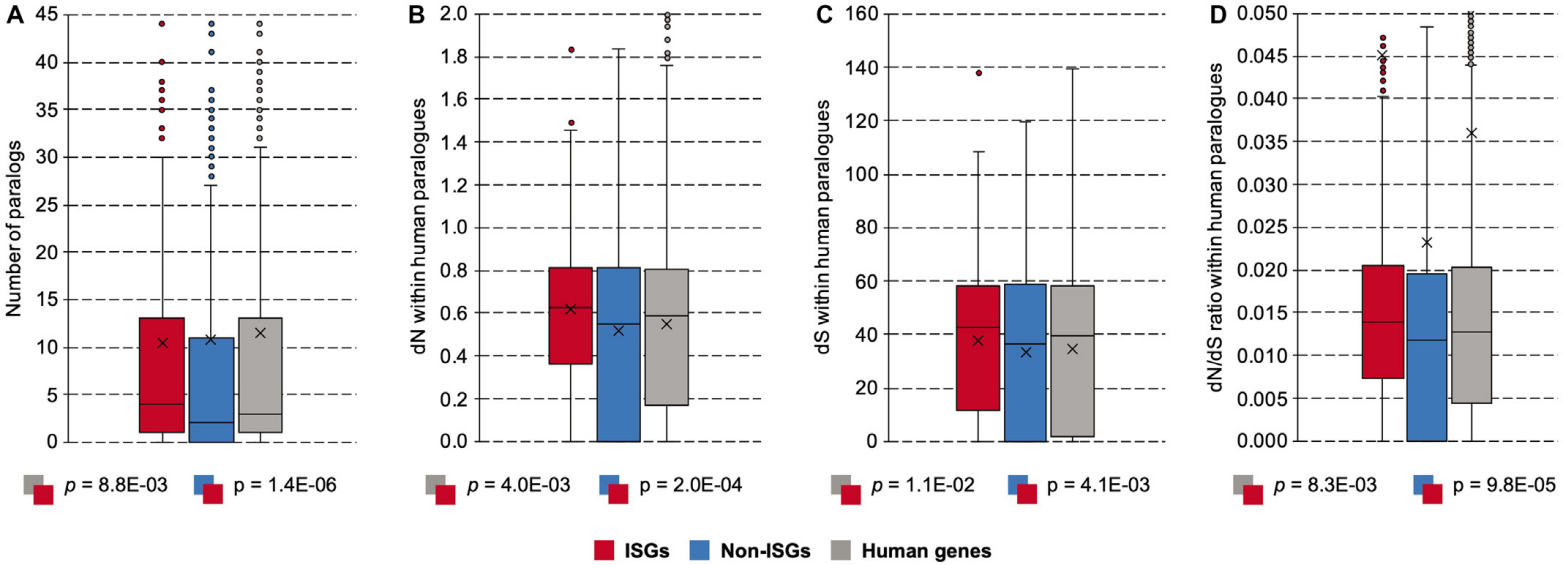
Differences in the evolutionary constraints of human genes. (A) Paralogues within *Homo sapiens*. (B) Nonsynonymous substitutions within human paralogues. (C) Synonymous substitutions within human paralogues. (D) dN/dS ratios within human paralogues. Here, the ISGs and non-ISGs are taken from dataset S2 while the background human genes are from dataset S1 (Table 5). Mann–Whitney *U* tests are applied for the hypothesis testing between the feature distribution of different classes. Boxes in the plot represent the major distribution of values (from the first to the third quartiles); outliers are added for values higher than 2-fold of the third quartile; cross symbol marks the position of the average value, including the outliers; upper and lower whiskers show the maximum and minimum values excluding the outliers.

To determine whether ISGs tend to originate from duplication events, we first counted the number of human paralogues of each gene (Fig. 4A). We found that there were around 22% of singletons in our main dataset, whilst ISGs had 15% and non-ISGs had 26%. The result of a Mann–Whitney *U* test [36] indicated that the number of human paralogues was significantly underrepresented in the ISGs compared to the background human genes (*M*_1_ = 10.5, *M*_2_ = 11.5, *P* = 8.8E-03). Next, we used the number of nonsynonymous substitutions (dN) and synonymous substitutions (dS) within human paralogues to measure the type and strength of selection pressure acting on human genes [37]. As shown in Fig. 4B, nonsynonymous substitutions are more frequently observed in the ISGs than in the background human genes (*M*_1_ = 0.62, *M*_2_ = 0.55, *P* = 4.0E-03). On the other hand, the ISGs tend to have a higher frequency of synonymous substitutions than the background human genes (*M*_1_ = 37.7, *M*_2_ = 34.6, *P* = 1.1E-02) (Fig. 4C), but the difference is not as obvious as for nonsynonymous substitutions. In Fig. 4D, the distribution of dN/dS ratios for human paralogues indicates that most human genes, including ISGs and non-ISGs, are constrained by natural selection, but the ISGs, in general, tend to be moderately less constrained (*M*_1_ = 0.036, *M*_2_ = 0.045, *P* = 8.3E-03). When eliminating the influence of duplication events, the ISGs still receive less selection pressure than the non-ISGs, but the difference in the dN/dS ratio is not significant (*M*_1_ = 0.053, *M*_2_ = 0.031, *P* > 0.05).

### Differences in the coding region of the canonical transcripts

**Figure 5:**
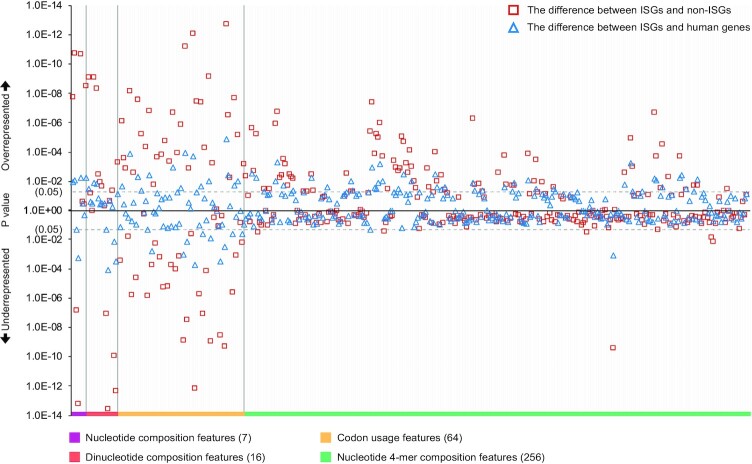
Differences in the representation of discrete features encoded from coding regions (canonical). Mann–Whitney *U* tests are applied for hypothesis testing on the whole comparing data without sampling, and the results are provided in Supplementary Data S2. Here, the ISGs and non-ISGs are taken from dataset S2 (No. = 620 and 874) while the background human genes are from dataset S1 (No. = 10,836) (Table 5).

Compared to general profile features (e.g., number of ORFs), the sequences themselves provide more direct mapping to the protein function and structure [38]. Here, we encoded 344 discrete features and 7,026 categorical features from complementary DNA (cDNA) of the canonical transcript to explore features specific to ISGs. We divided the discrete features into 4 categories (nucleotide composition/dinucleotide composition/codon usage/nucleotide 4-mer composition) and compared their representations among 3 different groups of human genes, including recompiled ISGs from dataset S2, recompiled non-ISGs from dataset S2, and the background human genes from dataset S1 (Fig. 5).

First, guanine and cytosine were both more depleted in ISGs than non-ISGs, leading to an underrepresentation of GC content in the ISGs (Mann–Whitney *U* test: *M*_1_ = 52%, *M*_2_ = 55%, *P* = 2.3E-11). This attribute is the opposite to the GC-biased gene conversion (gBGC) process and would result in ISGs being less stable with weak evolutionary conservation (Fig. 4) [39]. Additionally, the underrepresentation of GC content also influenced the representation of other dinucleotide features. Among all dinucleotide depletions in ISGs, CpG depletion was ranked first followed by GpG and GpC depletions (*P* = 2.9E-14, 4.9E-13, and 1.2E-10, respectively). In turn, adenine- and thymine-related dinucleotide composition, exemplified by ApT and TpA, were more enriched in ISGs than non-ISGs (*P* = 8.0E-10 and 8.5E-10, respectively).

We compared the usage of 64 different codons in the third category as their frequencies influence transcription efficiency [40]. Differences between the ISGs and background human genes were observed in codons for 11 amino acids, including leucine (L), isoleucine (I), valine (V), serine (S), threonine (T), alanine (A), glutamine (Q), lysine (K), glutamic acid (E), arginine (R), and glycine (G). The most significant difference was observed in the usage of codon “AGA.” Among all arginine-targeted alternative codons, codon “AGA” was usually favoured, and its presence reached an estimated 25% in the ISGs but reduced to 22% in the background human genes (*P* = 1.4E-05). It was significantly lower in the non-ISGs, at 18% (*P* = 1.9E-13). On the other hand, compared to the background human genes, the codon “CAG” coding for amino acid “Q” was the most underrepresented in the ISGs. It was less favoured by the ISGs than non-ISGs (*M*_1_ = 72%, *M*_2_ = 78%, *P =*7.3E-13), although it dominated in coding patterns. As for the 3 stop codons, compared with the background human genes, the usage of the TAA stop codon was overrepresented in the ISGs (*M*_1_ = 28%, *M*_2_ = 33%, *P =*9.7E-03). In this category of codon usage, the features with different frequencies between the ISGs and background human genes became more discriminating when comparing the ISGs with non-ISGs. Significant differences in codon usages between the ISGs and non-ISGs were widely observed except for methionine (M) and tryptophan (W). Hence, despite the limited differences of codon usages between the ISGs and background human genes, these features were useful for discriminating the ISGs from non-ISGs.

In the last category, we calculated the occurrence frequency of 256 nucleotide 4-mers to add some positional resolution for finding and comparing interesting organisational structures [41]. Among the 256 4-mers, 46 of them were differentially represented between the ISGs and background human genes (Supplementary Data S2). Most of these 4-mers were overrepresented by the ISGs except 2 with the pattern “TAAA” and “CGCG.” Interestingly, the feature of “TAAA” composition became a positive factor when comparing ISGs and non-ISGs (*M*_1_ = 4.1%, *M*_2_ = 3.7%, *P =*4.1E-06), suggesting it might be a suitable feature to discern potential or incorrectly labelled ISGs. We found that 6 nucleotide 4-mers (“ACCC,” “AGTC,” “AGTG,” “TGCT,” “GACC,” and “GTGC”) were overrepresented in the ISGs when compared to the background human genes. However, they were not differentially represented when comparing the ISGs with non-ISGs. These 6 features might be inherently biased for some reason and were not powerful enough to contribute to distinguishing the ISGs from non-ISGs. In addition to the aforementioned 40 features (except 4-mer “ACCC,” “AGTC,” “AGTG,” “TGCT,” “GACC,” and “GTGC”) that were differentially represented in ISGs compared to background human genes, we found a further 39 features that nucleotide 4-mers differentially represented between ISGs and non-ISGs (Supplementary Data S2).

To check the effect of these aforementioned 343 features on the level of stimulation in the IFN-α system (log_2_(fold change) >0), we calculated the PCC for the normalised features (Eq. 2) and found 106 features were positively related to the increase of fold change, and 34 features were suppressed when human genes were more upregulated after IFN-α treatments (Student *t*-test: *P* < 0.05) (Supplementary Data S3). ApA composition showed the most obvious positive correlation with stimulation level (PCC = 0.464, *P =*8.8E-06), while a negative association between the representation of 4-mer “CGCG” and IFN-α–induced upregulation was the most significant (PCC = −0.593, *P =*3.2E-09). Human genes with higher upregulation in the presence of IFN-α contained more codons “CAA,” rather than “CAG” for coding amino acid “Q.” The depletion of GC content, especially cytosine content, promotes the suppression of many nucleotide compositions in the cDNA (e.g., CpG composition).

**Figure 6:**
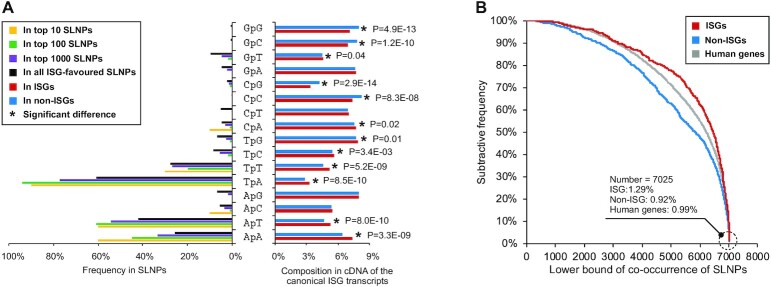
Short linear nucleotide patterns (SLNPs) in the coding regions (canonical). (A) Influence of dinucleotide composition on the flagged SLNPs. (B) The co-occurrence status of SLNPs in different human genes. Ranks in (A) are generated based on the adjusted *P*-value given by Pearson's chi-squared tests after the Benjamini–Hochberg correction procedure. Detailed results of the hypothesis tests are provided in Supplementary Data S4. Here, the ISGs and non-ISGs are taken from dataset S2 while the background human genes are from dataset S1 (Table 5).

To find conserved sequence patterns relating to gene regulation [42], we checked the existence of 2,940, 44,100, and 661,500 short linear nucleotide patterns (SLNPs) consisting of 3 to 5 consecutive nucleobases in the group of the ISGs and non-ISGs. By using a positive 5% difference in the occurrence frequency as the cutoff threshold, we found 7,884 SLNPs with a maximum difference in representation of around 15%. After using Pearson's chi-squared tests and Benjamini–Hochberg correction to avoid type I error in multiple hypotheses [43], 7,025 SLNPs remained with an adjusted *P*-value lower than 0.01 (Supplementary Data S4), hereon referred to as “flagged” SLNPs. The differentially represented 7,025 SLNPs were ranked according to the adjusted *P*-value. As shown in Fig. 6A, dinucleotide “TpA” dominates in the top 10, top 100, top 1,000, and all differentially represented SLNPs even if TpA representation is suppressed in the cDNA of genes’ canonical transcripts compared to other dinucleotides. Dinucleotide “ApT” and “ApA” are also frequently observed in the flagged SLNPs, but their occurrences do not show significant differences in the top 100 SLNPs (Pearson's chi-squared test: *P* > 0.05). GC-related dinucleotides (e.g., “CpC,” “GpC,” and “GpG”) are rarely observed in the flagged SLNPs, especially in the top 10 or top 100. In view of this, we hypothesize that the differential representation of nucleotide compositions influences and reflects on the pattern of SLNPs in the ISGs. By checking the co-occurrence status of the flagged SLNPs, we found that these sequence patterns had a cumulative effect in distinguishing the ISGs from non-ISGs, especially when the number of co-occurring SLNPs reached around 5,320 (Pearson's chi-squared test: *P =*7.9E-13, Fig. 6B). There were 8 (∼1.3%) ISGs in dataset S2 containing all the flagged 7,025 SLNPs. Their upregulations after IFN-α treatment were generally low with a fold change fluctuating around 2.2. However, some of these 8 genes, such as desmoplakin (DSP), were clearly highly upregulated in endothelial cells isolated from human umbilical cord veins after not only IFN-α treatments (fold change = 11.1) but also IFN-β treatments (fold change = 13.7). We also found some non-ISGs (e.g., hemicentin 1 [HMCN1]) and human genes with limited expression in the IFN-α experiments (ELGs) (e.g., tudor domain containing 6 [TDRD6]) containing the flagged SLNPs, but their frequencies were lower than that in the ISGs.

### Differences in the protein amino acid sequence

**Figure 7:**
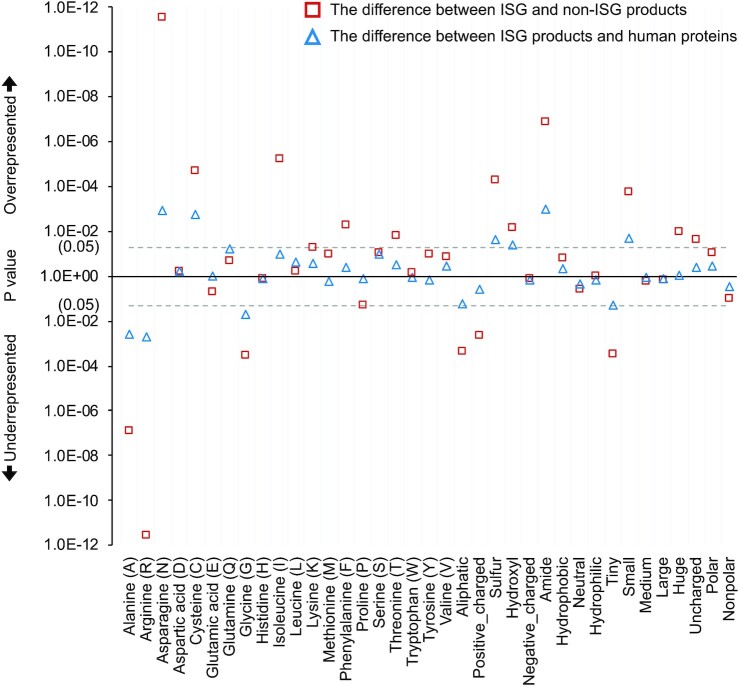
Differences in the representation of discrete features encoded from protein sequences. Mann–Whitney *U* tests are applied for hypothesis testing on the whole data without sampling and the results are provided in Supplementary Data S2. Here, the ISGs and non-ISGs are taken from dataset S2 (No. = 620 and 874) while the background human genes are from dataset S1 (No. = 10,836) (Table 5). Aliphatic group: amino acids “A,” “G,” “I,” “L,” “P,” and “V”; aromatic/huge group: amino acids “F,” “W,” and “Y” (volume >180 cubic angstroms); sulphur group: amino acids “C” and “M”; hydroxyl group: amino acids “S” and “T”; acidic/negative_charged group: amino acids “D” and “E”; amide group: amino acids “N” and “Q”; positive_charged group: amino acids “R,” “H,” and “K”; hydrophobic group: amino acids “A,” “C,” “I,” “L,” “M,” “F,” “V,” and “W” that participate in the hydrophobic core of the structural domains [46]; neutral group: amino acids “G,” “H,” “P,” “S,” “T,” and “Y”; hydrophilic group: amino acids “R,” “N,” “D,” “Q,” “E,” and “K”; tiny group: amino acids “G,” “A,” and “S” (volume <90 cubic angstroms); small group: amino acids “N,” “D,” “C,” “P,” and “T” (volume ranged from 109 to 116 cubic angstroms); medium group: amino acids “Q,” “E,” “H,” and “V” (volume ranged within 138 to 153 cubic angstroms); large group: amino acids “R,” “I,” “L,” “K,” and “M” (volume ranged within 163 to 173 cubic angstroms); uncharged group: the remaining 15 amino acids except electrically charged ones; polar group: amino acids “R,” “H,” “K,” “D,” “E,” “N,” “Q,” “S,” “T,” and “Y”; nonpolar group: the remaining 10 amino acids except polar ones.

We used the amino acid sequences generated by the canonical transcript to extract features at the proteomic level. In addition to the basic composition of 20 standard amino acids, we considered 17 additional features related to physicochemical (e.g., hydropathy and polarity) or geometric properties (e.g., volume) [44, 45]. We found several amino acids that were either enriched or depleted in the ISG products compared to the background human proteins, which were produced by genes in dataset S1 (Fig. 7). The differences were even more marked between protein products of the ISGs and non-ISGs, highlighting some differences that were not observed when comparing the ISG products to the background human proteins (e.g., isoleucine composition). The differences observed in the amino acid composition were at least in part associated with the patterns previously observed in features encoded from genetic coding regions. For example, asparagine (N) showed significant overrepresentation in the ISG products compared to the non-ISG products or background human proteins (Mann–Whitney *U* test: *P =*2.8E-12 and 1.2E-03, respectively). This was expected as there are only 2 codons (i.e., “AAT” and “AAC” coding for amino acid “N”), and dinucleotide “ApA” showed a remarkable enrichment in the coding region of ISGs. A similar explanation could be given for the relationship between the deficiency of GpG content and amino acid “G.” The translation of amino acid “K” was also influenced by ApA composition but was not significant due to the mild representation of dinucleotide “ApG” in the genetic coding region. Additionally, as previously mentioned, the ISGs showed a significant depletion in the CpG content, and consequently, the amino acids “A” and “R” in the ISG products were significantly underrepresented. Cysteine (C) was not frequently observed in human proteins but still showed a relatively significant enrichment in the ISG products (*M*_1_ = 2.3%, *M*_2_ = 2.5%, *P =*1.8E-03).

When focusing on the composition of amino acid sequences grouped by physicochemical or geometric properties, we found some features differentially represented between the ISG products and background human proteins. The result showed that hydroxyl (amino acids “S” and “T”), amide (amino acids “N” and “Q”), or sulphur amino acids (amino acids “C” and “M”) were more abundant in the ISG products compared to the background human proteins (Mann–Whitney *U* test: *P =*0.04, 1.0E-03, and 0.02, respectively). Small amino acids (amino acids “N,” “C,” and “T”; aspartic acid [D]; and proline [P]; the volume ranging from 108.5 to 116.1 cubic angstroms) were more frequently observed in the ISG products than in background human proteins (*M*_1_ = 22.1%, *M*_2_ = 21.7%, *P =*0.02). These differences became more marked when comparing the representation of these features between the ISG and non-ISG products. For example, features relating to chemical properties of the side chain (e.g., aliphatic), charge status, and geometric volume showed differences between proteins produced by the ISGs and non-ISGs. Some features such as neutral amino acids that include amino acids “G,” “P,” “S,” and “T”; histidine (H); and tyrosine (Y) were not differentially represented between the ISG and non-ISG products, but they indicated an obvious association with the change of IFN-α–triggered stimulations (PCC = −0.556, *P =*4.1E-08) (Supplementary Data S3).

**Figure 8:**
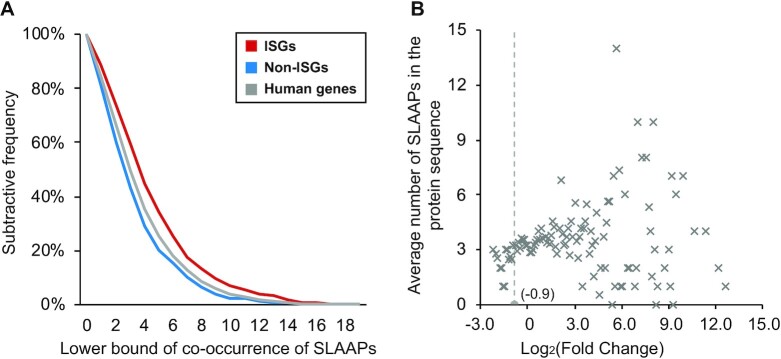
Representation of co-occurring short linear amino acid patterns (SLAAPs, flagged), in our main dataset. (A) The co-occurrence status of SLAAPs in different classes. (B) Relationship between co-occurrence of the marked SLAAPs and log_2_(fold change) after IFN-α treatments. Here, the ISGs and non-ISGs are taken from dataset S2 while the background human genes are from dataset S1 (Table 5). Points in (B) are located based on the average feature representation of genes with similar expression performance in IFN-α experiments.

Next, we searched the sequence of the ISG products against that of the non-ISG products to find conserved short linear amino acid patterns (SLAAPs), which might be constrained by strong purifying selection [47]. As opposed to the analysis of the genetic sequence, we obtained only 19 enriched sequence patterns with a Pearson's chi-squared *P*-value ranging from 1.5E-04 to 0.02 (Table 1), hereon referred to as flagged SLAAPs. They were greatly influenced by 4 polar amino acids, “K,” “N,” “E,” and “S,” and 1 nonpolar amino acid: “L.” Some of these flagged SLAAPs (e.g., SLAAP “NVT” and “S-N-E”) were clearly overrepresented in the ISG products compared to the background human proteins and could be used as features to differentiate the ISGs from background human genes. The third column in Table 1 indicates a number of patterns that are lacking in the non-ISG products and hence may be the reason for the lack of upregulation in the presence of IFN-α. Particularly, we noticed that SLAAP “KEN” was a destruction motif that could be recognised or targeted by anaphase promoting complex (APC) for polyubiquitination and proteasome-mediated degradation [48, 49]. Results shown in Fig. 8A illustrate that the co-occurrence of differentially represented SLAAPs (flagged) has a cumulative effect in distinguishing the ISGs from non-ISGs. This cumulative effect can even be achieved with only 2 random SLAAPs (Pearson's chi-squared test: *P =*4.6E-10). The bias in the co-occurring SLAAPs (flagged) in the background human proteins towards a pattern similar to the non-ISG products further proves the importance of these 19 SLAAPs. However, their co-occurrence is not associated with the level of IFN-triggered stimulations (PCC = 0.015, *P* > 0.05) (Fig. 8B).

**Table 1:** Representation of SLAAPs in protein sequences and their IDRs

SLAAP^a^	Frequency in ISG/non-ISG products^b^	Bias based on the frequency in human proteins	*P-* value^c^	Conditional frequency in the IDRs of ISG/non-ISG products/background human proteins^c,d^	*P-* value^e^
SxNxE	15.2%/8.8%	+47.6%/−14.2%	1.5E-04	39.4%/40.3%/33.4%	0.90
ENE	15.0%/8.8%	+20.9%/−29.0%	2.1E-04	37.6%/42.9%/40.9%	0.49
SxNxT	11.5%/6.2%	+21.9%/−34.2%	2.9E-04	40.8%/25.9%/27.3%	0.08
SVI	15.2%/9.2%	+37.6%/−16.9%	3.6E-04	18.1%/11.3%/15.2%	0.21
LxNL	23.7%/16.4%	+13.2%/−21.9%	4.0E-04	10.2%/11.9%/9.4%	0.65
LxKL	30.8%/22.8%	+18.0%/−12.8%	4.9E-04	12.6%/10.1%/8.7%	0.43
NVT	13.7%/8.5%	+52.1%/−6.1%	1.2E-03	18.8%/21.6%/15.4%	0.66
ISS	20.5%/14.3%	+20.7%/−15.7%	1.7E-03	29.9%/25.6%/23.8%	0.44
LKxK	24.4%/17.7%	+24.5%/−9.3%	1.8E-03	14.6%/20.6%/20.0%	0.16
IKxE	14.2%/9.0%	+34.2%/−14.5%	1.8E-03	26.1%/16.5%/25.8%	0.13
EKxI	15.8%/10.4%	+31.0%/−13.7%	2.0E-03	15.3%/20.9%/16.0%	0.32
KxExS	16.9%/11.4%	+21.9%/−17.7%	2.4E-03	36.2%/36.0%/39.2%	0.98
LNS	17.7%/12.1%	+21.2%/−17.1%	2.4E-03	20.0%/25.5%/20.5%	0.34
KEN	16.0%/10.6%	+33.5%/−11.0%	2.4E-03	27.3%/41.9%/34.8%	0.03
LxNxL	22.6%/17.5%	+14.3%/−11.4%	1.5E-02	10.7%/11.8%/9.5%	0.78
KxExL	25.8%/20.5%	+25.7%/−0.3%	1.5E-02	18.8%/17.9%/18.7%	0.84
KLL	27.1%/21.9%	+9.9%/−11.4%	1.9E-02	11.3%/8.4%/9.9%	0.35
LKE	29.8%/24.5%	+18.2%/−3.0%	2.1E-02	19.5%/24.8%/20.1%	0.20
LKxL	33.2%/27.7%	+15.0%/−4.2%	2.1E-02	7.8%/12.4%/10.0%	0.11

a “x” in SLAAPs indicates 1 position occupied by a standard amino acid.

b Here, the ISGs and non-ISGs are taken from dataset S2 while the background human genes use samples from dataset S1 (Table 5).

c *P-*values in this column use Pearson's chi-squared tests to measure the difference of SLAAP occurrences in the ISG and non-ISG products.

d Frequencies in this column are calculated based on a condition that corresponding SLAAPs are observed in the protein sequence.

e *P*-values in this column use Pearson's chi-squared tests to measure the difference of SLAAP occurrences in the IDRs of the ISG and non-ISG products.

Regions that lacked stable structures under normal physiological conditions within proteins are termed *intrinsically disordered regions* (IDRs). They play an important role in cell signalling [50]. Compared with ordered regions, IDRs are usually more accessible and have multiple binding motifs, which can potentially bind to multiple partners [51]. According to the results calculated by IUPred [52], we identified 6,721, 10,510, and 119,071 IDRs (IUpred score no less than 0.5) in proteins produced by the ISGs, non-ISGs, and background human genes, respectively. We hypothesize that enriched SLAAPs widely detected in the IDRs may be important for human protein–protein interactions or potentially virus mimicry [53]. For instance, in the ISG products, about 40.8% of SLAAP “SxNxT” were observed in the IDRs, 14.9% higher than that in non-ISG products (Table 1). This difference reflected the importance of SLAAP “SxNxT” for target specificity of IFN-α–induced protein–protein interactions (PPIs) [9], even if it was not statistically significant. By contrast, the conditional frequency of SLAAP “SxNxE” in the IDRs of the ISG and non-ISG products was almost the same, indicating that SLAAP “SxNxE” might have an association with some inherent attributes of the ISGs but was less likely to be involved in the IFN-α–induced PPIs. SLAAP “KEN” in the IDRs also showed some interesting differences: in the non-ISG products, 41.9% of SLAAP “KEN” were observed in the IDRs, 14.6% higher than that in the ISG products, which provided an effective approach to distinguish the ISGs from non-ISGs. When SLAAP “KEN” is discovered in the ordered globular region of a protein sequence, statistically, the protein is more likely to be produced by an ISG, but this assumption is reversed if the SLAAP is located in an IDR (Pearson's chi-squared tests: *P =*0.03). Despite the relatively low conditional frequency of SLAAP “KEN” in the IDRs of the ISG products, these SLAAPs in the IDR are more likely to be functionally active than those falling within ordered globular regions [54].

### Differences in network profiles

We constructed a network with 332,698 experimentally verified interactions among 17,603 human proteins (confidence score >0.63) from the Human Integrated Protein–Protein Interaction rEference (HIPPIE) database [55] to investigate if the connectivity among human proteins has an association with genes’ expression in the IFN-α experiments. In total, 10,169 out of 10,836 human proteins produced by genes in our background dataset S1 were included in the network. Based on this network, we calculated 8 features as defined in the methods, including the average shortest path, closeness, betweenness, stress, degree, neighbourhood connectivity, clustering coefficient, and topological coefficient.

**Figure 9:**
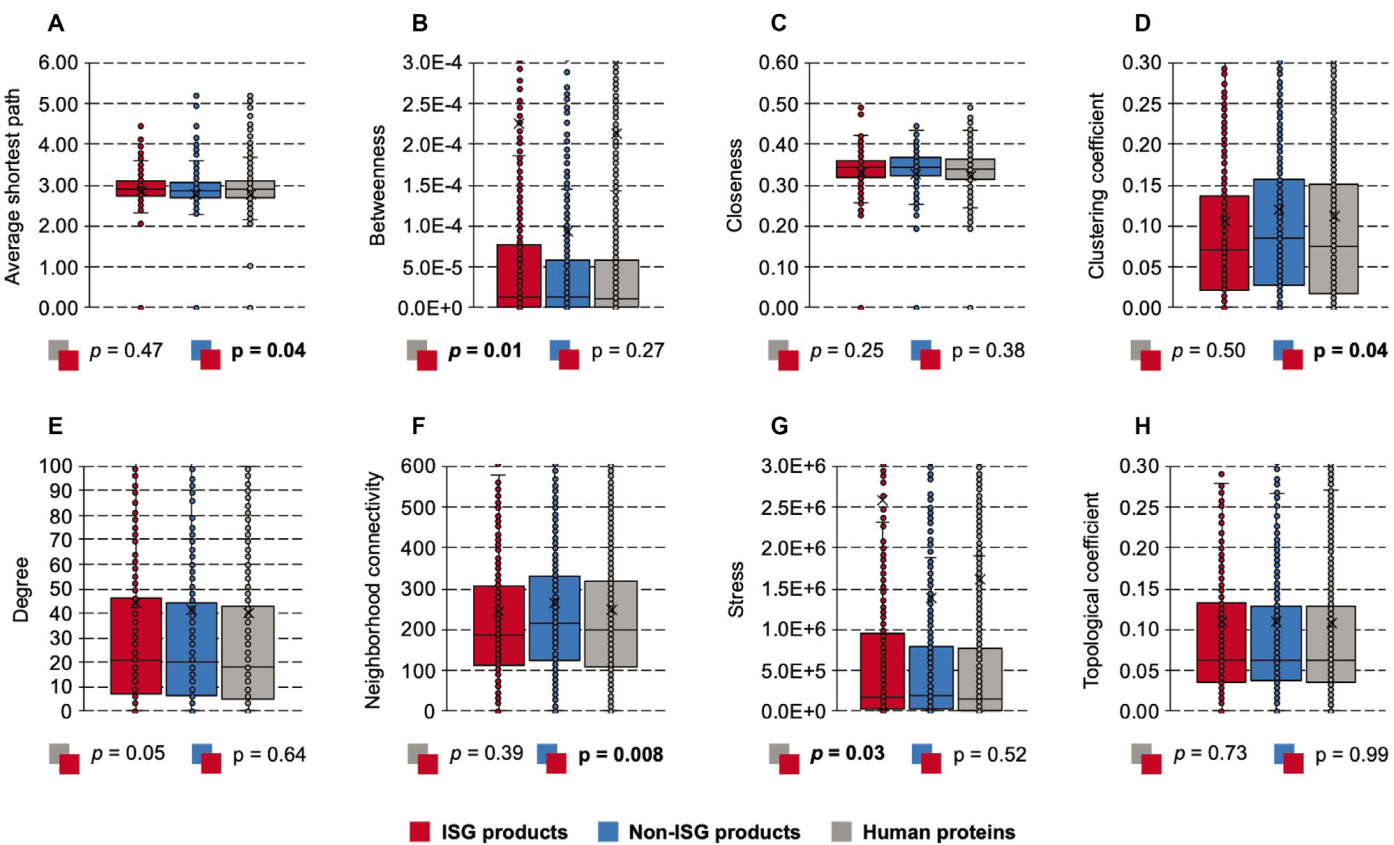
Differences in network properties. The included features are (A) average shortest path, (B) betweenness, (C) closeness, (D) clustering coefficient, (E) degree, (F) neighbourhood connectivity, (G) stress, and (H) topological coefficient. Mann–Whitney *U* tests are applied for hypothesis testing on the whole comparing data without sampling, and the results are provided in Supplementary Data S2. Here, the ISGs and non-ISGs are taken from dataset S2 (No. = 620 and 874) while the background human genes use samples from dataset S1 (No. = 10,836) (Table 5).

As illustrated in Fig. 9B and G, ISG products tend to have higher values of betweenness and stress than background human proteins (Mann–Whitney *U* test: *P =*0.01 and 0.03, respectively), which means they are more likely to locate at key paths connecting different nodes of the PPI network. Some ISG products with high values of betweenness and stress (e.g., tripartite motif containing 25 [TRIM25]) can be considered the shortcut or bottleneck of the network and play important roles in many PPIs, including those related to the IFN-α–triggered immune activities [56, 57]. However, such differential representation of betweenness does not mean ISG products are more likely to be or even be close to bottlenecks of the network compared to the background human proteins. Some examples shown in Table 2 indicate that ISG products are less connected by top-ranked bottlenecks and hubs of the network than non-ISG products or the background human proteins. This conclusion is not influenced by the hub/bottleneck protein's performance in the IFN-α experiments. Comparing proteins produced by the ISGs and non-ISGs, we found the former tends to have lower values of clustering coefficient and neighbourhood connectivity (Mann–Whitney *U* test: *P =*0.04 and 7.9E-03, Fig. 9D and F). This discovery indicates that the ISG products and some of their interacting proteins are less likely to be targeted by lots of proteins. It also supports the finding that the ISG products are involved in many shortest paths for nodes but are away from hubs or bottlenecks in the network. To some extent, this location also increases the length of the average shortest paths through ISG products in the network (Fig. 9A).

**Table 2:** Interaction profiles of human proteins connecting top hubs/bottlenecks of the HIPPIE network

Human protein	TRIM25	ELAVL1	ESR2	NTRK1
Gene class	ISG	IRG	Not included in S1^a^	
Degree (hub rank)	2295 (2nd)	1787 (4th)	2500 (1st)	1976 (3rd)
Betweenness (bottleneck rank)	0.067 (1st)	0.048 (4th)	0.051 (3rd)	0.026 (5th)
Difference in interacting partners	Depleted	*P* > 0.05	Depleted	Depleted
(ISG products versus non-ISG products)^b^	*P* = 0.01		*P* = 1.1E-4	*P* = 5.5E-3
Difference in interacting partners	*P* > 0.05	*P* > 0.05	Depleted	Depleted
(ISG products versus the background human proteins)^b^			*P* = 8.1E-3	*P* = 0.03

a ESR2 and NTRK1 were not included in dataset S1 as their expression data were not compiled in OCISG.

b Differences here are measured via Pearson's chi-squared tests on human proteins interacting with the corresponding hub/bottleneck protein.

When investigating the association between IFN-α–induced gene stimulation and network attributes of gene products, we only found the feature of neighbourhood connectivity was underrepresented as the level of differential expression in the presence of IFN increases (PCC = −0.392, *P =*2.2E-04). This suggests that proteins produced by genes that are highly upregulated in response to IFN-α are further away from hubs in the PPI networks.

### Features highly associated with the level of IFN stimulations

**Figure 10:**
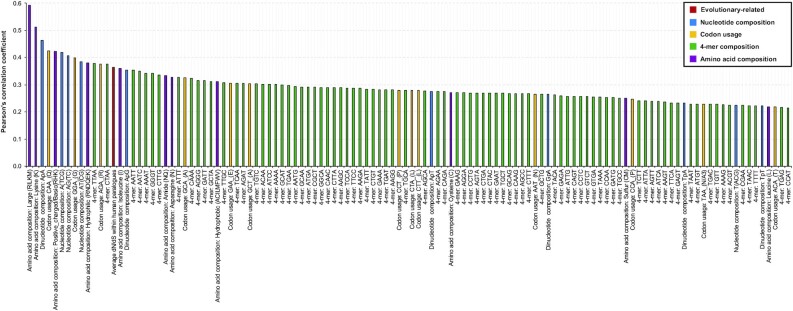
A total of 118 features positively associated with higher upregulation after IFN-α treatments. Features here are screened based on Pearson’s correlation coefficient (PCC) and results of Student t-tests (*P* < 0.05). Features with a higher PCC indicate a stronger positive correlation. Detailed results about PCC and hypothesis tests are provided in Supplementary Data S3.

**Figure 11:**
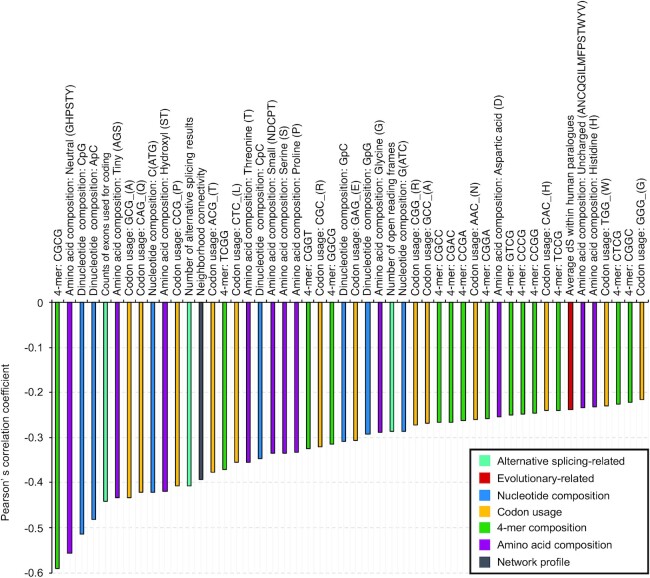
Fifty features negatively associated with higher upregulation after IFN-α treatments. Features here are screened based on Pearson’s correlation coefficient (PCC) and results of Student *t*-tests (*P* < 0.05). Features with a lower PCC indicate stronger negative correlation. Detailed results about PCC and hypothesis tests are provided in Supplementary Data S3.

In this study, we encoded a total of 397 discrete and 7,046 categorical features covering the aspects of evolutionary conservation, nucleotide composition, transcription, amino acid composition, and network profiles. In order to find out some key factors that may enhance or suppress the stimulation of human genes in the IFN-α system, we compared the representation of discrete features of human genes with different but positive log_2_(fold change). Two features on the co-occurrence of SLNPs and SLAAPs were not taken into consideration here as they were more subjective than the other discrete features and were greatly influenced by the number of sequence patterns. Upon the calculation of PCC and the result of hypothesis tests, we found 168 features highly associated with the level of IFN-α–triggered stimulations (Student *t*-tests: *P* < 0.05) (Supplementary Data S3). Among them, 118 features showed a positive correlation (Fig. 10) while the remaining 50 features showed a negative correlation (Fig. 11) with the change of upregulation in IFN-α experiments. Among these 168 features, the number of ORFs, alternative splicing results, and counts of exons used for coding were encoded from characteristics of the gene. Average dN/dS and average dS within human paralogues were encoded based on the sequence alignment results from Ensembl [58]. In total, 140 and 22 features were encoded from the genetic sequence and proteomic sequence, respectively. The last one, neighbourhood connectivity, was obtained from the network profile of a human interactome constructed based on experimentally verified data in the HIPPIE database [55].

In the positive group, “large” amino acid composition that includes the composition of 5 amino acids with geometric volume ranging from 163 to 173 cubic angstroms was ranked first for having the highest PCC at 0.593 (Student *t*-test: *P =*2.8E-09). This feature was not highlighted previously as it did not have a strong signal for discriminating the ISGs from non-ISGs (Mann–Whitney *U* test: *P* > 0.05). Similar phenomena were found on 87 features (64 positive correlations and 23 negative correlations) such as AG content, ApG content, and previously mentioned neutral amino acid composition. The strongest negative correlation between feature representation and IFN-α–triggered stimulations was found on the feature of 4-mer “CGCG” (PCC = −0.593, *P =*3.2E-09). This feature also showed a differential distribution between the ISGs and non-ISGs, providing useful information to distinguish the ISGs from non-ISGs. Similar phenomena were found on 81 features (54 positive correlations and 27 negative correlations) such as previously mentioned GC content, CpG content, and the usage of codon “GCG” coding for amino acid “A.”

Collectively, the biased effect on the basic composition of nucleotide sequences influences the correlation between the representation of sequence-based features and IFN-α–triggered stimulations. Human genes that show overrepresentation in more features listed in Fig. 10 are expected to be more upregulated after IFN-α treatments at least in the human fibroblast cells. Meanwhile, the underrepresentation of features listed in Fig. 11 also contributes to the level of upregulation in the IFN-α experiments.

### Difference in feature representation of interferon-repressed genes and genes with low levels of expression

We grouped human genes into 2 classes based on their response to IFN-α in the human fibroblast cells. Genes significantly upregulated in IFN-α experiments were included in the ISG class, while those that did not were put into the non-ISG class. However, there is also another group of human genes downregulated in the presence of IFN-α (i.e., the IRGs). They were labelled as the non-ISGs but contain unique patterns that constitute an important aspect of the IFN response [3]. Some of these IRGs were not upregulated in any known type I IFN systems, and thus they have been placed in a refined non-ISG class for analyses and predictions. Additionally, a number of genes have insufficient levels of expression in the experiments to determine a fold change (i.e., ELGs). Here, we used the previously defined features to compare the ISGs from dataset S2 with the IRGs and ELGs divided from the background dataset S1 (Table 5).

**Figure 12:**
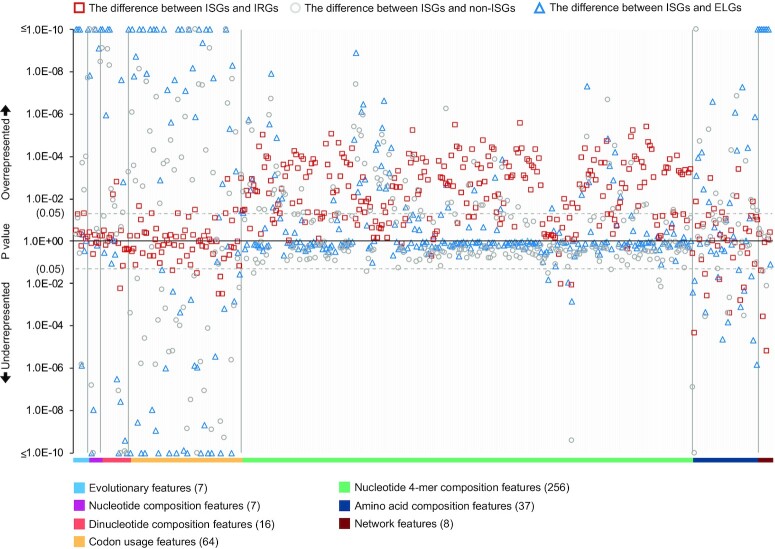
Differential expressions of discrete features between different genes and their coded proteins. Mann–Whitney *U* tests are applied for hypothesis testing on the whole comparing data without sampling, and the results are provided in Supplementary Data S2. Here, the ISGs and non-ISGs are taken from dataset S2 (No. = 620 and 874); the IRGs and ELGs are taken from dataset S4 (No. = 1,006) and dataset S8 (No. = 2,217); the background human genes are from dataset S1 (No. = 10,836) (Table 5).

As shown in Fig. 12, the IRGs are differentially represented to a lower extent in the majority of nucleotide 4-mer composition features than the ISGs, indicating the deficiency of some nucleotide sequence patterns in the coding region of IRGs. Note that many nucleotide 4-mer composition features are more suppressed in the ISGs than non-ISGs, although the differences are small. The biased representation of these features in the IRGs suggests that the IRGs have characteristics similar to the ISGs rather than non-ISGs. Additionally, there are a very limited number of features relating to evolutionary conservation, nucleotide sequence composition, or codon usage showing obvious differences between the ISGs and IRGs, but many of them are differentially represented when comparing the ISGs with non-ISGs. Therefore, involving the IRGs in the class of the non-ISGs will increase the risk for machine learning models to produce more false positives. However, there are some informative features differentiating the IRGs from ISGs. For example, compared to the ISGs, the IRGs are more enriched in CpGs (Mann–Whitney *U* test: *P =*5.6E-03), which is also mentioned in [59]. The IRGs tend to have higher closeness centrality and neighbourhood connectivity than the ISGs (Mann–Whitney *U* test: *P =*0.04 and 6.4E-06, respectively), suggesting that the IRGs are more central in the human PPI network and connected to key proteins with many interaction partners. Differences in some amino acid composition features between the ISGs and IRGs can also be observed in Fig. 12. Therefore, accurate predictability is still expected when using features extracted from protein sequences.

Figure 12 illustrates 161 features showing significant differences (Mann–Whitney *U* tests: *P* < 0.05) in the representation of the ISGs and ELGs. An estimated 82% of these features were also differentially represented between the ISGs and non-ISGs. Seventy-nine percent of these significant features displayed similar overrepresentation or underrepresentation in 2 comparisons: ISGs versus ELGs and ISGs versus non-ISGs. These ratios indicate that the majority of the ELGs are less likely to be ISGs based on their feature profile as well as their low expression levels in cells induced with IFN-α. Network analyses showed that the ELG products tended to have lower values of all calculated network features than ISG products, with the exception of topological coefficient. This means that the ELG products are less connected to other human proteins in the human PPI network. Particularly, their abnormal representation on the feature of average shortest paths indicates that some ELGs (e.g., vascular cell adhesion molecule 1 [VCAM1] and ubiquitin D [UBD]) may still have high connectivity in the human PPI network.

### Implementation with machine learning framework

In this study, we encoded 397 discrete and 7,046 categorical features for the analyses. As excess of features will greatly increase the dimension of feature spaces and complicate the classification task for the classifier, we limited the number of SLNPs to the top 100 based on the adjusted *P*-value, and we expected these to be sufficient to provide a picture of short linear sequence patterns in the coding region of the canonical transcript. Accordingly, features measuring the co-occurrence status of multiple SLNPs were recalculated based on the selected 100 SLNPs. As a result, we prepared 518 features (Supplementary Data S5) for our machine learning framework. To reduce the impact of noisy data on classifications, we used only the refined ISGs and non-ISGs from dataset S2 for training and modelling.

Table 3 first shows the comparisons of prediction performance among different machine learning methods. The threshold is determined by maximising the value of the Matthews correlation coefficient (MCC). As the random forest (RF) classifier was built based on randomly selected samples and features [60], we repeated its modelling procedure 10 times. These initial comparisons showed that the support vector machine (SVM) [61] is superior to k-nearest neighbours (KNN) and RF [60]. The poor prediction performance of the best base classifier (SVM, area under the curve [AUC] = 0.6509) indicates that there are a number of poorly performing features hidden in the set. As some genes respond to IFNs in a cell-specific manner [2], it is hard to produce predictions unless we detect key discriminating features, which are robust to the change of biological environment.

**Table 3:** Performance of different machine learning classifiers on the training dataset S2′ via 5-fold cross-validation

Classifier	Method	Features	Threshold dependent					Threshold independent	
			Score range	Threshold^a^	Sensitivity	Specificity	MCC	SN_496^b^	AUC
Basic	KNN^c^	518	0.100∼0.900	0.500∼0.550	0.593	0.621	0.214	0.607 ± 0.014	0.6305
	RF^d^	Random	0.080∼0.900	0.380∼0.579	0.590 ± 0.168	0.617 ± 0.183	0.219 ± 0.019	0.600 ± 0.007	0.6413 ± 0.0082
	SVM	518	0.328∼0.743	0.542	0.567	0.681	0.250	0.615	0.6509
Optimised	SVM + FFS	78^e^	0.170∼0.836	0.561	0.518	0.760	0.287	0.621	0.6768
	SVM + ASI	74^e^	0.098∼0.918	0.549	0.623	0.750	0.376	0.681	0.7479

a This threshold is provided by maximising the value of MCC.

b This sensitivity is measured among tested genes with the top 496 prediction probabilities.

c The k-value here is set as the square root of the size of the training samples in 5-fold cross validation (i.e., k = 20) [62].

d This random forest algorithm uses 50 random grown trees and the modelling and validation procedures are repeated 10 times.

e These features constitute the best/optimum feature set for the current machine learning method.

Here, we considered 2 iterative strategies for selecting predictive features. The first one is the forward feature selection (FFS) [63] wherein features are added sequentially based on their individual performance. This strategy did not work well (Table 3) as the prediction performances were all poor when the feature was used individually (Supplementary Data S5). The second strategy is developed based on the backward feature elimination scheme but uses fewer iterations to achieve the end result, namely, AUC-driven subtractive iteration algorithm (ASI) (Fig. 15).

Preprocessing using the ASI algorithm showed that there were at least 28% of features disrupting the prediction model. The loss of some of the individual nucleotide 4-mer feature seemed not to influence the performance of the classifier at this stage, but the similarities between IRGs and ISGs (Fig. 12), particularly in the 4-mer features, were a cause for concern when the model was used to predict new data, especially unknown IRGs.

**Figure 13:**
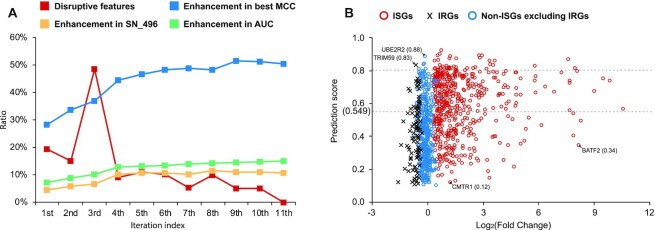
The optimisation of the machine learning model with the ASI algorithm. (A) Change of the prediction models based on the one generated with all 518 features (poorly performing feature vector = 144, best MCC = 0.250, SN_496 = 0.615, and AUC = 0.6509). (B) Distribution of prediction scores generated by the ASI-optimised model for human genes with different expression levels in the IFN-α system. The ISGs and non-ISGs shown in (B) are randomly selected through an undersampling strategy [64] on dataset S2. The list of gene names can be found in Supplementary Data S1.

When using the ASI algorithm, the number of disrupting features did not stabilise until the algorithm reached the 11th iteration. The remaining 74 features constituted our optimum feature set for predicting the ISGs (Table 4). Among them, 14 and 9 features displayed positive and negative correlations with the level of upregulation in IFN-α experiments (*P* < 0.05). During the procedure, the AUC kept increasing steadily and reached 0.7479 at the end (Table 3). The MCC also showed an overall improvement, although it fluctuated slightly during the last few iterations. By ranking the scores calculated by the prediction model, we found 68.1% of the 496 genes (equal to the number of ISGs in the training dataset) were successfully predicted as the ISGs. Fig.13B illustrates the distribution of prediction scores generated by the ASI-optimised model for human genes with different expressions in IFN-α experiments. Human genes with higher upregulation in IFN-α experiments tend to obtain higher prediction scores from our optimised machine learning model (PCC = 0.243, *P =*4.2E-10).

**Table 4:** The optimum 74 features contributing to predicting the ISGs

Evolutionary features (2)		
Number of human paralogues, average dS within human paralogues^N^		
**Codon usage features (10)**		
Codon usage: CTA (L)^P^	Codon usage: ATT (I)	Codon usage: TAT (Y)
Codon usage: GCG (A)^N^	Codon usage: CAC (H)^N^	Codon usage: TGC (C)
Codon usage: CGT (R)	Codon usage: CGA (R)	Codon usage: CGG (R)^N^
Codon usage: AGA (R)^P^		
**Genetic composition features (40)**		
DNA AC content	Dinucleotide CpT composition	DNA 4-mer CGCG composition^N^
DNA 4-mer AATC composition^P^	DNA 4-mer TCGT composition	DNA 4-mer GATG composition^P^
DNA 4-mer AACA composition	DNA 4-mer TGAG composition^P^	DNA 4-mer GACC composition
DNA 4-mer ATAT composition	DNA 4-mer TGTA composition	DNA 4-mer GACG composition
DNA 4-mer ATGT composition^P^	DNA 4-mer CACG composition	DNA 4-mer GAGT composition^P^
DNA 4-mer ACAC composition	DNA 4-mer CTCC composition	DNA 4-mer GTAC composition
DNA 4-mer ACTA composition	DNA 4-mer CCAC composition	DNA 4-mer GTGT composition
DNA 4-mer ACTC composition	DNA 4-mer CCTA composition	DNA 4-mer GTGC composition
DNA 4-mer ACCG composition	DNA 4-mer CCTC composition^P^	DNA 4-mer GTGG composition
DNA 4-mer TATG composition	DNA 4-mer CCGT composition	DNA 4-mer GCAA composition^P^
DNA 4-mer TTCT composition	DNA 4-mer CGAG composition	DNA 4-mer GCTC composition
DNA 4-mer TTCG composition	DNA 4-mer CGTG composition	DNA 4-mer GCCT composition
DNA 4-mer TTGA composition	DNA 4-mer CGCA composition	DNA 4-mer GGGG composition
DNA 4-mer TCAT composition		
**Proteomic composition features (9)**		
Arginine composition, cysteine composition^P^, methionine composition		
Basic amino acid composition (R/H/K)^P^	Sulphur amino acid composition (C&M)^P^	
Hydroxyl amino acid composition (S&T)^N^	Small amino acid composition (N/D/C/P/T)^N^	
Large amino acid composition (R/I/L/K/M)^P^		
Uncharged amino acid composition (A/N/C/Q/G/I/L/M/F/P/S/T/W/Y/V)^N^		
**Features about human interactome network (3)**		
Average shortest paths^P^, betweenness, neighbourhood connectivity^N^		
Sequence pattern features (8)		
SLNP: ATA[AG][TG]	SLNP: TAT[AT]T	SLNP: T[AT]AAA
SLNP: [ATG]TGTA	SLAAP: SxNxE	SLAAP: ENE
SLAAP: SVI	Co-occurrence of SLAAPs (count)	

P Features are positively associated with the level of upregulation in IFN-α experiments (*P* < 0.05).

N Features are negatively associated with the level of upregulation in IFN-α experiments (*P* < 0.05).

However, there were also some ISGs incorrectly predicted by our model, even though they were highly upregulated, for example, basic leucine zipper ATF-like transcription factor 2 (BATF2, prediction score = 0.34). The model produced 33 ISGs with a prediction score higher than 0.8, but this number for the non-ISGs reduced to 6, including 1 IRG (tripartite motif containing 59 [TRIM59]). The highest prediction score within the non-ISGs was found on ubiquitin conjugating enzyme E2 R2 (UBE2R2, prediction score = 0.88). It contains many features similar to the ISGs but was not differentially expressed in the presence of IFN-α in the human fibroblast cells [3]. The lowest prediction score within ISGs was found on cap methyltransferase 1 (CMTR1, prediction score = 0.12) due to the weak signal from its features. For instance, CMTR1 protein does not contain any ISG-favoured SLAAPs listed in Table 1. The influence of the IRGs on the prediction was reflected in the training dataset but was not significant. Compared with human genes not differentially expressed in the IFN-α experiments (non-ISGs but not IRGs), there were slightly more IRGs unsuccessfully classified when using a threshold of 0.549 (Pearson's chi-squared tests: *M*_1_ = 27%, *M*_2_ = 24%, *P* > 0.05).

### Review of different testing datasets

**Figure 14:**
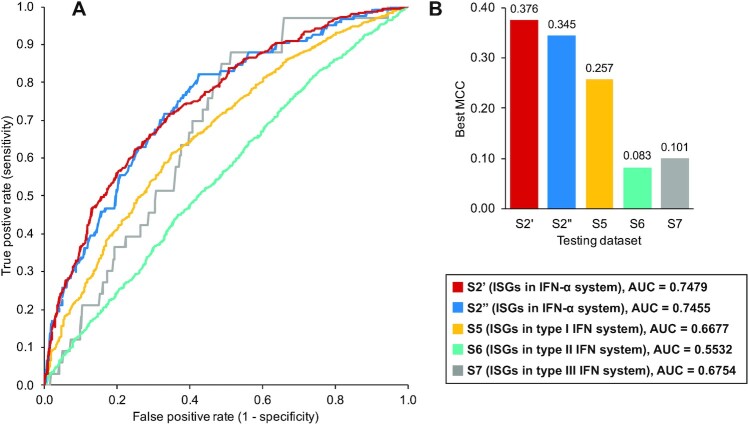
The performance of our optimised model on different datasets. (A, B) The AUC and best MCC. S2′ is the training dataset used in this study. It randomly includes 496 ISGs and an equal number of non-ISGs from dataset S2 that contains ISGs/non-ISGs with high confidence (Table 5). Evaluation on this dataset in (A) is processed via 5-fold cross-validation. S2′′ is the testing dataset constructed with the remaining human genes in dataset S2. S5, S6, and S7 are collected from the Interferome database [24], including human genes with different responses to the type I, II, and III IFNs, respectively. The label and usage of these human genes are provided in Supplementary Data S1.

In this study, we trained and optimised a SVM model from our training dataset S2′ and prepared 7 testing datasets (dataset S2′′/S3/S4/S5/S6/S7/S8) to assess the generalisation capability of our model under different conditions (Table 5). The S2′′ testing dataset was a subset of dataset S2. The prediction performance on this testing dataset was close to that in the training stage with an AUC of 0.7455 (Fig. 14A). The best MCC value (0.345) was achieved when setting the judgement threshold to 0.438, which meant that the prediction model was sensitive to signals related to ISGs. In this case, it performed predictions with high sensitivity but inevitably produced many false positives, especially within IRGs.

**Table 5:** A breakdown of datasets used in this study

Dataset	Brief description	IFN system	ISGs	Non-ISGs	ELGs	Usage
S1	Background human genes	IFN-α in fibroblast cells	1,315	7,304	2,217	Analyses
S2	Dataset with high confidence	IFN-α in fibroblast cells	620	874	0	Analyses
S2′	Training subset of S2	IFN-α in fibroblast cells	496	496	0	Training
S2′′	Testing subset of S2	IFN-α in fibroblast cells	124	378	0	Testing
S3	ISGs with low confidence in S1	IFN-α in fibroblast cells	695	0	0	Testing
S4	IRGs divided from S1	IFN-α in fibroblast cells	0	1,006	0	Analyses/testing
S5	ISGs from Interferome [24]	Type I IFNs in all cells	1,259	872	0	Testing
S6	ISGs from Interferome [24]	Type II IFN in all cells	2,229	755	0	Testing
S7	ISGs from Interferome [24]	Type III IFN in all cells	33	1,683	0	Testing
S8	ELGs divided from S1	IFN-α in fibroblast cells	0	0	2,217	Testing

In the S3 testing dataset, we used 695 ISGs with low confidence. The overall accuracy (equals to sensitivity as there were no negatives) only reached 44.0% when using a judgement threshold of 0.549, about 0.18 lower than SN under the same threshold in the training dataset S2′ (Table 3). It is expected as some of their inherent attributes make them slightly upregulated, silent, or even repressed (e.g., become non-ISGs in other IFN systems) in response to some IFN-triggered signalling. On this testing dataset, our machine learning model produced 38 (5.5%) ISGs with a prediction score higher than 0.8. This number was also lower than that on the training dataset S2′. It further indicates the relatively low confidence for the ISGs included in dataset S3.

The S4 testing dataset was constructed to illustrate our hypothesis that there are some patterns shared among the ISGs and IRGs at least in the IFN-α system in the human fibroblast cells. On this testing dataset, the prediction accuracy (equals to SP as there were no positives) was 60.2% under the judgement threshold of 0.549, about 0.15 lower than the SP under the same threshold in the training dataset S2′ (Table 3). Leucine rich repeat containing 2 (LRRC2), carbohydrate sulfotransferase 10 (CHST10), and eukaryotic translation elongation factor 1 epsilon 1 (EEF1E1) showed strong signals of being ISGs (probability score >0.9). In total, there were 56 (5.6%) IRGs being incorrectly predicted as ISGs with prediction scores higher than 0.8. This high score was found in an estimated 8.1% of the ISGs but was observed in only 1.2% of human genes not differentially expressed in the IFN-α experiments (Fig. 13B). These results indicate that there are a considerable number of IRGs incorrectly predicted as ISGs in the S4 testing dataset due to their close distance to the ISGs in the high-dimensional feature space. This may be the case for many other datasets, including dataset S2′′, S5, S6, S7, and S8. It also supports our hypothesis about the shared patterns from the machine learning aspect and is consistent with the results shown in Fig. 12.

The next 3 testing datasets (S5, S6, and S7) were collected from the Interferome database [24] to test the applicability of the machine learning model across different IFN types. The ISGs in these testing datasets were all highly upregulated (log_2_(fold change) >1.0) in the corresponding IFN systems while all the non-ISGs were not upregulated after corresponding IFN treatments (log_2_(fold change) <0). The results shown in Fig. 14 reveal that the ISGs triggered by type I or III IFN signalling can still be predicted by our machine learning model, but the performance is limited to some extent (AUC = 0.6677 and 0.6754, respectively). However, it is almost impossible to make normal predictions with the current feature space for human genes upregulated by type II IFNs (AUC = 0.5532).

**Figure 15:**
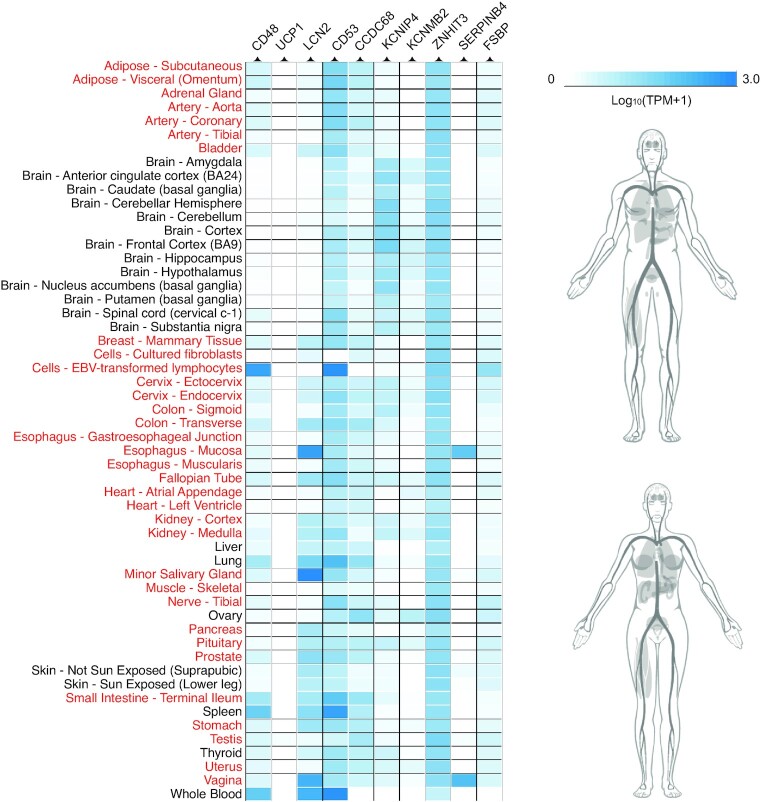
Expression of the ELGs in different tissues. Expression data for 10 ELGs are collected from the Genotype-Tissue Expression project (https://gtexportal.org/) [65]. The tissues in red are not included in the Interferome database [24]. White boxes in the heatmap indicate that there are no data available for genes in the corresponding tissues. The overall expression level of these 10 ELGs is reflected via human perspective photo retrieved from Expression Atlas (https://www.ebi.ac.uk/gxa) [66].

The S8 testing dataset consisted of 2,217 human genes that were insufficiently expressed in IFN-α experiments in the human fibroblast cells [3]. The results showed that there were around 41.2% ELGs being predicted as the ISGs when using a judgement threshold of 0.549. This was approximately 0.21 lower than the SN under the same threshold in the training dataset S2′ (Table 3). It suggests that there are more non-ISGs than ISGs in this dataset, which is consistent with the results shown in Fig. 12. Particularly, we found 10 ELGs with prediction scores higher than 0.9: CD48 molecule, CD53 molecule, lipocalin 2 (LCN2), uncoupling protein 1 (UCP1), coiled-coil domain containing 68 (CCDC68), potassium calcium–activated channel subfamily M regulatory beta subunit 2 (KCNMB2), potassium voltage–gated channel interacting protein 4 (KCNIP4), zinc finger HIT-type containing 3 (ZNHIT3), serpin family B member 4 (SERPINB4), and fibrinogen silencer binding protein (FSBP). By retrieving data from the Genotype-Tissue Expression project [65], we found that the expression of these ELGs was generally limited with the exception of CD53 and ZNHIT3 (Fig. 15). The expression data of CD53 were not included in the OCISG database [3] and also limited in the Interferome database [24]. It only showed slight upregulation after type I IFN treatments in blood, liver, and brain, but there is currently no record of its expression level in the presence of IFN-α in the human fibroblast cells. ZNHIT3 is another well-expressed gene lacking information in the OCISG. In the Interferome database [24], we found that ZNHIT3 could be upregulated after IFN treatments in some fibroblast cells on the skin. As for the remaining 8 ELGs, despite their limited expression in the human fibroblast cells, their features suggest that they are very likely to be IFN-α stimulated in a currently untested cell type.

## Discussion

In this study, we investigated the characteristics that influence the expression of human genes in IFN-α experiments. We compared the ISGs and non-ISGs through multiple procedures to guarantee strong signals for the ISGs and to avoid cell-specific influences that resulted in the lack of ISG expression in certain cell types [2]. Even some highly upregulated ISGs can become downregulated when the biological conditions change, exemplified by the performance of C-X-C motif chemokine ligand 10 (CXCL10) on liver biopsy specimens after IFN-α treatment. This refinement is necessary as the representation of features between the ISGs and background human genes shows that many non-ISGs, especially IRGs, have similar feature patterns to the ISGs (Fig. 12).

Generally, the ISGs are less evolutionarily conserved and include more human paralogues than the non-ISGs. They have specific nucleotide patterns exemplified by the depletion of GC content and have a unique codon usage preference in coding proteins. There are a number of SLNPs widely observed in the cDNA of the ISGs, which are relatively rare in the non-ISGs (Supplementary Data S4). Likewise, there are also many SLAAPs highlighted in the sequences of ISG products that are absent or rare in the non-ISG products (Table 1). In the human PPI network, the ISG products tend to have higher betweenness than the background human protein. Abnormal expression or knockout of these proteins will increase the diameter of the network and may lead to some lethal consequences that are not tolerated in signalling pathways [67–69]. These ISG-specific patterns may be the result of the evolution of the innate immune system in vertebrates and could be adaptations to the cellular environment induced by interferon following a pathogenic infection [70]. It is also possible that some of the particular SLNPs and SLAAPs may be functionally important as the cell changes from noninfected to infected. Experimental evidence will be necessary to investigate this.

We found that dN/dS ratio was positively correlated with gene upregulation following IFN-α treatments (Fig. 10). This suggests ISGs are on average under stronger adaptive evolutionary selection pressure than the non-ISGs possibly linked to their evolution as antiviral molecules. Some other properties of the ISGs facilitate or elevate their expression after IFN-α treatments but may also be used by viruses to escape from IFN-α–mediated antiviral response [ 22]. For instance, we found arginine was underrepresented in the ISG products compared to the non-ISG products. As arginine is essential for the normal proliferation and maturation of human T cells [ 71], such depletion in the ISG products may leave a risk of inhibiting T‐cell function and potentially increase susceptibility to infections [72]. Furthermore, the special pattern of the ISGs also promotes the representation of some features even if they are not well represented in nature, for example, the higher cysteine composition in the ISGs. We hypothesize that it may be helpful to activate T cells to regulate protein synthesis, proliferation, and secretion of immunoregulatory cytokines [73, 74]. There are also some features (e.g., methionine composition) not differentially represented between the ISGs and non-ISGs but that play important roles in IFN-α–mediated immune responses. For example, there is evidence for the methionine content playing a role in the biosynthesis of S-adenosylmethionine (SAM), which can improve interferon signalling in cell culture [ 75, 76].

As previously mentioned, there were similar patterns between the feature representation of the ISGs and IRGs, which led to an unclear boundary for the ISGs and non-ISGs in the feature space. We found significant differences in the representation of features on evolutionary conservation (Fig. 4) between the ISGs and non-ISGs, but they became nonsignificant when comparing the ISGs with IRGs. Similar phenomena were observed on many features deciphered from the canonical transcript (e.g., dinucleotide composition and codon usage features). We hypothesise that IRGs are former ISGs that have evolved to be downregulated to avoid any unintended harmful consequences. Furthermore, despite so many similarities between the ISGs and IRGs, the separate classification of these genes is still possible. The 4-mer composition features can be considered the key features as most of them are differentially represented between ISGs and IRGs (Fig. 12). Using proteomic features can also help to differentiate the ISGs from IRGs but is not as predictive as using 4-mer features.

In the machine learning framework, we developed the ASI algorithm to remove poorly performing features but kept features that do not influence prediction performance when removed individually from iterations. Features may have synergistic effects on the prediction performance. The elimination of some specific features may ruin such improvement even when they were individually uninformative for the improvement of the classifier. In this case, keeping as many useful features as possible seems to be a reasonable option but will greatly increase the dimension of the feature space and increase the risk of overfitting [77]. By contrast, our ASI algorithm avoided such a risk and kept the synergistic effect of different features through iterations.

In the prediction task, we found some previously labelled non-ISGs with very high prediction scores, suggesting that they had some inherent properties consistent with them being stimulated after IFN-α treatments. Some (e.g., UBE2R2) have been shown to be significantly upregulated after IFN-α treatment [78]. The non-ISG label had been assigned because the relevant expression data in the presence of IFN-α were not included in the OCISG [3] and Interferome databases [24]. We also found 10 ELGs with very high prediction scores (>0.9). Literature searches on these genes indicate that they are likely to be involved in the innate immune response [79, 80]. Their responses may be limited to certain tissues or cell types for which there are limited expression data in the Interferome database [24]. For example, LCN2 has been shown to mediate an innate immune response to bacterial infections by sequestering iron [79] and is induced in the central nervous system of mice infected with West Nile virus encephalitis [81]. CD48 was shown to increase in levels in the context of human IFN-α/β/γ stimulation [80]. Interestingly, CD48 is also the target of immune evasion by viruses [82] and has been captured in the genome of cytomegalovirus and undergone duplication [83]. Evidence for other ELGs is harder to assess, particularly those for which expression is absent in a range of tissues (e.g., UCP1 in Fig. 15). UCP1 is a mitochondrial carrier protein expressed in brown adipose tissue (BAT) responsible for nonshivering thermogenesis [84]. It is possible that UCP1 is stimulated directly or indirectly by IFN-α in BAT, resulting in the defended elevation of body temperature in response to infection.

We developed the machine learning model based on experimental data from the human fibroblast cells stimulated by IFN-α. It can be generalised to type I or III IFN systems, presumably because activations of type I and III ISGs are both controlled by ISRE [9] and aim to regulate host immune response [10–12]. However, our model cannot be used for predictions in the type II IFN system (AUC = 0.5532, best MCC = 0.083, Fig. 14). This is possibly caused by the different control elements used and their different roles in human immune activities [14]. One feasible strategy is to reclassify the ISGs/non-ISGs based on the IFN experiments in the type II IFN system. Using only the overlapping ISGs and non-ISGs in both type I and type II IFN system for modelling could be another solution. In summary, our analyses highlight some key sequence-based features that are helpful to distinguish the ISGs from non-ISGs, or IRGs. While reliable ISG prediction remains a difficult challenge, our machine learning model is able to produce a list of putative ISGs to support IFN-related research. As knowledge of the ISG functions continues to be elucidated by experimentalists, the *in silico* approach applied here can in future be extended to classify the different functions of ISGs. The “important” features mentioned in this study may become a focus for investigating the interferon antagonists expressed by different viruses [85].

## Methods

### Dataset curation

In this study, we retrieved 2,054 ISGs (upregulated), 12,379 non-ISGs (downregulated or not differentially expressed), and 3,944 unlabelled human genes (ELGs with less than 1 count per million reads mapping across the 3 biological replicates [86, 87]) from the OCISG database [3]. Gene clusters in the OCISG database were built through Ensembl Compara [88], which provided a thorough account of gene orthology based on whole genomes available in Ensembl [58]. Labels of these human genes were defined based on the fold change and a false discovery rate (FDR) following the IFN-α treatments in the human fibroblast cells. We searched the collected 18,377 entries against the RefSeq database [32] to decipher features based on appropriate transcripts (canonical) [89] coding for the main functional isoforms of these human genes. It produced 1,315, 7,304, and 2,217 results for the ISGs, non-ISGs, and ELGs, respectively. These 10,836 human genes were well annotated by multiple online databases and were used as the background dataset S1 in the analyses.

For the purpose of generating a set of human genes with high confidence of being upregulated and not upregulated in response to the IFN-α, we searched the recompiled 8,619 human genes (ISGs or non-ISGs) against Interferome [24]. We filtered out the ISGs without high upregulation (log_2_(fold change) >1.0) or with obvious downregulation (log_2_(fold change) <−1.0) in the presence of type I IFNs. This procedure guaranteed a refined ISG dataset with strong levels of stimulation induced by any type I IFNs and reduced biases driven by the IRGs for the analyses and predictions. We filtered out the non-ISGs showing enhanced expression after type I IFN treatments (log_2_(fold change) >0). The exclusion of these non-ISGs could effectively reduce the risk of involving false negatives in analyses and producing false positives in predictions. As a result, the refined dataset S2 contains 620 ISGs and 874 non-ISGs with relatively high confidence.

The training procedure in the machine learning framework was conducted on the balanced dataset S2′. It consisted of 992 randomly selected ISGs and non-ISGs from dataset S2. The remaining human genes in S2 were used for independent testing. Additionally, we also constructed another 6esting datasets for the purpose of review and assessment. Dataset S3 contained 695 ISGs with low confidence compared to those ISGs in dataset S2. Some of them could be non-ISGs or even IRGs in the type I IFN system. Dataset S4 contained 100,6 IRGs from the human fibroblast cell experiments. Dataset Ss5, S6, and S7 were constructed based on records for experiments in type I, II, and III IFN systems from Interferome (RRID:SCR_007743) [24]. The criterion for an ISG in the latter 3 datasets was a high level of upregulation (log_2_(fold change) >1.0) while that for non-ISGs was no upregulation after IFN treatments (log_2_(fold change) <0). The last testing dataset S8 was derived from our background dataset S1, containing 2,217 ELGs. A breakdown of the aforementioned 8 datasets is shown in Table 5. Detailed information of the human genes used in this study is provided in Supplementary Data S1. The cDNA and protein sequences are accessible at [90].

### Generation of discrete features

We encoded 397 discrete features from aspects of evolution, nucleotide composition, transcription, amino acid composition, and network properties. Original values of these features for our compiled 10,836 human genes are accessible at [90].

From the perspective of evolution, we used the number of transcripts, ORFs, and count of exons used for coding to quantify the alternative splicing process. Genes with more transcripts and ORFs have higher alternative splicing diversity to produce proteins with similar or different biological functions [33, 91, 92]. Frequent use of protein-coding exons indicates more complex alternative splicing products [93]. Here, duplication and mutation features were measured by the number of within-species paralogues and substitutions [34, 35]. These data were collected from BioMart (RRID:SCR_002987) [58] to assess the selection on protein sequences and mutational processes affecting the human genome [94].

From the perspective of nucleotide composition, we calculated the percentage of adenine, thymine, cytosine, guanine, and their 4-category combinations in the coding region of the canonical transcript. The first category measured the proportion of 2 different nitrogenous bases out of the implied 4 bases (e.g., GC content). The second category also focused on the combination of 2 nucleotides but added the impact of phosphodiester bonds along the 5′ to 3′ direction (e.g., CpG content) [95]. The third category calculated the occurrence frequency of 4-mers (e.g., “CGCG” composition to involve some positional resolution) [41]. The last category considered the co-occurrence of SLNPs. From the perspective of transcription, we calculated the usage of 61 coding codons and 3 stop codons in the coding region of the canonical transcripts. Codon usage biases are observed when there are multiple codons available for coding 1 specific amino acid. They can affect the dynamics of translation and thus regulate the efficiency of translation and even the folding of the proteins [40, 96].

From the perspective of amino acid composition, we calculated the percentage of 20 standard amino acids and their combinations based on their physicochemical properties [46]. Patterns in the amino acid level are considered to have a direct impact on the establishment of biological functions or to reflect the result of strong purifying selection [47]. Based on the chemical properties of the side chain, we grouped amino acids into 7 classes, including aliphatic, aromatic, sulphur, hydroxyl, acidic, amide, and basic amino acids. We also grouped amino acids based on geometric volume, hydropathy, charge status, and polarity but found some overlaps among these features. For instance, amino acids with basic side chains are all positively charged. Aromatic amino acids all have large geometric volumes (volume >180 cubic angstroms). Likewise, we also considered the co-occurrence of short linear sequence patterns at the protein level. These co-occurring SLAAPs may relate to potential mechanisms regulating the expression of the ISGs [97].

To infer network properties for the gene products, we constructed a human PPI network based on 332,698 experimentally verified interactions (confidence score >0.63) from HIPPIE (RRID:SCR_014651) [56]. Nodes and edges of this network are provided at our web server. Eight network-based features, including the average shortest path, closeness, betweenness, stress, degree, neighbourhood connectivity, clustering coefficient, and topological coefficient, were calculated from this network. Isolated nodes or proteins were not included in our network and were assigned zero values for all these 8 features. The shortest path measures the average length of the shortest path between a focused node and others in the network. Closeness of a node is defined as the reciprocal of the length of the average shortest path. Proteins with a low value of the shortest paths or closeness are close to the centre of the network. Betweenness reflects the degree of control that 1 node exerted over the interactions of other nodes in the network [98]. Stress of a node measures the number of shortest paths passing through it. Proteins with a high value of betweenness or stress are close to the bottleneck of the network. Degree of a node counts the number of edges linked to it while neighbourhood connectivity reflects the average degree of its neighbours. Proteins with high values of degree or neighbourhood connectivity are close to the hub of the network. They are considered to play an important role in the establishment of the stable structure of the human interactome [99]. Clustering and topological coefficient measure the possibility of a node to form clusters or topological structures with shared neighbours. The former coefficient can be used to identify the modular organisation of metabolic networks [100] while the latter one may be helpful to find out virus mimicry targets [53].

### Generation of categorical features

In this study, categorical features were used to check the occurrence of short linear sequence patterns in the genome and proteome. SLNPs constructed in this study contained 3 to 5 random nucleotides, producing 708,540 alternative choices. SLNPs with no restrictions on their first or last position were not taken into consideration as their patterns could be expressed in a more concise way. A SLNP was picked out to encode a binary feature when its occurrence level in the coding region of the canonical ISG transcripts was significantly higher than that for the non-ISGs (Pearson's chi-squared test: *P* < 0.05). SLAAPs were constructed with 3 to 4 fixed amino acids separated by putative gaps. The gap could be occupied by at most 1 random amino acid, producing 1,312,000 alternative choices. Likewise, binary features were prepared for SLAAPs showing significant enrichment in the ISG products than in the non-ISG products (Pearson's chi-squared test: *P* < 0.05). Since there were lots of results rejecting the null hypothesis, we adopted the Benjamini–Hochberg correction procedure to avoid type I error [43]. Additionally, we also encoded 2 features to check the co-occurrence or absence of multiple SLNPs and SLAAPs. This co-occurrence status might be a better representation of functional sites composed of short stretches of adjacent nucleobases or amino acids surrounding SLNPs or SLAAPs [47].

### Assessment of associations between feature representation and IFN-triggered stimulations

We obtained 8,619 human genes with expression data from the OCISG database [3]. In total, 4,111 of them were annotated with a positive log_2_(fold change) ranging from 0 to 12.6, which meant they were upregulated after IFN-α treatments in the human fibroblast cells. In order to measure the average level of feature representation (AREP) for genes with similar expression during IFN stimulations, we introduced a 0.1-length sliding window to divide the data into 126 bins with different log_2_(fold change). Here, PCC was introduced to test the association between the representation of discrete features and IFN-α–triggered stimulation (log_2_(fold change) >0). It can be formulated as

(1) \begin{equation*}
PCC\left( f \right)\,\, = \frac{1}{{n - 1}}\Sigma _{i = 1}^n\left( {\frac{{LF{C_i} - {M_0}}}{{S{D_0}}}} \right) \times \left( {\frac{{ARE{P_i} - {M_f}}}{{S{D_f}}}} \right) \end{equation*}

where *n* is the number of divided parts that equals 126 in this study; $LF{C_i}$ and $ARE{P_i}$ are the value of log_2_(fold change) and AREP in the *i*th part; ${M_0}$ and $S{D_0}$ are the mean and standard deviation of log_2_(fold change), which are set as 6.4 and 3.7, respectively, in this study; and ${M_f}$ and $S{D_f}$ are the mean and standard deviation of 126 AREPs that reflect the representation of the considered feature. To make fair comparisons among features with different scales, we normalised them based on the major value of their representations:

(2) \begin{equation*}
Norm\,\,\left( f \right) = \left\{ {\begin{array}{@{}*{1}{c}@{}} {\,\,1,\,\,f > UB\left( f \right)}\\ {\frac{{f - LB\left( f \right)}}{{UB\left( f \right) - LB\left( f \right)}}}\\ {\,\,0,\,\,f < LB\left( f \right)} \end{array}} \right.\,\,,\,\,LB\left( f \right) < f < UB\left( f \right) \end{equation*}

where $LB( f )$ and $UB( f )$ are the lower and upper bound representing the 5th and 95th percentile within representation values for the target feature. The representation of feature was considered to have a stronger positive/negative association with IFN-α–triggered stimulations if the PCC calculated from the normalised features was closer to 1.0/−1.0 and the *P*-value calculated by the Student *t*-test was lower than 0.05.

### Machine learning and optimisation

**Figure 16:**
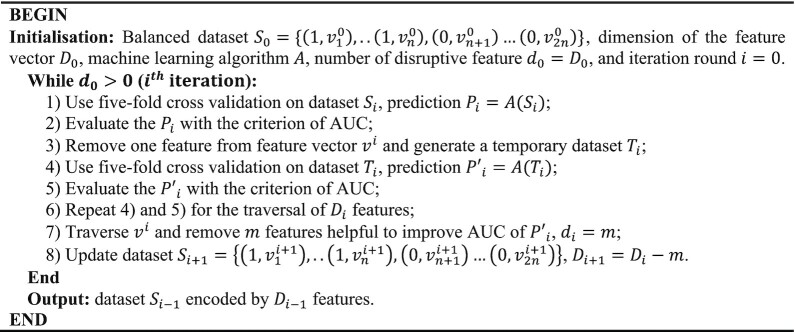
The pseudo-code of the AUC-driven subtractive iteration algorithm.

We designed a machine learning framework for the prediction of ISGs. First, all features were encoded and normalised based on their major representations (Eq. 2). Then we used an undersampling procedure [64] to generate a balanced dataset from dataset S2 for training and modelling. The SVM with radial basis function [61] was used as the basic classifier. It maps the normalised feature space to a higher dimension to generate a space plane to better classify the majority of positive and negative samples. In order to avoid overfitting [77] and make it easier for the SVM model to generate an appropriate classification plane that involved fewer false positives and false negatives, here we propose a subtractive iteration algorithm driven by the change of AUC. This algorithm is developed based on the traditional backward feature elimination method [63] but uses fewer iterations to filter out poorly performing features (Fig. 16). In each iteration, we traversed the features and removed those that did not improve the AUC of the prediction results. In the testing procedure, we encoded the optimum features for testing samples and placed them in the optimised feature space. Samples with longer distance to the optimised classification plane indicated a stronger signal of being the ISGs or non-ISGs. They were more likely to get higher prediction scores (close to 0 or 1) from the SVM model.

### Performance evaluation

In this study, the prediction results were evaluated with 3 threshold-dependent criteria, including sensitivity, specificity, and MCC [101], and 2 threshold-independent criteria: SN_n and AUC. Sensitivity and specificity were used to assess the quality of the machine learning model in recognising ISGs and non-ISGs, respectively, while MCC provided a comprehensive evaluation for both positives and negatives. The number of “n” in the SN_n criterion was determined based on the number of ISGs used for testing. It was used to measure the upper limit of the prediction model as well as to check the existence of important false positives close to the class of ISGs from the perspective of data expression. Finally, AUC was a widely used criterion to evaluate the prediction ability of a binary classifier system. The group of interest was almost unpredictable in a specific binary classifier system if the AUC of the classifier was close to 0.5.

### Availability of Source Code and Requirements

- Project name: ISGPRE
- Project homepage: http://isgpre.cvr.gla.ac.uk/
- Operating system: Platform independent
- Programming language: Java
- Other requirements: Docker or JDK 8+
- Docker image: https://hub.docker.com/repository/docker/hchai01/isgpre
- Biotools repository: https://bio.tools/isgpre
- Research Resource Identification Initiative ID: SCR_022730
- Documentation and tutorials: https://github.com/HChai01/ISGPRE
- License: GNU GPL v3.0

## Data Availability

The implemented web server and all reproduceable data are freely accessible at https://isgpre.cvr.gla.ac.uk/ and [90]. Code snapshots and other supplementary data are also available in the *GigaScience* GigaDB repository [102].

## Additional Files

**Supplementary Data S1**. Basic information and usage of our compiled 10,836 human genes.

**Supplementary Data S2**. The result of Mann–Whitney *U* tests for discrete features.

**Supplementary Data S3**. Association between feature representations and IFN-α stimulations.

**Supplementary Data S4**. The result of Pearson's chi-squared tests for sequence motifs.

**Supplementary Data S5**. Features and their individual performance in machine learning.

giac103_GIGA-D-22-00042_Original_Submission

giac103_GIGA-D-22-00042_Revision_1

giac103_GIGA-D-22-00042_Revision_2

giac103_Response_to_Reviewer_Comments_Original_Submission

giac103_Response_to_Reviewer_Comments_Revision_1

giac103_Reviewer_1_Report_Original_Submission Milton Pividori, Ph.D. -- 3/29/2022 Reviewed

giac103_Reviewer_1_Report_Revision_1 Milton Pividori, Ph.D. -- 9/10/2022 Reviewed

giac103_Reviewer_2_Report_Original_Submission Muthukumaran Venkatachalapathy -- 6/1/2022 Reviewed

giac103_Supplemental_Files

## Abbreviations

APC: anaphase promoting complex; AREP: average level of feature representation; ASI: AUC-driven subtractive iteration algorithm; AUC: area under the receiver operating characteristic curve; cDNA: complementary DNA; dN: nonsynonymous substitutions; dS: synonymous substitutions; ELGs: human genes with limited expression in the IFN-α experiments; FDR: false discovery rate; FFS: forward feature selection; GAF: IFN-γ activation factor; GAS: gamma-activated sequence promoter elements; gBGC: GC-biased gene conversion; HIPPIE: Human Integrated Protein–Protein Interaction rEference; IDRs: intrinsically disordered regions; IFNAR: interferon-α receptor; IFNGR: IFN-γ receptor; IFNLR1: IFN-λ receptor 1; IFNs: interferons; IL-10R2: interleukin-10 receptor 2; IRF9: interferon regulatory factor 9; IRG: interferon repressed (downregulated) human genes; ISGF3: interferon stimulated gene factor 3 complex; ISGs: interferon-stimulated (upregulated) human genes; ISRE: interferon-stimulated response elements; JAK1: Janus kinase 1; KNN: k-nearest neighbours; MCC: Matthews correlation coefficient; non-ISGs, human genes not significantly upregulated by interferons; OCISG: Orthologous Clusters of Interferon-Stimulated Genes; ORF: open reading frame; PCC: Pearson's correlation coefficient; PPI: protein–protein interaction; RefSeq: Reference Sequence; RF: random forest; SLAAP: short linear amino acid pattern; SLNP: short linear nucleotide pattern; SN_496: sensitivity of samples with the top 496 prediction scores; STAT: signal transducer and activator of transcription; SVM: support vector machine.

## Conflict of Interest

The authors have declared no conflict of interest.

## Funding

H.C. is supported by the China Scholarship Council (201706620069). J.H., Q.G., and D.L.R. are supported by the Medical Research Council (MC_UU_1201412). The funders had no role in study design, data collection and analysis, decision to publish, or preparation of the manuscript.

## Authors’ Contributions

Conceptualization: all authors; data curation: H.C.; formal analysis: H.C.; funding acquisition: D.L.R.; investigation: H.C.; methodology: H.C.; project administration: D.L.R., J.H.; resources: Q.G., J.H., D.L.R.; web server: H.C.; software: H.C.; supervision: Q.G., J.H., D.L.R.; validation: all authors; visualization: H.C.; writing original draft: H.C.; writing review and editing: all authors.
