# Supplementary material for: Defining the characteristics of interferon-alpha–stimulated human genes: insight from expression data and machine learning
Source: Gigascience. 2022 Nov 18;11:giac103. doi: 10.1093/gigascience/giac103 (PMC9673497; doi:10.1093/gigascience/giac103)
Supplement: giac103_GIGA-D-22-00042_Original_Submission [file giac103_giga-d-22-00042_original_submission.pdf]

GigaScience

Defining the characteristics of interferon-alpha-stimulated human genes: insight from expression data and machine-learning

--Manuscript Draft--

**Manuscript Number:**	GIGA-D-22-00042
**Full Title:**	Defining the characteristics of interferon-alpha-stimulated human genes: insight from expression data and machine-learning
**Article Type:**	Research
**Funding Information:**	Medical Research Council (MC_UU_1201412) — Prof David L. Robertson<br>China Scholarship Council (201706620069) — Mr Haiting Chai
**Abstract:**	Background<br><br>A virus-infected cell triggers a signalling cascade resulting in the secretion of interferons (IFNs). It in turn induces the up-regulation of the IFN stimulated genes (ISGs) that play anti-pathogen roles in host defenses. Here, we conducted analyses on large-scale data relating to evolution, gene expression, sequence compositions, and network properties to elucidate factors associated with the stimulation of human genes in response to the typical IFN-α.<br><br>Results<br><br>We propose that the ISGs are less evolutionary conserved than genes that are not significantly stimulated in IFN experiments (non-ISGs). ISGs show obvious depletion of GC-content in the coding region, leading to differential representations in their sequence compositions. The IFN repressed human genes (IRGs), which are down-regulated in IFN experiments can have similar properties to the ISGs. Additionally, we also design a machine-learning framework integrating the support vector machine and novel feature selection algorithm. It achieves an area under the receiver operating characteristic curve (AUC) of 0.7455 for the ISG prediction and demonstrates the similarity between the ISGs triggered by type I and III IFNs.<br><br>Conclusions<br><br>The ISGs have unique properties that make them different from the non-ISGs. Some of them have strong correlations with genes' expression following IFN-α stimulations. which can be used as good features in machine learning. Our model predicts several genes as potential ISGs that so far have shown no significant differential expression when stimulated with IFN-α in the cell/tissue types in the available databases. A webserver implementing our method is accessible at http://isgpre.cvr.gla.ac.uk/.
**Corresponding Author:**	Joseph Hughes<br>University of Glasgow Centre for Virus Research<br>Glasgow, Glasgow UNITED KINGDOM
**Corresponding Author Secondary Information:**	
**Corresponding Author's Institution:**	University of Glasgow Centre for Virus Research
**Corresponding Author's Secondary Institution:**	
**First Author:**	Haiting Chai
**First Author Secondary Information:**	
**Order of Authors:**	Haiting Chai<br>Quan Gu

Powered by Editorial Manager® and ProduXion Manager® from Aries Systems Corporation

	Joseph Hughes
	David L. Robertson
**Order of Authors Secondary Information:**	
**Additional Information:**	
**Question**	**Response**
Are you submitting this manuscript to a special series or article collection?	No
**Experimental design and statistics**<br><br>Full details of the experimental design and statistical methods used should be given in the Methods section, as detailed in our Minimum Standards Reporting Checklist. Information essential to interpreting the data presented should be made available in the figure legends.<br><br>Have you included all the information requested in your manuscript?	Yes
**Resources**<br><br>A description of all resources used, including antibodies, cell lines, animals and software tools, with enough information to allow them to be uniquely identified, should be included in the Methods section. Authors are strongly encouraged to cite Research Resource Identifiers (RRIDs) for antibodies, model organisms and tools, where possible.<br><br>Have you included the information requested as detailed in our Minimum Standards Reporting Checklist?	Yes
**Availability of data and materials**<br><br>All datasets and code on which the conclusions of the paper rely must be either included in your submission or deposited in publicly available repositories (where available and ethically appropriate), referencing such data using	Yes

Powered by Editorial Manager® and ProduXion Manager® from Aries Systems Corporation

a unique identifier in the references and in the “Availability of Data and Materials” section of your manuscript.<br><br>Have you have met the above requirement as detailed in our Minimum Standards Reporting Checklist?	

Powered by Editorial Manager® and ProduXion Manager® from Aries Systems Corporation

Manuscript

Click here to access/download;Manuscript;Chai-et-al-manuscript-GigaScience.docx

Defining the characteristics of interferon-alpha-stimulated human genes: insight from expression data and machine-learning

Haiting Chai[1], Quan Gu[1], Joseph Hughes[1,*], David L. Robertson[1,*]

[1]MRC-University of Glasgow Centre for Virus Research, Glasgow, United Kingdom

*david.l.robertson@glasgow.ac.uk, joseph.hughes@glasgow.ac.uk

Abstract

Background: A virus-infected cell triggers a signalling cascade resulting in the secretion of interferons (IFNs). It in turn induces the up-regulation of the IFN stimulated genes (ISGs) that play anti-pathogen roles in host defenses. Here, we conducted analyses on large-scale data relating to evolution, gene expression, sequence compositions, and network properties to elucidate factors associated with the stimulation of human genes in response to the typical IFN-α.

Results: We propose that the ISGs are less evolutionary conserved than genes that are not significantly stimulated in IFN experiments (non-ISGs). ISGs show obvious depletion of GC-content in the coding region, leading to differential representations in their sequence compositions. The IFN repressed human genes (IRGs), which are down-regulated in IFN experiments can have similar properties to the ISGs. Additionally, we also design a machine-learning framework integrating the support vector machine and novel feature selection algorithm. It achieves an area under the receiver operating characteristic curve (AUC) of

0.7455 for the ISG prediction and demonstrates the similarity between the ISGs triggered by type I and III IFNs.

Conclusions: The ISGs have unique properties that make them different from the non-ISGs. Some of them have strong correlations with genes' expression following IFN-α stimulations. which can be used as good features in machine learning. Our model predicts several genes as potential ISGs that so far have shown no significant differential expression when stimulated with IFN-α in the cell/tissue types in the available databases. A webserver implementing our method is accessible at http://isgpre.cvr.gla.ac.uk/.

Key words: interferon, interferon stimulated genes, omics data analyses, machine-learning.

Introduction

Interferons (IFNs) are a family of cytokines originally defined for their capacity to interfere with viral replication. They are secreted from host cells after an infection by pathogens such as bacteria or viruses to trigger the innate immune response with the aim of inhibiting viral spread by 'warning' uninfected cells [1]. The response induced by IFNs is usually fast and feedforward, especially to synthesize new IFNs, which guarantees a full response even if the initial activation is limited [2]. In humans, several IFNs have been discovered (e.g. IFN-α/β/ε/κ/ω/γ/λ [3-8]). IFN-α, IFN-β, IFN-ε, IFN-κ, IFN-ω are grouped into type I IFNs for signalling through the common IFN-α receptor (IFNAR) complex present on target cells [3-6] (**Figure 1A**). IFN-α comprises 13 subtypes in humans while the remaining type I IFNs are encoded by a specific gene [9]. IFN-λ targets IFN-λ receptor 1 (IFNLR1)/interleukin-10 receptor 2 (IL-10R2) and was classified as type III IFN since its discovery in 2003 [8] (**Figure 1C**). Similar to type I IFNs, IFN-λ also exert antiviral properties but functions less intensely

[10-12]. IFN-γ is classified as type II IFN and manifest its biological effects by interacting with IFN-γ receptor (IFNGR) [7] (**Figure 1B**). In contrast to type I and III IFNs, IFN-γ is also anti-pathogen, immunomodulatory, and proinflammatory but more focused on establishing cell immunity [3,7,11,13].

All three types of IFNs are capable of activating the Janus kinase/signal transducer and activator of transcription (JAK-STAT) pathway and inducing the transcriptional up-regulation of approximately 10% of human genes that prime cells for stronger pathogen detections and defenses [9,14,15]. Henceforth, these up-regulated human genes are referred to as the IFN-stimulated genes (ISGs). They play an important role in the establishment of the cellular antiviral state, the inhibition of viral infection and the return to cellular homeostasis [3,9,14,16]. For example, the ectopic expression of heparinase (HPSE) can inhibits the attachment of multiple viruses [17,18]; interferon induced transmembrane proteins (IFITM) can impair the entry of multiple viruses and traffic viral particles to degradative lysosomes [19,20]; MX dynamin like GTPase proteins (MX) can effectively block early steps of multiple viral replication cycles [21]. Abnormality in the IFN-signalling cascade, for example, the absence of signal transducer and activator of transcription 1 (STAT1) will lead to the failure of activating ISGs, making the host cell highly susceptible to virus infections [22].

Figure 1. Illustration of signalling cascade triggered by different IFNs. In (A), type I IFN signals through IFNAR, Janus kinase 1(JAK1), tyrosine kinase 2 (TYK2), STAT, and IFN regulatory factor 9 (IRF9) to form IFN stimulated gene factor 3 complex (ISGF3), and then bind to IFN stimulated response elements (ISRE) to induce the expression of type I ISGs. In (B), type II IFN signals through IFNGR, JAK1 and JAK2 to form IFN-γ activation factor (GAF) and then bind to gamma-activated sequence promoter elements (GAS) to induce the expression of type II ISGs. In (C), type III IFN signals through IFNLR1, IL-10R2, JAK1, TYK2, STAT,

and IRF9 to form ISGF3, and then bind to ISRE to induce the expression of type III ISGs. Figure created using the BioRender (https://biorender.com/).

Most research on the ISGs has focused on elucidating the role of the ISGs in antiviral activities or discovering new ISGs within or across species [3,9,14,19,23,24]. The identification of ISGs can be achieved via various approaches. Associating gene expression with suppression of viral infection is a good strategy to identify ISGs with obvious antiviral performance, exemplified by the influenza inhibitor, MX dynamin like GTPase 1 (MX1), and the human immunodeficiency virus 1 inhibitor, MX dynamin like GTPase 2 (MX2) [21]. CRISPR screening is a loss-of-function experimental approach to identify ISGs required for IFN-mediated inhibition to viruses. It enabled the discovery of tripartite motif containing 5 (TRIM5), MX2 and bone marrow stromal cell antigen 2 (BST2) [25]. Monitoring the ectopic expression of ISGs is another instrumental way to find some ISGs that are individually sufficient for viral suppression [26], for example, interferon stimulated exonuclease gene 20 (ISG20) and ISG15 ubiquitin like modifier (ISG15). Using RNA-sequencing [27] and fold change-based criterion to measure whether a target human gene is induced by IFN signalling now has become a well-accepted idea [24,28,29]. In most cases, a gene is defined as IFN stimulated (up-regulated) when its expression value is more than doubled with the presence of IFNs (fold change > 2) [3,24,30]. There are also many online databases to support IFN- or ISG-related research. For example, Interferome (http://www.interferome.org) provides an excellent resource by compiling *in vivo* and *in vitro* gene expression profiles in the context of IFN stimulation [24]. The Orthologous Clusters of Interferon-stimulated Genes (OCISG, http://isg.data.cvr.ac.uk) demonstrates an evolutionary comparative approach of genes differentially expressed in type I IFN system for ten different species [3].

We notice that a same human gene may show differential response to different IFNs in different tissues or cells [24]. Despite some well-investigated ISGs, the majority of classified ISGs have limitedly expression following IFN stimulations [3,24]. It means that the difference between ISGs and those human genes not significantly up-regulated in the presence of IFNs (non-ISGs) may not be obvious especially when being assessed more generally. It should also be noted that, within non-ISGs, there are a group of genes down-regulated during IFN stimulations. We refer to them as interferon-repressed human genes (IRGs) and they constitute another major part of the IFN regulation system [3,31]. Collectively, the complex nature of the IFN-stimulated system results in knowledge that is far from comprehensive.

In this study, we try to associate the inherent properties of human genes with their expression following IFN-α stimulations. We propose that it is feasible to make ISG predictions on human genes with a model only compiled from the knowledge of IFN-α responses in the human fibroblast cells. To achieve these ends, we first constructed a refined high-confidence dataset consisting of 620 ISGs and 874 non-ISGs by checking the genes across multiple databases including the OCISG [3], Interferome [24], and Reference Sequence (RefSeq) [32]. The analyses were conducted primarily on our refined data using genome- and proteome-based features that were likely to influence the expression of human genes in the presence of IFN-α. Then based on the calculated features, we designed a machine learning framework with an optimised feature selection strategy for the prediction of putative ISGs in different IFN systems. Finally, we also developed an online web server that implemented our machine learning method at http://isgpre.cvr.gla.ac.uk/.

Results

Evolutionary characteristics of ISGs

In this study, we constructed the dataset S2 from 10836 well-annotated human genes (dataset S1). It consists of 620 ISGs and 874 non-ISGs with high confidence based on their records in both the OCISG [3] and Interferome [24]. The compiled 10836 human genes were used as the background set and were evolutionarily unrelated to each other as they were retrieved from the OCISG [3] that compiled clusters of orthologous genes based on whole-genome alignments. Detailed information about our compiled datasets is provided in **Table 5** and **Supplementary Data S1**.

Here, we explored features relating to alternative splicing [33], duplication [34] and mutation [35]. We used the number of open reading frames (ORFs) and transcripts of a human gene to represent the diversity of its alternative splicing process. Meanwhile, the usage of protein-coding exons was quantified to reflect the complexity of the alternative splicing process. By calculating the average number of ORFs with respect to different Log_2(Fold Change) levels of expression (window size = 0.1) in the presence of IFN-α, we found that more highly upregulated human genes tended to have less ORFs (Pearson’s correlation coefficient (PCC) = -0.287, **Figure 2A**). As for the latter two features relating to the transcripts and protein-coding exons, similar negative relationships were observed when Log_2(Fold Change) increased (**Figure 2B & 2C**). These results illustrate that simple alternative splicing process may promote IFN-α up-regulation. Particularly, as the lowest value of Log_2(Fold Change) for human genes not differentially expressed only reached around -0.9. Points placed left to the boundary (x = -0.9) are all IRGs. They are generally placed below those non-ISGs with a Log_2(Fold Change) around zero, suggesting these three features (number of ORFs, number of transcripts and the usage of protein-coding exons) are all differentially represented in some IRGs compared to the remaining non-ISGs. This distribution also indicates that some IRGs have similar feature

patterns to ISGs, especially to those highly up-regulated in the presence of IFN-α (right part of the scatter plots in **Figure 2A, 2B & 2C**).

Figure 2. The average representation of features associated with IFN-α stimulations in experiments. (A) The numbers of ORFs and (B) transcripts are used as measurements of the diversity of alternative splicing process. (C) The counts of exons used for coding is used as a measurement of the complexity of alternative splicing process. These three plots are drawn based on the expression data of 8619 human genes with valid fold change in the IFN-α experiments (**Supplementary Data S1**). 2217 human genes are not shown in these figures as they had insufficient read coverage to determine a fold change in the experiments (**Table 5**). Points in the scatter plot are located based on the average feature representation of genes with similar expression performance in experiments.

To determine whether the ISGs tend to originate from duplications, we counted the number of within human paralogs of each gene (**Figure 3A**). We found that there were around 22% of singletons in our main dataset, whilst ISGs had 15% and non-ISGs had 26%. The result of a Mann-Whitney U test [36] indicated that the number of human paralogs was significantly under-represented in the ISGs compared to the background human genes ($M_1 = 10.5$, $M_2 = 11.5$, $p = 8.8E\text{-}03$). We hypothesize that such a difference is mainly caused by the imbalanced distribution of singletons in the ISGs and non-ISGs as it becomes smaller when singletons are excluded from the test ($M_1 = 12.4$, $M_2 = 14.6$, $p > 0.05$). Next, we used the number of non-synonymous substitutions per non-synonymous site (dN) and synonymous substitutions per synonymous site (dS) within human paralogues as a measurement of differences in mutational signatures between different classes [37]. As shown in **Figure 3B**, non-synonymous substitutions are more frequently observed in the ISGs than in the background human genes

(M_1 = 0.62, M_2 = 0.55, p = 4.0E-03). On the other hand, the ISGs also have a higher frequency of synonymous substitutions than the background human genes (M_1 = 37.7, M_2 = 34.6, p = 1.1E-02) (**Figure 3C**) but the difference is not as obvious as for non-synonymous substitutions. In **Figure 3D**, the distribution of dN/dS ratios within human paralogues indicates that most human genes are constrained by natural selection but the ISGs, in general, tend to be less conserved (M_1 = 0.036, M_2 = 0.045, p = 8.3E-03). When eliminating the influence of duplication events, the ISGs are still less conserved than the non-ISGs but the difference in the dN/dS ratio is not significant (M_1 = 0.053, M_2 = 0.031, $p > 0.05$).

Figure 3. Differences in the evolutionary constraints of human genes. (A) Paralogues within *Homo sapiens*. (B) Non-synonymous substitutions within human paralogues. (C) Synonymous substitutions within human paralogues. (D) dN/dS ratios within human paralogues. Here, the ISGs and non-ISGs are taken from dataset S2 while the background human genes are from dataset S1 (**Table 5**). Mann-Whitney U tests are applied for the hypothesis testing between the feature distribution of different classes. Boxes in the plot represent the major distribution of values (from the first to the third quartile); outliers are added for values higher than two-fold of the third quartile; cross symbol marks the position of the average value including the outliers; upper and lower whiskers show the maximum and minimum values excluding the outliers.

Differences in the coding region of the canonical transcripts

Compared to general profile features (e.g., number of ORFs), the sequences themselves provide more direct mapping to the protein function and structure [38]. Here, we encoded 344 parametric features and 7026 non-parametric features from complementary DNA (cDNA) of the canonical transcript to explore features specific to ISGs. We divided the parametric features

into four categories and compared their representations among three different groups of human genes including recompiled ISGs from dataset S2, recompiled non-ISGs from dataset S2, and the background human genes from dataset S1 (**Figure 4**). Firstly, guanine and cytosine were both more depleted in ISGs than non-ISGs, leading to an under-representation of GC-content in the ISGs (Mann-Whitney U test: M_1 = 52%, M_2 = 55%, p = 2.3E-11). This attribute was antithetical to the GC-biased gene conversion (gBGC), making ISGs less stable with weak evolutionary conservation (**Figure 3**) [39]. Additionally, the under-representation of GC-content also influenced the representation of other dinucleotide features. Among all dinucleotide depletions in ISGs, CpG composition was ranked the first followed by GpG and GpC composition (p = 2.9E-14, 4.9E-13 and 1.2E-10, respectively). In turn, adenine and thymine-related dinucleotide compositions, exemplified by ApT and TpA were more enriched in ISGs than non-ISGs (p = 8.0E-10 and 8.5E-10, respectively).

We compared the usage of 64 different codons in the third category as their frequencies influence transcription efficiency [40]. Differences between the ISGs and background human genes were observed in codons for 11 amino acids including leucine (L), isoleucine (I), valine (V), serine (S), threonine (T), alanine (A), glutamine (Q), lysine (K), glutamic acid (E), arginine (R), and glycine (G). The most significant difference was observed in the usage of codon 'AGA'. Among all arginine-targeted alternative codons, codon 'AGA' was usually favoured, and its usage reached an estimated 25% in the ISGs but reduced to 22% in the background human genes (p = 1.4E-05). It was even significantly lower in the non-ISGs, at 18% (p = 1.9E-13). On the other hand, compared to the background human genes, the codon 'CAG' coding for amino acid 'Q' was the most under-represented in the ISGs. It was less favoured by the ISGs than non-ISGs (M_1 = 72%, M_2 = 78%, p = 7.3E-13) although it dominated in coding patterns. As for the three stop codons, comparing with the background human genes, the usage of the ochre stop codon ('TAA') was over-represented in the ISGs (M_1 = 28%, M_2 = 33%, p =

9.7E-03). In this category of codon usage, the features with different frequencies between the ISGs and background human genes became more discriminating when comparing the ISGs with non-ISGs. Significant differences in codon usages between the ISGs and non-ISGs were widely observed except for methionine (M) and tryptophan (W). Hence, despite the limited differences of codon usages between the ISGs and background human genes, these features were useful for discriminating the ISGs from non-ISGs.

In the last category, we calculated the occurrence frequency of 256 nucleotide 4-mers to add some positional resolution for finding and comparing interesting organisational structures [41]. Among the 256 4-mers, 46 of them were differentially represented between the ISGs and background human genes (**Supplementary Data S2**). Most of these 4-mers were over-represented by the ISGs except two with the pattern 'TAAA' and 'CGCG'. Interestingly, the feature of 'TAAA' composition became a positive factor when comparing ISGs and non-ISGs (M_1 = 4.1%, M_2 = 3.7%, p = 4.1E-06), suggesting it might be a good feature to discern potential or incorrectly labelled ISGs. We found six nucleotide 4-mers: 'ACCC', 'AGTC', 'AGTG', 'TGCT', 'GACC', and 'GTGC' were over-represented in the ISGs when compared to the background human genes. However, they were not differentially represented when comparing the ISGs with non-ISGs. These six features might be inherently biased for some reasons and were not powerful enough to distinguish the ISGs from non-ISGs. In addition to the aforementioned 40 features (except 4-mer 'ACCC', 'AGTC', 'AGTG', 'TGCT', 'GACC', and 'GTGC') that were differentially represented in ISGs compared to background human genes, we found a further 39 features nucleotide 4-mers differentially represented between ISGs and non-ISGs (**Supplementary Data S2**).

To check the effect of these aforementioned 343 features on the level of stimulation in the IFN-α system (Log_2(Fold Change) > 0), we calculated the PCC for the normalised features (**Equation 2**) and found 106 features were positively related to the increase of fold change, and

34 features were suppressed when human gene were more up-regulated after IFN-α treatments (Student t-test: $p < 0.05$) (**Supplementary Data S3**). ApA composition showed the most obvious positive correlation with stimulation level (PCC = 0.464, p = 8.8E-06) while negative association between the representation of 4-mer 'CGCG' and IFN-α-induced up-regulation was the most significant (PCC = -0.593, p = 3.2E-09). Human genes with higher up-regulation in the presence of IFN-α contained more codons 'CAA' rather than 'CAG' for coding amino acid 'Q'. The depletion of GC-content, especially cytosine content, promotes the suppression of many nucleotide compositions in the cDNA, e.g. CpG composition.

Figure 4. Differences in the representation of parametric features encoded from coding regions (canonical). Mann-Whitney U tests are applied for hypothesis testing and the results are provided in the **Supplementary Data S2**. Here, the ISGs and non-ISGs are taken from dataset S2 while the background human genes are from dataset S1 (**Table 5**).

To find conserved sequence patterns relating to gene regulations [42], we checked the existence of 2940, 44100 and 661500 short linear nucleotide patterns (SLNPs) consisting of three to five consecutive nucleobases in the group of the ISGs and non-ISGs. By using a positive 5% difference in the occurrence frequency as cut-off threshold, we found 7884 SLNPs with a maximum difference in representation around 15%. After using Pearson's chi-squared tests and Benjamini-Hochberg correction to avoid type I error in multiple hypotheses [43], 7025 SLNPs remained with an adjusted p-value lower than 0.01 (**Supplementary Data S4**), hereon referred to as flagged SLNPs. The differentially represented 7025 SLNPs were ranked according to the adjusted p-value. As shown in **Figure 5A**, dinucleotide 'TpA' dominates in the top 10, top 100, top 1000, and all differentially represented SLNPs even if TpA representation is suppressed in the cDNA of genes' canonical transcripts compared to other

dinucleotides. Dinucleotide ‘ApT’ and ‘ApA’ are also frequently observed in the flagged SLNPs but their occurrences do not show significant difference in the top 100 SLNPs (Pearson's chi-squared test: $p > 0.05$). GC-related dinucleotides, e.g., ‘CpC’, ‘GpC’ and ‘GpG’ are rarely observed in the flagged SLNPs especially in the top 10 or top 100. In view of these, we hypothesize that the differential representation of nucleotide compositions influences and reflects on the pattern of SLNPs in the ISGs. By checking the co-occurrence status of the flagged SLNPs, we found that these sequence patterns had a cumulative effect in distinguishing the ISGs from non-ISGs especially when the number of cooccurring SLNPs reached around 5320 (Pearson's chi-squared test: $p = 7.9E-13$, **Figure 5B**). There were eight (~1.3%) ISGs in the dataset S2 containing all the flagged 7025 SLNPs. Their up-regulation after IFN-α treatment were generally low with a fold change fluctuating around 2.2. However, some of these eight genes such as desmoplakin (DSP) were clearly highly up-regulated in endothelial cells isolated from human umbilical cord veins after not only IFN-α treatments (fold change = 11.1) but also IFN-β treatments (fold change = 13.7). We also found some non-ISGs (e.g., hemicentin 1 (HMCN1)) and human genes with limited expression in the IFN-α experiments (ELGs) (e.g. tudor domain containing 6 (TDRD6)) containing the flagged SLNPs, but their frequencies were lower than that in the ISGs. Although there is an obvious imbalance between the number of the ISGs and non-ISGs in the human genome [9-11], the curve for the background human genes in **Figure 5B** is still closer to that for the ISGs rather than that for the non-ISGs. It suggests that some genetic patterns are widely represented in the coding region of human genes, making them potentially up-regulated in the IFN-α system.

Figure 5. SLNPs in the coding regions (canonical). (A) Influence of dinucleotide compositions on the flagged SLNPs. (B) The co-occurrence status of SLNPs in different human genes. Ranks in (A) are generated based on the adjust p value given by Pearson's chi-squared

tests after Benjamini-Hochberg correction procedure. Detailed results of the hypothesis tests are provided in **Supplementary Data S4**. Here, the ISGs and non-ISGs are taken from dataset S2 while the background human genes are from dataset S1 (**Table 5**).

Differences in the protein sequence

We used the protein sequences generated by the canonical transcript to extract features at the proteomic level. In addition to the basic composition of 20 standard amino acids, we considered 17 additional features related to physicochemical (e.g., hydropathy and polarity) or geometric properties (e.g., volume) [44,45]. We found several amino acids that were either enriched or depleted in the ISG products compared to the background human proteins, which were produced by genes in dataset S1 (**Figure 6**). The differences were even more marked between protein products of the ISGs and non-ISGs, highlighting some differences that were not observed when comparing the ISG products to the background human proteins (e.g., isoleucine composition). The differences observed in the amino acid compositions were at least in part associated with the patterns previously observed in features encoded from genetic coding regions. For example, asparagine (N) showed significant over-representation in the ISG products compared to the non-ISG products or background human proteins (Mann-Whitney U test: p = 2.8E-12 and 1.2E-03, respectively). This was expected as there are only two codons, i.e., 'AAT' and 'AAC' coding for amino acid 'N', and dinucleotide 'ApA' showed a remarkable enrichment in the coding region of ISGs. A similar explanation could be given for the relationship between the deficiency of GpG content and amino acid 'G'. The translation of amino acid 'K' was also influenced by ApA composition but was not significant due to the mild representation of dinucleotide 'ApG' in the genetic coding region. Additionally, as previously mentioned, the ISGs showed a significant depletion in the CpG content, and consequently, the amino acid 'A' and 'R' in the ISG products were significantly under-

represented. Cysteine (C) was not frequently observed in human proteins but still showed a relatively significant enrichment in the ISG products (M_1 = 2.3%, M_2 = 2.5%, p = 1.8E-03).

When focusing on the composition of amino acids grouped by physicochemical or geometric properties, we found some features differentially represented between the ISG products and background human proteins. The result showed that hydroxyl (amino acid 'S' and 'T'), amide (amino acid 'N' and 'Q'), or sulfur amino acids (amino acid 'C' and 'M') were more abundant in the ISG products compared to the background human proteins (Mann-Whitney U test: p = 0.04, 1.0E-03 and 0.02, respectively). Small amino acids (amino acid 'N', 'C', 'T', aspartic acid (D) and proline (P), the volume ranges from 108.5 to 116.1 cubic angstroms) were more frequently observed in the ISG products than in background human proteins (M_1 = 22.1%, M_2 = 21.7%, p = 0.02). These differences became more marked when comparing the representation of these features between the ISG and non-ISG products. For example, features relating to chemical properties of the side chain (e.g., aliphatic), charge status and geometric volume showed differences between proteins produced by the ISGs and non-ISGs. Some features such as neutral amino acids that include amino acid 'G', 'P', 'S', 'T', histidine (H) and tyrosine (Y) were not differentially represented between the ISG and non-ISG products, but they indicated obvious association with the change of IFN-α-triggered stimulations (PCC = -0.556, p = 4.1E-08) (**Supplementary Data S3**).

Figure 6. Differences in the representation of parametric features encoded from protein sequences. Mann-Whitney U tests are applied for hypothesis testing and the results are provided in the **Supplementary Data S2**. Here, the ISGs and non-ISGs are taken from dataset S2 while the background human genes are from dataset S1 (**Table 5**). Aliphatic group: amino acid 'A', 'G', 'I', 'L', 'P' and 'V'; aromatic/huge group: amino acid 'F', 'W' and 'Y' (volume > 180 cubic angstroms); sulfur group: amino acid 'C' and 'M'; hydroxyl group: amino acid 'S'

and ‘T’; acidic/negative_charged group: amino acid ‘D’ and ‘E’; amide group: amino acid ‘N’ and ‘Q’; positive_charged group: amino acid ‘R’, ‘H’ and ‘K’; hydrophobic group: amino acid ‘A’, ‘C’, ‘I’, ‘L’, ‘M’, ‘F’, ‘V’, and ‘W’ that participates to the hydrophobic core of the structural domains [46]; neutral group: amino acid ‘G’, ‘H’, ‘P’, ‘S’, ‘T’ and ‘Y’; hydrophilic group: amino acid ‘R’, ‘N’, ‘D’, ‘Q’, ‘E’ and ‘K’; Tiny group: amino acid ‘G’, ‘A’ and ‘S’ (volume < 90 cubic angstroms); small group: amino acid ‘N’, ‘D’, ‘C’, ‘P’ and ‘T’ (volume ranged from 109 to 116 cubic angstroms); medium group: amino acid ‘Q’, ‘E’, ‘H’ and ‘V’ (volume ranged within 138 to 153 cubic angstroms); large group: amino acid ‘R’, ‘I’, ‘L’, ‘K’ and ‘M’ (volume ranged within 163 to 173 cubic angstroms); uncharged group: the remaining 15 amino acids except electrically charged ones; polar group: amino acid ‘R’, ‘H’, ‘K’, ‘D’, ‘E’, ‘N’, ‘Q’, ‘S’, ‘T’ and ‘Y’; nonpolar group: the remaining 10 amino acids except polar ones.

Next, we searched the sequence of the ISG products against that of the non-ISG products to find conserved short linear amino acid patterns (SLAAPs), which might have resulted from strong purifying selection [47]. As opposed to the analysis on the genetic sequence, we only obtained 19 enriched sequence patterns with a Pearson's chi-squared p value ranging from 1.5E-04 to 0.02 (**Table 1**), hereon referred to as flagged SLAAPs. They were greatly influenced by four polar amino acids: ‘K’, ‘N’, ‘E’ and ‘S’, and one nonpolar amino acid: ‘L’. Some of these flagged SLAAPs, for example, SLAAP ‘NVT’ and ‘S-N-E’, were clearly over-represented in the ISG products compared to the background human proteins and could be used as features to differentiate the ISGs from background human genes. The third column in **Table 1** indicates a number of patterns that are lacking in the non-ISG products and hence may be the reason for the lack of up-regulation in the presence of IFN-α. Particularly, we noticed that SLAAP ‘KEN’ was a destruction motif that could be recognised or targeted by anaphase promoting complex (APC) for polyubiquitination and proteasome-mediated

degradation [48,49]. Results shown in **Figure 7A** illustrate that the co-occurrence of differentially represented SLAAPs (flagged) has a cumulative effect in distinguishing the ISGs from non-ISGs. This cumulative effect can even be achieved with only two random SLAAPs (Pearson's chi-squared test: p = 4.6E-10). The bias in the co-occurring SLAAPs (flagged) in the background human proteins towards a pattern similar to the non-ISG products further proves the importance of these 19 SLAAPs. However, their co-occurrence is not associated with the level of IFN-triggered stimulations (PCC = 0.015, $p > 0.05$) (**Figure 7B**).

Regions that lacked stable structures under normal physiological conditions within proteins are termed intrinsically disordered regions (IDRs). They play an important role in cell signalling [50]. Compared with ordered regions, IDRs are usually more accessible and have multiple binding motifs, which can potentially bind to multiple partners [51]. According to the results calculated by IUPred [52], we found 6721, 10510, and 119071 IDRs (IUpred score no less than 0.5) in proteins produced by the ISGs, non-ISGs and background human genes respectively. We hypothesize that enriched SLAAPs widely detected in the IDRs may be important for human protein-protein interactions or potentially virus mimicry [53]. For instance, in the ISG products, about 40.8% of SLAAP 'SxNxT' were observed in the IDRs, 14.9% higher than that in non-ISG products (**Table 1**). This difference reflected the importance of SLAAP 'SxNxT' for target specificity of IFN-α-induced protein-protein interactions (PPIs) [9] even if it was not statistically significant. By contrast, the conditional frequency of SLAAP 'SxNxE' in the IDRs of the ISG and non-ISG products were almost the same, indicating that SLAAP 'SxNxE' might have an association with some inherent attributes of the ISGs but was less likely to be involved in the IFN-α-induced PPIs. SLAAP 'KEN' in the IDRs also showed some interesting differences: in the non-ISG products, 41.9% of SLAAP 'KEN' were observed in the IDRs, 14.6% higher than that in the ISG products, which provided an effective approach to distinguish the ISGs from non-ISGs. When SLAAP 'KEN' is discovered in the ordered

globular region of a protein sequence, statistically, the protein is more likely to be produced by an ISG, but this assumption is reversed if the SLAAP is located in an IDR (Pearson's chi-squared tests: $p = 0.03$). Despite the relatively low conditional frequency of SLAAP 'KEN' in the IDRs of the ISG products, these SLAAPs in the IDR are more likely to be functionally active than those falling within ordered globular regions [54].

Table 1. Representation of SLAAPs in protein sequences and their IDRs.

SLAAP[a]	Frequency in ISG/non-ISG products[b]	Bias based on the frequency in human proteins	P value[c]	Conditional frequency in the IDRs of ISG/non-ISG products/background human proteins[c,d]	P value[e]
SxNxE	15.2%/8.8%	+47.6%/-14.2%	1.5E-04	39.4%/40.3%/33.4%	0.90
ENE	15.0%/8.8%	+20.9%/-29.0%	2.1E-04	37.6%/42.9%/40.9%	0.49
SxNxT	11.5%/6.2%	+21.9%/-34.2%	2.9E-04	40.8%/25.9%/27.3%	0.08
SVI	15.2%/9.2%	+37.6%/-16.9%	3.6E-04	18.1%/11.3%/15.2%	0.21
LxNL	23.7%/16.4%	+13.2%/-21.9%	4.0E-04	10.2%/11.9%/9.4%	0.65
LxKL	30.8%/22.8%	+18.0%/-12.8%	4.9E-04	12.6%/10.1%/8.7%	0.43
NVT	13.7%/8.5%	+52.1%/-6.1%	1.2E-03	18.8%/21.6%/15.4%	0.66
ISS	20.5%/14.3%	+20.7%/-15.7%	1.7E-03	29.9%/25.6%/23.8%	0.44
LKxK	24.4%/17.7%	+24.5%/-9.3%	1.8E-03	14.6%/20.6%/20.0%	0.16
IKxE	14.2%/9.0%	+34.2%/-14.5%	1.8E-03	26.1%/16.5%/25.8%	0.13
EKxI	15.8%/10.4%	+31.0%/-13.7%	2.0E-03	15.3%/20.9%/16.0%	0.32
KxExS	16.9%/11.4%	+21.9%/-17.7%	2.4E-03	36.2%/36.0%/39.2%	0.98
LNS	17.7%/12.1%	+21.2%/-17.1%	2.4E-03	20.0%/25.5%/20.5%	0.34
KEN	16.0%/10.6%	+33.5%/-11.0%	2.4E-03	27.3%/41.9%/34.8%	0.03
LxNxL	22.6%/17.5%	+14.3%/-11.4%	1.5E-02	10.7%/11.8%/9.5%	0.78
KxExL	25.8%/20.5%	+25.7%/-0.3%	1.5E-02	18.8%/17.9%/18.7%	0.84
KLL	27.1%/21.9%	+9.9%/-11.4%	1.9E-02	11.3%/8.4%/9.9%	0.35
LKE	29.8%/24.5%	+18.2%/-3.0%	2.1E-02	19.5%/24.8%/20.1%	0.20
LKxL	33.2%/27.7%	+15.0%/-4.2%	2.1E-02	7.8%/12.4%/10.0%	0.11

a: 'x' in SLAAPs indicates one position occupied by a standard amino acid;

*b: here, the ISGs and non-ISGs are taken from dataset S2 while the background human genes use samples from dataset S1 (**Table 5**);*

c: p values in this column use Pearson's chi-squared tests to measure the difference of SLAAPs occurrences in the ISG and non-ISG products;

d: frequencies in this column are calculated based on a condition that corresponding SLAAPs are observed in the protein sequence;

e: p values in this column use Pearson's chi-squared tests to measure the difference of SLAAPs occurrences in the IDRs of the ISG and non-ISG products.

Figure 7. Representation of co-occurred SLAAPs (flagged) in our main dataset. (A) The co-occurrence status of SLAAPs in different classes. (B) Relationship between co-occurrence of the marked SLAAPs and Log_2(Fold Change) after IFN-α treatments. Here, the ISGs and non-ISGs are taken from dataset S2 while the background human genes are from dataset S1 (**Table 5**). Points in (B) are located based on the average feature representation of genes with similar expression performance in IFN-α experiments.

Differences in network profiles

We constructed a network with 332,698 experimentally verified interactions among 17603 human proteins (confidence score > 0.63) from the Human Integrated Protein-Protein Interaction rEference (HIPPIE) database [55] to investigate if the connectivity among human proteins have association with genes' expression in the IFN-α experiments. 10169 out of 10836 human proteins produced by genes in our background dataset S1 were included in the network. Nodes and edges of this network can be downloaded from our webserver at http://isgpre.cvr.gla.ac.uk/. Based on this network, we calculated eight features including the average shortest path, closeness, betweenness, stress, degree, neighbourhood connectivity, clustering coefficient, and topological coefficient.

As illustrated in **Figure 8B/G**, ISG products tend to have higher values of betweenness and stress than background human proteins (Mann-Whitney U test: p = 0.01, and 0.03, respectively), which means they are more likely to locate at key paths connecting different nodes of the PPI network. Some ISG products with high values of betweenness and stress, e.g., tripartite motif containing 25 (TRIM25), can be considered as the shortcut or bottleneck of the network and play important roles in many PPIs including those related to the IFN-α-triggered immune activities [56,57]. However, such differential representation of betweenness does not mean ISG products are more likely to be or even be close to bottlenecks of the network compared to the background human proteins. Some examples shown in **Table 2** indicate that ISG products are less-connected by top-ranked bottlenecks and hubs of the network than non-ISG products or the background human proteins. This conclusion is not influenced by hub/bottleneck protein's performance in the IFN-α experiments. Comparing proteins produced by the ISGs and non-ISGs, we found the former tends to have lower values of clustering coefficient and neighbourhood connectivity (Mann-Whitney U test: p = 0.04 and 7.9E-03, **Figure 8D/F**). This discovery indicates that the ISG products and some of their interacting proteins are less likely to be targeted by lots of proteins. It also supports the finding that the ISG products are involved in many shortest paths for nodes but are away from hubs or bottlenecks in the network. To some extents, this location also increases the length of the average shortest paths through ISG products in the network (**Figure 8A**).

When investigating the association between IFN-α-induced gene stimulation and network attributes of gene products, we only found the feature of neighbourhood connectivity was under-represented as the level of differential expression in the presence of IFN increases (PCC = -0.392, p = 2.2E-04). This suggests that proteins produced by genes that are highly up-regulated in response to IFN-α are further away from hubs in the PPI networks.

Figure 8. Differences in network preferences. The included features are: (A) average shortest path (B) betweenness, (C) closeness, (D) clustering coefficient, (E) degree, (F) neighbourhood connectivity, (G) stress, and (H) topological coefficient. Mann-Whitney U tests are applied for hypothesis testing and the results were provided in the **Supplementary Data S2**. Here, the ISGs and non-ISGs are taken from dataset S2 while the background human genes use samples from dataset S1 (**Table 5**).

Table 2. Interaction profiles of human proteins connecting top hubs/bottlenecks of the HIPPIE network.

Human protein	TRIM25	ELAVL1	ESR2	NTRK1
Gene class	ISG	IRG	Not included in S1[a]	
Degree (hub rank)	2295 (2nd)	1787 (4th)	2500 (1st)	1976 (3rd)
Betweenness (bottleneck rank)	0.067 (1st)	0.048 (4th)	0.051 (3rd)	0.026 (5th)
Difference in interacting partners (ISG products versus non-ISG products)[b]	Depleted P = 0.01	P > 0.05	Depleted P = 1.1E-4	Depleted P = 5.5E-3
Difference in interacting partners (ISG products versus the background human proteins)[b]	P > 0.05	P > 0.05	Depleted P = 8.1E-3	Depleted P = 0.03

a: ESR2 and NTRK1 were not included in dataset S1 as their expression data were not compiled in OCISG;

b: differences here are measured via Pearson's chi-squared tests on human proteins interacting with the corresponding hub/bottleneck protein.

Features highly associated with the level of IFN stimulations

In this study, we encoded a total of 397 parametric and 7046 non-parametric features covering the aspects of evolutionary conservation, nucleotide composition, transcription, amino acid composition, and network profiles. In order to find out some key factors that may enhance or suppress the stimulation of human genes in the IFN-α system, we compared the representation of parametric features of human genes with different but positive Log_2(Fold Change). Two features on the co-occurrence of SLNPs and SLAAPs were not taken into consideration here

as they were more subjective than the other parametric features and were greatly influenced by the number of sequence patterns. Upon the calculation of PCC and the result of hypothesis tests, we found 168 features highly associated with the level of IFN-α-triggered stimulations (Student t-tests: $p < 0.05$) (**Supplementary Data S3**). Among them, 118 features showed a positive correlation (**Figure 9**) while the remaining 50 features showed a negative correlation (**Figure 10**) with the change of up-regulation in IFN-α experiments. Among these 168 features, the number of ORFs, alternative splicing results, and counts of exons used for coding were encoded from characteristics of the gene. Average dN/dS and average dS within human paralogues were encoded based on the sequence alignment results from Ensembl [58]. 140 and 22 features were encoded from the genetic sequence and proteomic sequence respectively. The last one, neighbourhood connectivity, was obtained from the network profile of a human interactome constructed based on experimentally verified data in the HIPPIE database [55].

In the positive group, the feature of 'large' amino acid compositions that includes the composition of five amino acids with geometric volume ranged from 163 to 173 cubic angstroms was ranked the first for having the highest PCC at 0.593 (Student t-test: p = 2.8E-09). This feature was not highlighted previously as it did not have a strong signal for discriminating the ISGs from non-ISGs (Mann-Whitney U test: $p > 0.05$). Similar phenomena were found on 87 features (64 positive correlations and 23 negative correlations) such as AG-content, ApG content and previously mentioned neutral amino acid composition. The strongest negative correlation between feature representation and IFN-α-triggered stimulations was found on the feature of 4-mer 'CGCG' (PCC = -0.593, p = 3.2E-09). This feature also showed a differential distribution between the ISGs and non-ISGs, thus provided useful information to distinguish the ISGs from non-ISGs. Similar phenomena were found on 81 features (54 positive correlations and 27 negative correlations) such as previously mentioned GC-content, CpG content and the usage of codon 'GCG' coding for amino acid 'A'.

Collectively, the biased effect on the basic composition of nucleotides influences the correlation between the representation of sequence-based features and IFN-α-triggered stimulations. Human genes that show over-representation in more features listed in **Figure 9** are expected to be more up-regulated after IFN-α treatments at least in the human fibroblast cells. Meanwhile, the under-representation of features listed in **Figure 10** also contributes to the level of up-regulation in the IFN-α experiments.

Figure 9. 118 features positively associated with higher up-regulation after IFN-α treatments. Features here are screened based on the PCC and results of Student t-tests ($p < 0.05$). Detailed results about PCC and hypothesis tests are provided in **Supplementary Data S3**.

Figure 10. 50 features negatively associated with higher up-regulation after IFN-α treatments. Features here are screened based on the PCC and results of Student t-tests ($p < 0.05$). Detailed results about PCC and hypothesis tests are provided in **Supplementary Data S3**.

Difference in feature representation of interferon-repressed genes and genes with low levels of expression

We grouped human genes into two classes based on their response to the IFN-α in the human fibroblast cells. Genes significantly up-regulated in IFN-α experiments were included in the ISG class, while those that did not were put into the non-ISG class. However, there is also another group of human genes down-regulated in the presence of IFN-α, i.e., the IRGs. They were labelled as the non-ISGs, but contain unique patterns that constitute an important aspect of the IFN response [3]. Some of these IRGs were not up-regulated in any known type I IFN

systems, thus have been placed in a refined non-ISG class for analyses and predictions. Additionally, there are a number of genes that have insufficient levels of expression in the experiments to determine a fold change, i.e., ELGs. Here, we used the previously defined features to compare the ISGs from dataset S2 with the IRGs and ELGs divided from the background dataset S1 (**Table 5**).

As shown in **Figure 11,** the IRGs are differentially represented to a lower extent in the majority of nucleotide 4-mer compositions than the ISGs, indicating the deficiency of some nucleotide sequence patterns in the coding region of IRGs. Note that, many nucleotide 4-mer composition features are more suppressed in the ISGs than non-ISGs although the differences are small. The biased representation of these features in the IRGs suggests that the IRGs have characteristics similar to the ISGs rather than non-ISGs. Additionally, there are a very limited number of features relating to evolutionary conservation, nucleotide compositions or codon usages showing obvious differences between the ISGs and IRGs, but many of them are differentially represented when comparing the ISGs with non-ISGs. Therefore, involving the IRGs in the class of the non-ISGs will increase the risk for machine learning models to produce more false positives. However, there are some informative features differentiating the IRGs from ISGs. For example, comparing with the ISGs, the IRGs are more enriched in CpGs (Mann-Whitney U test: p = 5.6E-03), which is also mentioned in [59]. The IRGs tend to have higher closeness centrality and neighbourhood connectivity than the ISGs (Mann-Whitney U test: p = 0.04 and 6.4E-06 respectively), suggesting that the IRGs are closer to the centre of the human PPI network and connected to key proteins with many interaction partners. Differences in some amino acid composition features between the ISGs and IRGs can also be observed in **Figure 11**. Therefore, good predictability is still expected when using features extracted from proteins sequences.

Figure 11 also illustrates 161 features showing significant differences (Mann-Whitney U tests: $p < 0.05$) in the representation of the ISGs and ELGs. An estimated 82% of these features were also differentially represented between the ISGs and non-ISGs. 79% of these significant features displayed similar over-representation or under-representation in two comparisons, i.e., ISGs versus ELGs and ISGs versus non-ISGs. These ratios indicate that the majority of the ELGs are less likely to be ISGs based on their feature profile as well as their low expression levels in cells induced with IFN-α. Network analyses showed that the ELG products tended to have lower values of all calculated network features with the exception of topological coefficient than ISG products. It means that the ELG products are less connected by other human proteins in the human PPI network. Particularly, their abnormal representation on the feature of average shortest paths indicating that some ELGs (e.g. vascular cell adhesion molecule 1 (VCAM1) and ubiquitin D (UBD)) may still have high connectivity in the human PPI network.

Figure 11. Differential expressions of parametric features between different genes and their coded proteins. Mann-Whitney U tests are applied for hypothesis testing and the results were provided in the **Supplementary Data S2**. Here, the ISGs and non-ISGs are taken from dataset S2; the IRGs and ELGs are taken from dataset S4 and dataset S8 (subsets of dataset S1); the background human genes are from dataset S1 (**Table 5**).

Implementation with machine learning framework

In this study, we encoded 397 parametric and 7046 non-parametric features for the analyses. As an excess of features will greatly increase the dimension of feature spaces and complicate the classification task for the support vector machine (SVM) [60], we limited the number of SLNPs to the top 100 based on the adjusted p-value and we expected these to be sufficient to

provide a picture of short linear sequence patterns in the coding region of the canonical transcript. Accordingly, features measuring the co-occurrence status of multiple SLNPs were recalculated based on the selected 100 SLNPs. To reduce the impact of noisy data toward classifications, we only used the refined ISGs and non-ISGs from dataset S2 in machine learning.

Measured by sensitivity (SN), specificity (SP), Matthews correlation coefficient (MCC) [61] and area under the receiver operating characteristic curve (AUC), the initial prediction results shown in **Table 3** indicate that proteome-based features, including those deciphered from protein sequences and the human interactome, perform much better than genome-based features presumably due to overfitting of the model [62]. Using parametric features that took the advantage of both genetic and proteomic aspects showed a good improvement in tests. The non-parametric features used in this study gave a binary statement for the occurrence of short linear sequence patterns in genetic and proteomic sequences but seemed not to perform well and disrupted the model when they were combined with parametric features. The results shown in the previous analyses also indicate that there are a considerable number of disruptive features hidden in the set (e.g., **Figure 4, Figure 6, and Figure 8**). The similar attributes of the ISGs and IRGs (shown in **Figure 11**) led to lots of noisy data biasing the classifiers. This situation was not ameliorated and became more difficult when using other machine learning algorithms such as k-nearest neighbors (KNN), decision tree (DT), random forest (RF) (**Table 3**) [63,64]. As some genes respond to IFNs in a cell-specific manner [2], it is hard to produce predictions unless we detect key discriminating features, which are robust to the change of biological environment.

Considering these drawbacks, we designed an AUC-driven subtractive iteration algorithm (ASI) (**Figure 15**) to remove as many disruptive features as possible (**Figure 12A**). Pre-processing using the ASI algorithm showed that there were at least 28% of features

disrupting the prediction model. They included 34% of features on codon usages and 50% of SLNP/SLAAP features, thus, explaining the poor performance of the model trained with non-parametric features (**Table 3**). However, the loss of some of the individual nucleotide 4-mer feature seemed not to influence the performance of the classifier at this stage, but the similarities between IRGs and ISGs (**Figure 11**) particularly in these 4-mer features was a cause for concern when the model was used to predict new data especially unknown IRGs.

When using the ASI algorithm, the number of disrupting features did not stabilise until the algorithm reached the 11-th iterations. The remaining 74 features constituted our optimum feature set for predicting the ISGs (**Table 4**). Among them, 14 and 9 features displayed positive and negative correlations with the level of up-regulation in IFN-α experiments. During the procedure, the AUC kept increasing steadily and reached 0.7479 at the end. The MCC also showed an overall improvement although it fluctuated slightly during the last few iterations. By degressively ranking the score calculated by the prediction model, we found 68.1% of the 496 genes (equal to the number of ISGs in the training dataset) were successfully predicted as the ISGs. **Figure 12B** illustrates the distribution of prediction scores generated by the ASI-optimised model for human genes with different expressions in IFN-α experiments. Human genes with higher up-regulation in IFN-α experiments tend to obtain higher prediction score from our optimised machine learning model (PCC = 0.243, p = 4.2E-10).

However, there were also some ISGs incorrectly predicted by our model even though they were highly up-regulated, for example, basic leucine zipper ATF-like transcription factor 2 (BATF2, prediction score = 0.34). The model produced 33 ISGs with a prediction score higher than 0.8 but this number for the non-ISGs reduced to six, including one IRG (tripartite motif containing 59 (TRIM59)). The highest prediction score within the non-ISGs was found on ubiquitin conjugating enzyme E2 R2 (UBE2R2, prediction score = 0.88). It contains many features similar to the ISGs but was not differentially expressed in the presence of IFN-α in the

human fibroblast cells [3]. The lowest prediction score within ISGs was found on cap methyltransferase 1 (CMTR1, prediction score = 0.12) due to the weak signal from its features. For instance, CMTR1 protein does not contain any ISG-favoured SLAAPs listed in **Table 1**. The influence of the IRGs on the prediction was reflected in the training dataset but was not significant. Compared with human genes not differentially expressed in the IFN-α experiments (non-ISGs but not IRGs), there were slightly more IRGs unsuccessfully classified when using a threshold of 0.549 (Pearson's chi-squared tests: M_1 = 27%, M_2 = 24%, p > 0.05).

Table 3. Performance of different feature combinations on the training dataset S2' via five-fold cross-validation.

Method	Features	Number	Threshold-dependent					Threshold-independent	
			Score range	Threshold[a]	SN	SP	MCC	SN_496[b]	AUC
SVM	Genetic	452	0.359~0.623	0.402	0.769	0.355	0.169	0.579	0.6058
SVM	Proteomic	66	0.261~0.730	0.560	0.425	0.778	0.218	0.605	0.6360
SVM	Parametric	397	0.305~0.760	0.529	0.595	0.665	0.261	0.621	0.6573
SVM	Non-parametric	121	0.368~0.605	0.487	0.653	0.504	0.159	0.573	0.5736
SVM	All	518	0.328~0.743	0.542	0.567	0.681	0.250	0.615	0.6509
KNN[c]	All	518	0.100~0.900	0.500~0.550	0.593	0.621	0.214	0.607±0.014	0.6305
DT	Partial	182[d]	0 or 1	N/A	0.546	0.548	0.095	0.546	N/A
RF[e]	Random	Random	0.080~0.900	0.380~0.579	0.590±0.168	0.617±0.183	0.219±0.019	0.600±0.007	0.6413±0.0082
SVM	Optimum	74	0.098~0.918	0.549	0.623	0.750	0.376	0.681	0.7479

a: this threshold is provided by maximum the value of MCC;

b: this sensitivity is measured among tested genes with the top 496 prediction probabilities;

c: k-value here is set as the square root of the size of the training samples in five-fold cross validation, i.e., k = 20 [65];

*d:182 out of the 518 features (**Supplementary Data S5**) are used for decisions during this modelling procedure as the rest ones are not helpful to better split the dataset for lower system entropy [66];*

e: this random forest algorithm uses 50 random grown trees and the modelling and validation procedures are repeated for 10 times.

Figure 12. The optimisation on the machine learning model with the ASI algorithm. (A) shows the change of the prediction models based on the one generated with all 518 features (disruptive feature vector = 144, best MCC = 0.250, SN_496 = 0.615, and AUC = 0.6509). (B) shows the distribution of prediction scores generated by the ASI-optimised model for human genes with different expression levels in the IFN-α system. The ISGs and non-ISGs shown in (B) are randomly selected through an undersampling strategy [67] on dataset S2. The list of gene names can be found in **Supplementary Data S1**.

Table 4. The optimum 74 features contributing to predicting the ISGs.

**Evolutionary features (2)**		
Number of human paralogues[P], average dS within human paralogues[P-].		
**Codon usage features (10)**		
Codon usage: CTA (L)[P+]	Codon usage: ATT (I)[P]	Codon usage: TAT (Y)[P]
Codon usage: GCG (A)[P-]	Codon usage: CAC (H)[P-]	Codon usage: TGC (C)[P]
Codon usage: CGT (R)[P]	Codon usage: CGA (R)[P]	Codon usage: CGG (R)[P-]
Codon usage: AGA (R)[P+]		
**Genetic composition features (40)**		
DNA AC content[P]	Dinucleotide CpT composition[P]	DNA 4-mer CGCG composition[P-]
DNA 4-mer AATC composition[P+]	DNA 4-mer TCGT composition[P]	DNA 4-mer GATG composition[P+]
DNA 4-mer AACA composition[P]	DNA 4-mer TGAG composition[P+]	DNA 4-mer GACC composition[P]
DNA 4-mer ATAT composition[P]	DNA 4-mer TGTA composition[P]	DNA 4-mer GACG composition[P]
DNA 4-mer ATGT composition[P+]	DNA 4-mer CACG composition[P]	DNA 4-mer GAGT composition[P+]
DNA 4-mer ACAC composition[P]	DNA 4-mer CTCC composition[P]	DNA 4-mer GTAC composition[P]
DNA 4-mer ACTA composition[P]	DNA 4-mer CCAC composition[P]	DNA 4-mer GTGT composition[P]
DNA 4-mer ACTC composition[P]	DNA 4-mer CCTA composition[P]	DNA 4-mer GTGC composition[P]
DNA 4-mer ACCG composition[P]	DNA 4-mer CCTC composition[P+]	DNA 4-mer GTGG composition[P]
DNA 4-mer TATG composition[P]	DNA 4-mer CCGT composition[P]	DNA 4-mer GCAA composition[P+]

DNA 4-mer TTCT composition^{P}	DNA 4-mer CGAG composition^{P}	DNA 4-mer GCTC composition^{P}
DNA 4-mer TTCG composition^{P}	DNA 4-mer CGTG composition^{P}	DNA 4-mer GCCT composition^{P}
DNA 4-mer TTGA composition^{P}	DNA 4-mer CGCA composition^{P}	DNA 4-mer GGGG composition^{P}
DNA 4-mer TCAT composition^{P}		
**Proteomic composition features (9)**		
Arginine composition^{P}, cysteine composition^{P+}, methionine composition^{P};		
Basic amino acid composition (R/H/K)$^{P+}$	Sulfur amino acid composition (C&M)$^{P+}$	
Hydroxyl amino acid composition (S&T)$^{P-}$	Small amino acid composition (N/D/C/P/T)$^{P-}$	
Large amino acid composition (R/I/L/K/M)$^{P+}$		
Uncharged amino acid composition (A/N/C/Q/G/I/L/M/F/P/S/T/W/Y/V)$^{P-}$		
Features about human interactome network (3)		
Average shortest paths^{P+}, betweenness^{P}, neighborhood connectivity^{P-}.		
**Sequence pattern features (8)**		
SLNP: ATA[AG][TG]^{N}	SLNP: TAT[AT]T^{N}	SLNP: T[AT]AAA^{N}
SLNP: [ATG]TGTA^{N}	SLAAP: SxNxE^{N}	SLAAP: ENE^{N}
SLAAP: SVI^{N}	Co-occurrence of SLAAPs^{P}	

P: parametric features;

N: non-parametric features;

'+' symbol means features are positively associated with the level of up-regulation in IFN-α experiments ($p < 0.05$);

'-' symbol means features are negatively associated with the level of up-regulation in IFN-α experiments ($p < 0.05$).

Review of different testing datasets

In this study, we trained and optimised a SVM model from our training dataset S2', and prepared seven testing datasets (dataset S2''/S3/S4/S5/S6/S7/S8) to assess the generalisation capability of our model under different conditions (**Table 5**). The S2'' testing dataset was a subset of dataset S2. The prediction performance on this testing dataset was close to that in the training stage with an AUC of 0.7455 (**Figure 13A**). The best MCC value (0.345) was achieved

when setting the judgement threshold to 0.438, which meant that the prediction model was sensitive to signals related to ISGs. In this case, it performed predictions with high sensitivity but inevitably produced many false positives, especially within IRGs.

In the S3 testing dataset, we used 695 ISGs with low confidence. The overall accuracy (equals to SN as there were no negatives) only reached 44.0% when using a judgement threshold of 0.549, about 0.18 lower than SN under the same threshold in the training dataset S2' (**Table 3**). It is expected as some of their inherent attributes make them slightly up-regulated, silent or even repressed (e.g., become non-ISGs in other IFN systems) in response to some IFN-triggered signalling. On this testing dataset, our machine learning model produced 38 (5.5%) ISGs with a prediction score higher than 0.8. This number was also lower than that on the training dataset S2'. It further indicates the relatively low confidence for the ISGs included in dataset S3.

The S4 testing dataset was constructed to illustrate our hypothesis that there are some patterns shared among the ISGs and IRGs at least in the IFN-α system in the human fibroblast cells. On this testing dataset, the prediction accuracy (equals to SP as there were no positives) was 60.2% under the judgement threshold of 0.549, about 0.15 lower than the SP under the same threshold in the training dataset S2' (**Table 3**). Leucine rich repeat containing 2 (LRRC2), carbohydrate sulfotransferase 10 (CHST10) and eukaryotic translation elongation factor 1 epsilon 1 (EEF1E1) showed strong signals of being ISGs (probability score > 0.9). In total, there were 56 (5.6%) IRGs being incorrectly predicted as the ISGs with prediction scores higher than 0.8. This high score was found in an estimated 8.1% of the ISGs but was only observed in 1.2% of human genes not differentially expressed in the IFN-α experiments (**Figure 12B**). These results indicate that there is a considerable number of IRGs incorrectly predicted as ISGs in the S4 testing dataset due to their close distance to the ISGs in the high-dimensional feature space. This may be the case for many other datasets including dataset S2'',

S5, S6, S7, and S8. It also supports our hypothesis about the shared patterns from the machine learning aspect and is consistent with the results shown in **Figure 11**.

The next three testing datasets (S5, S6, and S7) were collected from the Interferome database [24] to test the applicability of the machine learning model across different IFN types. The ISGs in these testing datasets were all highly up-regulated (Log_2(Fold Change) > 1.0) in the corresponding IFN systems while all the non-ISGs were not up-regulated after corresponding IFN treatments (Log_2(Fold Change) < 0). The results shown in **Figure 13** reveals that the ISGs triggered by type I or III IFN signalling can still be predicted by our machine learning model, but the performance is limited to some extents (AUC = 0.6677 and 0.6754 respectively). However, it is almost impossible to make normal predictions with the current feature space for human genes up-regulated by type II IFNs (AUC = 0.5532).

Figure 13. The performance of our optimised model on different datasets. (A) and (B) illustrate the AUC and best MCC. S2' is the training dataset used in this study. It randomly includes 496 ISGs and an equal number of non-ISGs from dataset S2 that contains ISGs/non-ISGs with high confidence (**Table 5**). Evaluation on this dataset in (A) is processed via five-fold cross validation. S2'' is the testing dataset constructed with the remaining human genes in dataset S2. S5, S6, and S7 are collected from the Interferome database [24], including human genes with different responses to the type I, II and III IFNs, respectively. The label and usage of these human genes are provided in **Supplementary Data S1**.

The S8 testing dataset consisted of 2217 human genes that were insufficiently expressed in IFN-α experiments in the human fibroblast cells [3]. The results showed that there were around 41.2% ELGs being predicted as the ISGs when using a judgement threshold of 0.549. This was approximately 0.21 lower than the SN under the same threshold in the training dataset

S2' (**Table 3**). It suggests that there are more non-ISGs than ISGs in this dataset, which is consistent with the results shown in **Figure 11**. Particularly, we found ten ELGs with prediction scores higher than 0.9: CD48 molecule, CD53 molecule, lipocalin 2 (LCN2), uncoupling protein 1 (UCP1), coiled-coil domain containing 68 (CCDC68), potassium calcium-activated channel subfamily M regulatory beta subunit 2 (KCNMB2), potassium voltage-gated channel interacting protein 4 (KCNIP4), zinc finger HIT-type containing 3 (ZNHIT3), serpin family B member 4 (SERPINB4), and fibrinogen silencer binding protein (FSBP). By retrieving data from the Genotype-Tissue Expression project [68], we found that the expression of these ELGs were generally limited with the exception of CD53 and ZNHIT3 (**Figure 14**). The expression data of CD53 were not included in the OCISG database [3] and were also limited in the Interferome database [24]. It only showed slight up-regulation after type I IFN treatments in blood, liver, and brain but there is currently no record of its expression level in the presence of IFN-α in the human fibroblast cells. ZNHIT3 is another well-expressed gene lacking information in the OCISG. In the Interferome database [24], we found that ZNHIT3 could be up-regulated after IFN treatments in some fibroblast cells on skin. As for the remaining eight ELGs, despite their limited expression in the human fibroblast cells, their features suggest that they are very likely to be IFN-α stimulated in a currently untested cell type.

Figure 14. Expression of the ELGs in different tissues. Expression data for ten ELGs are collected from the Genotype-Tissue Expression project (https://gtexportal.org/) [68]. The tissues in red are not included in the Interferome database [24]. White boxes in the heatmap indicate that there is no data available for genes in the corresponding tissues. The overall expression level of these ten ELGs are reflected via human perspective photo retrieved from Expression Atlas (https://www.ebi.ac.uk/gxa) [69].

Discussion

In this study, we investigated the characteristics that influence the expression of human genes in IFN-α experiments. We compared the ISGs and non-ISGs through multiple procedures to guarantee strong signals for the ISGs and to avoid cell-specific influences that resulted in the lack of the ISGs expression in certain cell types [2]. Even some highly up-regulated ISGs can become down-regulated when the biological conditions change, exemplified by the performance of C-X-C motif chemokine ligand 10 (CXCL10) on liver biopsies after IFN-α treatment. This refinement is necessary as the representation of features between the ISGs and background human genes show that many non-ISGs especially IRGs have similar feature patterns to the ISGs (**Figure 11**).

Generally, the ISGs are less evolutionarily conserved with more human paralogues than the non-ISGs. They have specific nucleotide patterns exemplified by the depletion of GC-content and have a unique codon usage preference in coding proteins. There are a number of SLNPs widely observed in the cDNA of the ISGs which are relatively rare in the non-ISGs (**Supplementary Data S4**). Likewise, there are also many SLAAPs highlighted in the sequences of ISG products that are absent or rare in the non-ISG products (**Table 1**). In the human PPI network, the ISG products tend to have higher betweenness than the background human protein, indicating their more frequent interruption of the shortest path (geodesic distance) between different nodes. Abnormal expression or knockout of these proteins will increase the diameter of the network and may lead to some lethal consequences that are not tolerated in signalling pathways [70-72]. These ISG specific patterns may be the result of the evolution of the innate immune system in vertebrates and could be adaptations to the cellular environment induced by interferon following a pathogenic infection [73]. It is also possible that some of the particular SLNPs and SLAAPs may be functionally important as the cell

changes from non-infected to infected. Experimental evidence will be necessary to investigate this.

Some inherent properties of the ISGs facilitate or elevate their expression after IFN-α treatments but may also be used by viruses to escape from IFN-α-mediated antiviral response [22]. For instance, the representation of dN showed a more significant difference than that of dS within human paralogues. We found that higher dN/dS ratio was positively correlated with gene up-regulation following IFN-α treatments (**Figure 9**). It means the gene is less conserved with more non-synonymous or nonsense mutations, which can often be associated to inherited diseases and cancer [74]. It will also facilitate the virus to interfere with IFN-α signalling through the JAK-STAT pathway and inactivate downstream cellular factors involved in IFN-α signal transductions [22]. We found arginine was under-represented in the ISG products compared to the non-ISG products. As arginine is essential for the normal proliferation and maturation of human T cells [75], such depletion in the ISG products may leave a risk of inhibiting T- cell function and potentially increased susceptibility to infections [76]. Furthermore, the special pattern of the ISGs also promotes the representation of some features even if they are not well represented in nature, for example, the higher cysteine composition in the ISGs. We hypothesize that it may be helpful to activate T-cell to regulate protein synthesis, proliferation and secretion of immunoregulatory cytokines [77,78]. There are also some features (e.g. methionine composition) not differentially represented between the ISGs and non-ISGs but play important roles in IFN-α-mediated immune responses. For example, there is evidence for the methionine content playing a role in the biosynthesis of S-Adenosylmethionine (SAM), which can improve interferon signalling in cell culture [79,80].

As previously mentioned, there were similar patterns between the feature representation of the ISGs and IRGs, which led to the unclear boundary for the ISGs and non-ISGs in the feature space. We found significant differences on the representation of features on

evolutionary conservation (**Figure 3**) between the ISGs and non-ISGs, but they became non-significant when comparing the ISGs with IRGs. Similar phenomena were observed on many features deciphered from the canonical transcript, e.g., dinucleotide composition and codon usage features. We suggest that the IRGs can be viewed as additional ISGs as they also regulate the activity of human genes in response to IFNs, only negatively. Furthermore, despite so many similarities between the ISGs and IRGs, the separate classification of these genes is still possible. 4-mer compositions can be considered as the key features as most of them are differentially represented between ISGs and IRGs (**Figure 11**). Using proteomic features can also help to differentiate the ISGs from IRGs but is not as good as using 4-mer features.

In the machine learning framework, we developed the ASI algorithm to remove disruptive features but kept features not influencing the prediction performance when being removed individually during iterations. Features might have synergistic effects thus the elimination of each feature left a different impact on the remaining ones even if these were individually useless for the improvement of the classifier. In this case, keeping as many useful features as possible seems to be a good option but will greatly increase the dimension of the feature space and increase the risk of overfitting [62]. By contrast, our ASI algorithm avoided such a risk and kept the synergistic effect of different features through iterations.

In the prediction task, we found some previously labelled non-ISGs with very high prediction scores, suggesting that they had many inherent properties enabling them to be stimulated after IFN-α treatments. Some of them, for example, UBE2R2 has been shown to be significantly up-regulated after IFN-α treatment [81]. The non-ISG label was assigned because the relevant expression data in the presence of IFN-α were not included in the OCISG [3] and Interferome databases [24]. We also found ten ELGs with very high prediction scores (> 0.9). Literature searches on these genes indicate that they are likely to be involved in the innate immune response [82,83]. Their responses may be limited to certain tissues or cell types for

which there is limited expression data in the Interferome database [24]. For example, LCN2 has been shown to mediate an innate immune response to bacterial infections by sequestering iron [82] and is induced in the central nervous system of mice infected with West Nile virus encephalitis [84]. CD48 was shown to increase in levels in the context of human IFN-α/β/γ stimulation [83]. Interestingly, CD48 is also the target of immune evasion by viruses [85] and has been captured in the genome of cytomegalovirus and undergone duplication [86]. Evidence for other ELGs is harder to assess, particularly those for which expression is absent in a range of tissues (e.g., UCP1 in **Figure 14**). UCP1 is a mitochondrial carrier protein expressed in brown adipose tissue (BAT) responsible for non-shivering thermogenesis [87]. It is possible that UCP1 is stimulated directly or indirectly by IFN-α in BAT, resulting in the defended elevation of body temperature in response to infection.

We developed the machine learning model based on experimental data from the human fibroblast cells stimulated by IFN-α. It can be generalised to type I or III IFN systems, presumably because activations of type I and III ISGs are both controlled by ISRE [9] and aim to regulate host immune response [10-12]. However, our model cannot be used for predictions in the type II IFN system (AUC = 0.5532, best MCC = 0.083, **Figure 13**) because of the different control element and the different role in human immune activities [14].

In summary, our analyses highlight some key sequence-based features that are helpful to distinguish the ISGs from non-ISGs or IRGs. Our machine learning model is able to produce a list of putative ISGs to support IFN-related research. As knowledge of the ISG functions continue to be elucidated by experimentalists, the *in-silico* approach applied here can in future be extended to classify the different functions of ISGs.

Methods

Dataset curation

In this study, we retrieved 2054 ISGs (up-regulated), 12379 non-ISGs (down-regulated or not differentially expressed), and 3944 unlabelled human genes (ELGs with less than one count per million reads mapping across the three biological replicates [88,89]) from the OCISG database (http://isg.data.cvr.ac.uk/) [3]. Gene clusters in the OCISG database were built through Ensembl Compara [90], which provided a thorough account of gene orthology based on whole genomes available in Ensembl [58]. Labels of these human genes were defined based on the fold change and a false discovery rate (FDR) following the IFN-α treatments in the human fibroblast cells. We searched the collected 18377 entries against the RefSeq database (https://www.ncbi.nlm.nih.gov/refseq/) [32] to decipher features based on appropriate transcripts (canonical) [91] coding for the main functional isoforms of these human genes. It produced 1315, 7304, and 2217 results for the ISGs, non-ISGs and ELGs, respectively. These 10836 human genes were well-annotated by multiple online databases and were used as the background dataset S1 in the analyses.

For the purpose of generating a set of human genes with high confidence of being up-regulated and non-up-regulated in response to the IFN-α, we searched the recompiled 8619 human genes (ISGs or non-ISGs) against Interferome (http://www.interferome.org/) [24]. We filtered out the ISGs without high up-regulation (Log_2(Fold Change) > 1.0) or with obvious down-regulation (Log_2(Fold Change) < -1.0) in the presence of type I IFNs. This procedure guaranteed a refined ISG dataset with strong levels of stimulation induced by any type I IFNs and reduced biases driven by the IRGs for the analyses and predictions. We filtered out the non-ISGs showing enhanced expression after type I IFN treatments (Log_2(Fold Change) > 0). The exclusion of these non-ISGs could effectively reduce the risk of involving false negatives

in analyses and producing false positives in predictions. As a result, the refined dataset S2 contains 620 ISGs and 874 non-ISGs with relatively high confidence.

The training procedure in the machine learning framework was conducted on the balanced dataset S2'. It consisted of 992 randomly selected ISGs and non-ISGs from dataset S2. The remaining human genes in S2 were used for independent testing. Additionally, we also constructed another six testing datasets for the purpose of review and assessment. Dataset S3 contained 695 ISGs with low confidence compared to those ISGs in dataset S2. Some of them could be non-ISGs or even IRGs in the type I IFN system. Dataset S4 contained 1006 IRGs from the human fibroblast cell experiments. Dataset S5, S6, and S7 were constructed based on records for experiments in type I, II, and III IFN systems from Interferome [24]. The criterion for an ISG in the latter three datasets was a high level of up-regulation (Log_2(Fold Change) > 1.0) while that for non-ISGs was no up-regulation after IFN treatments (Log_2(Fold Change) < 0). The last testing dataset S8 was derived from our background dataset S1, containing 2217 ELGs. A breakdown of the aforementioned eight datasets is shown in **Table 5**. Detailed information of the human genes used in this study is provided in **Supplementary Data S1**. The cDNA and protein sequences are accessible at http://isgpre.cvr.gla.ac.uk/.

Table 5. A breakdown of datasets used in this study.

Dataset	Brief description	IFN system	ISGs	Non-ISGs	ELGs
S1	Well-annotated human genes (background)	IFN-α in fibroblast cells	1315	7304	2217
S2	Refined dataset with high confidence	IFN-α in fibroblast cells	620	874	0
S2'	Training subset of S2	IFN-α in fibroblast cells	496	496	0
S2"	Testing subset of S2	IFN-α in fibroblast cells	124	378	0
S3	ISGs with low confidence in S1	IFN-α in fibroblast cells	695	0	0
S4	IRGs divided from S1	IFN-α in fibroblast cells	0	1006	0
S5	ISGs from Interferome [24]	Type I IFNs in all cells	1259	872	0

S6	ISGs from Interferome [24]	Type II IFN in all cells	2229	755	0
S7	ISGs from Interferome [24]	Type III IFN in all cells	33	1683	0
S8	ELGs divided from S1	IFN-α in fibroblast cells	0	0	2217

Generation of parametric features

We encoded 397 parametric features from aspects of evolution, nucleotide composition, transcription, amino acid composition, and network preference. Original values of these features for our compiled 10836 human genes are accessible at http://isgpre.cvr.gla.ac.uk/.

From the perspective of evolution, we used the number of transcripts, open reading frames (ORFs) and count of exons used for coding to quantify the alternative splicing process. Genes with more transcripts and ORFs have higher alternative splicing diversity to produce proteins with similar or different biological functions [33,92,93]. Frequent use of protein-coding exons indicates more complex alternative splicing products [94]. Here, duplication and mutation features were measured by the number of within species paralogues and substitutions [34,35]. These data were collected from BioMart [58] to assess the selection on protein sequences and mutational processes affecting the human genome [95].

From the perspective of nucleotide composition, we calculated the percent of adenine, thymine, cytosine, guanine, and their four-category combinations in the coding region of the canonical transcript. The first category measured the proportion of two different nitrogenous bases out of the implied four bases, e.g., GC-content. The second category also focused on the combination of two nucleotides but added the impact of phosphodiester bonds along the 5' to 3' direction, e.g., CpG-content [96]. The third category calculated the occurrence frequency of 4-mers, e.g., 'CGCG' composition to involve some positional resolution [41]. The last category considered the co-occurrence of SLNPs. From the perspective of transcription, we calculated the usage of 61 coding codons and three stop codons in the coding region of the canonical transcripts. Codon usage biases are observed when there are multiple codons available for

coding one specific amino acid. They can affect the dynamics of translation thus regulate the efficiency of translation and even the folding of the proteins [40,97].

From the perspective of amino acid composition, we calculated the percentage of 20 standard amino acids and their combinations based on their physicochemical properties [46]. Patterns in the amino acid level are considered to have a direct impact on the establishment of biological functions or to reflect the result of strong purifying selection [47]. Based on the chemical properties of the side chain, we grouped amino acids into seven classes including aliphatic, aromatic, sulfur, hydroxyl, acidic, amide, and basic amino acids. We also grouped amino acids based on geometric volume, hydropathy, charge status, and polarity, but found some overlaps among these features. For instance, amino acids with basic side chains are all positively charged. Aromatic amino acids all have large geometric volumes (volume > 180 cubic angstroms). Likewise, we also considered the co-occurrence of short linear sequence patterns at the protein level. These co-occurring SLAAPs may relate to potential mechanisms regulating the expression of the ISGs [98].

When trying to measure the network preference for the gene products, we constructed a human PPI network based on 332,698 experimentally verified interactions (confidence score > 0.63) from HIPPIE [55]. Nodes and edges of this network are provided at http://isgpre.cvr.gla.ac.uk/. Eight network-based features including the average shortest path, closeness, betweenness, stress, degree, neighbourhood connectivity, clustering coefficient, and topological coefficient were calculated from this network. Isolated nodes or proteins were not included in our network and were assigned zero value for all these eight features. The shortest path measures the average length of the shortest path between a focused node and others in the network. Closeness of a node is defined as the reciprocal of the length of the average shortest path. Proteins with a low value of the shortest paths or closeness are close to the centre of the network. Betweenness reflects the degree of control that one node exerted over the interactions

of other nodes in the network [99]. Stress of a node measures the number of shortest paths passing through it. Proteins with a high value of betweenness or stress are close to the bottleneck of the network. Degree of a node counts the number of edges linked to it while neighbourhood connectivity reflected the average degree of its neighbours. Proteins with high degree or neighbourhood connectivity are close to the hub of the network. They are considered to play an important role in the establishment of the stable structure of the human interactome [100]. Clustering and topological coefficient measure the possibility of a node to form clusters or topological structures with shared neighbours. The former coefficient can be used to identify the modular organisation of metabolic networks [101] while the latter one may be helpful to find out virus mimicry targets [53].

Generation of non-parametric features

In this study, non-parametric features were used to check the occurrence of short linear sequence patterns in the genome and proteome. SLNPs constructed in this study contained three to five random nucleotides, producing 708,540 alternative choices. SLNPs with no restrictions on their first or last position were not taken into consideration as their patterns could be expressed in a more concise way. A SLNP was picked out to encode a binary feature when its occurrence level in the coding region of the canonical ISG transcripts was significantly higher than that for the non-ISGs (Pearson's chi-squared test: $p < 0.05$). SLAAPs were constructed with three to four fixed amino acids separated by putative gaps. The gap could be occupied by at most one random amino acid, producing 1,312,000 alternative choices. Likewise, binary features were prepared for SLAAPs showing significant enrichment in the ISG products than in the non-ISG products (Pearson's chi-squared test: $P < 0.05$). Since there were lots of results rejecting the null-hypothesis, we adopted the Benjamini-Hochberg correction procedure to avoid type I error [43]. Additionally, we also encoded two features to

check the co-occurrence or absence of multiple SLNPs and SLAAPs. This co-occurrence status might be a better representation of functional sites composed of short stretches of adjacent nucleobases or amino acids surrounding SLNPs or SLAAPs [47].

Assessment of associations between feature representation and IFN-triggered stimulations

We obtained 8619 human genes with expression data from the OCISG database [3]. 4111 of them were annotated with a positive Log_2(Fold Change) ranging from 0 to 12.6, which meant they were up-regulated after IFN-α treatments in the human fibroblast cells. In order to measure the average level of feature representation (AREP) for genes with similar expression during IFN stimulations, we introduced a 0.1-length sliding-window to divide the data into 126 bins with different Log_2(Fold Change). Here, PCC was introduced to test the association between the representation of parametric features and IFN-α-triggered stimulation (Log_2(Fold Change) > 0). It can be formulated as:

$$PCC(f) = \frac{1}{n-1}\sum_{i=1}^{n}\left(\frac{LFC_i - M_0}{SD_0}\right) \times \left(\frac{AREP_i - M_f}{SD_f}\right) \quad \mathbf{(1)}$$

where n is the number of divided parts that equals to 126 in this study; LFC_i and $AREP_i$ are the value of Log_2(Fold Change) and AREP in the i-th part; M_0 and SD_0 are the mean and standard deviation of Log_2(Fold Change), which is set as 6.4 and 3.7 respectively in this study; M_f and SD_f are the mean and standard deviation of 126 AREP that reflect the representation of the considered feature. To make fair comparisons among features with different scales, we normalised them based on the major value of their representations:

$$Norm(f) = \begin{cases} 1, f > UB(f) \\ \frac{f - LB(f)}{UB(f) - LB(f)}, LB(f) < f < UB(f) \\ 0, f < LB(f) \end{cases} \quad \mathbf{(2)}$$

where $LB(f)$ and $UB(f)$ are the lower and upper bound representing the 5th and 95th percentile within representation values for the target feature. The representation of feature was considered to have a stronger positive/negative association with IFN-α-triggered stimulations if the PCC calculated from the normalised features was closer to 1.0/-1.0 and the p value calculated by the Student t-test was lower than 0.05.

Machine learning and optimisation

We designed a machine learning framework for the prediction of ISGs. Firstly, all features were encoded and normalised based on their major representations (**Equation 2**). Then we used an under-sampling procedure [67] to generate a balanced dataset from dataset S2 for training and modelling. SVM with radial basis function [60] was used as the basic classifier. It maps the normalised feature space to a higher dimension to generate a space plane to better classify the majority of positive and negative samples. Since there were usually lots of noisy data distributed in the feature space, it was necessary to remove disruptive features. This effectively reduced the dimensionality of the feature space and made it easier for the SVM model to generate a more appropriate classification plane that involved fewer false positives and false negatives. Here, we propose a subtractive iteration algorithm driven by the change of AUC to filter out disruptive features (**Figure 15**). In each iteration, we traversed the features and removed those that do not improve the AUC of the prediction results. Theoretically, this algorithm can greatly optimise the feature space and remove all disruptive features after multiple iterations. In the testing procedure, we encoded the optimum features for testing samples and place them in the optimised feature space. Samples with longer distance to the optimised classification plane indicated a stronger signal of being the ISGs or non-ISGs. They were more likely to get higher prediction scores (close to 0 or 1) from the SVM model.

Figure 15. The pseudo-code of the AUC-driven subtractive iteration algorithm.

Performance evaluation

In this study, the prediction results were evaluated with three threshold-dependent criteria including SN, SP, and MCC [61] and two threshold-independent criteria: SN_n and AUC. SN and SP were used to assess the quality of the machine learning model in recognising ISGs and non-ISGs respectively while MCC provided a comprehensive evaluation for both positives and negatives. The number of 'n' in the SN_n criterion was determined based on the number of ISGs used for testing. It was used to measure the upper limit of the prediction model as well as to check the existence of important false positives close to the class of ISGs from the perspective of data expression. Finally, AUC was a widely used criterion to evaluate the prediction ability of a binary classifier system. The group of interest was almost unpredictable in a specific binary classifier system if the AUC of the classifier was close to 0.5.

Availability of source code and requirements

- Project name: ISGPRE
- Project home page: http://isgpre.cvr.gla.ac.uk/
- Operating system: mac OS
- Programming language: Java
- Other requirements: JDK 8+
- License: GNU GPL v3
- Any restrictions to use by non-academics: None
- Documentation and tutorials: https://github.com/HChai01/ISGPRE

Additionally, we have released all of our compiled data and calculated features at the project home page and GitHub repository. They can be reused to conduct research relating to IFN-α or type I/II/III IFNs.

Data Availability

The implemented web server and all reproduceable data are freely accessible at http://isgpre.cvr.gla.ac.uk/ and https://github.com/HChai01/ISGPRE.

Additional Files

Supplementary Data S1. Basic information and usage of our compiled 10836 human genes.

Supplementary Data S2. The result of Mann-Whitney U tests for parametric features.

Supplementary Data S3. Association between feature representations and IFN-α stimulations.

Supplementary Data S4. The result of Pearson's chi-squared tests for sequence motifs.

Supplementary Data S5. Decision trees generated during five-cross validation on the training dataset S2'.

Abbreviations

APC: anaphase promoting complex; AREP: average level of feature representation; ASI: AUC-driven subtractive iteration algorithm; AUC: area under the receiver operating characteristic curve; BAT: brown adipose tissue; BATF2: basic leucine zipper ATF-like

transcription factor 2; BST2: bone marrow stromal cell antigen 2; CCDC68: coiled-coil domain containing 68; cDNA: complementary DNA; CHST10: carbohydrate sulfotransferase 10; CMTR1: cap methyltransferase 1; CXCL10: C-X-C motif chemokine ligand 10; dN: non-synonymous substitutions per non-synonymous site; dS: synonymous substitutions per synonymous site; DSP: desmoplakin; DT: decision tree; EEF1E1: eukaryotic translation elongation factor 1 epsilon 1; ELAVL1: embryonic lethal, abnormal vision like RNA binding protein 1; ELGs: human genes with limited expression in the IFN-α experiments; ESR2: estrogen receptor 2; FDR: false discovery rate; FSBP: fibrinogen silencer binding protein; GAF: IFN-γ activation factor; GAS: gamma-activated sequence promoter elements; gBGC: GC-biased gene conversion; HIPPIE: Human Integrated Protein-Protein Interaction rEference; HMCN1: hemicentin 1; HPSE: ectopic expression of heparinase; IDRs: intrinsically disordered regions; IFITM: interferon induced transmembrane proteins; IFNAR: interferon-α receptor; IFNGR: IFN-γ receptor; IFNLR1: IFN-λ receptor 1; IFNs: interferons; IL-10R2: interleukin-10 receptor 2; IRF9: interferon regulatory factor 9; IRG: interferon repressed (down-regulated) human genes; ISG15: ISG15 ubiquitin like modifier; ISG20: interferon stimulated exonuclease gene 20; ISGF3: interferon stimulated gene factor 3 complex; ISGs: interferon stimulated (up-regulated) human genes; ISRE: interferon stimulated response elements; JAK1: Janus kinase 1; KCNIP4: potassium voltage-gated channel interacting protein 4; KCNMB2: potassium calcium-activated channel subfamily M regulatory beta subunit 2; KNN: k-nearest neighbors; LCN2: lipocalin 2; LRRC2: Leucine rich repeat containing 2; MCC: Matthews correlation coefficient; MX: MX dynamin like GTPase proteins; non-ISGs, human genes not significantly up-regulated by interferons; NTRK1: neurotrophic receptor tyrosine kinase 1; OCISG: Orthologous Clusters of Interferon-stimulated Genes; ORF: open reading frame; PCC: Pearson's correlation coefficient; PPI: protein-protein interaction; RefSeq: Reference Sequence; RF: random forest; SAM: S-Adenosylmethionine; SERPINB4: serpin family B

member 4; SLAAP: short linear amino acid pattern; SLNP: short linear nucleotide pattern; SN: sensitivity; SP: specificity; STAT: signal transducer and activator of transcription; SVM: support vector machine; TDRD6: tudor domain containing 6; TRIM25: tripartite motif containing 25; TRIM5: tripartite motif containing 5; TRIM59: tripartite motif containing 59; TYK2: tyrosine kinase 2; UBD: ubiquitin D; UBE2R2: ubiquitin conjugating enzyme E2 R2; UCP1: uncoupling protein 1; VCAM1: vascular cell adhesion molecule 1; ZNHIT3: zinc finger HIT-type containing 3.

Competing Interests

The authors have declared that no competing interests exist.

Funding

HC is supported by the China Scholarship Council (201706620069). JH, QG and DLR are supported by the Medical Research Council (MC_UU_1201412). The funders had no role in study design, data collection and analysis, decision to publish, or preparation of the manuscript.

Authors’ Contributions

Conceptualization: all authors; data curation: H. C.; formal analysis: H. C.; funding acquisition: D. L. R.; investigation: H. C.; methodology: H. C.; project administration: D. L. R., J. H.; resources: Q. G., J. H., D. L. R.; web server: H. C.; supervision: Q. G., J. H., D. L. R.; validation: all authors; visualization: H. C.; writing original draft: H. C.; writing review & editing: all authors.

Acknowledgments

The authors wish to thank Drs Andrew Davison, Suzannah Rihn and Sam Wilson for helpful discussions and recommendations, and Scott Arkison for help setting up the website.

References

1. Rönnblom L. The type I interferon system in the etiopathogenesis of autoimmune diseases. Ups J Med Sci. 2011;116(4):227-37.
2. Mostafavi S, Yoshida H, Moodley D, LeBoité H, Rothamel K, Raj T, et al. Parsing the interferon transcriptional network and its disease associations. Cell. 2016;164(3):564-78.
3. Shaw AE, Hughes J, Gu Q, Behdenna A, Singer JB, Dennis T, et al. Fundamental properties of the mammalian innate immune system revealed by multispecies comparison of type I interferon responses. PLoS Biol. 2017;15(12):e2004086.
4. Shalhoub S. Interferon beta-1b for COVID-19. The Lancet. 2020;395(10238):1670-1.
5. Harris BD, Schreiter J, Chevrier M, Jordan JL and Walter MR. Human interferon-ϵ and interferon-κ exhibit low potency and low affinity for cell-surface IFNAR and the poxvirus antagonist B18R. J Biol Chem. 2018;293(41):16057-68.
6. Li S-f, Zhao F-r, Shao J-j, Xie Y-l, Chang H-y and Zhang Y-g. Interferon-omega: Current status in clinical applications. Int Immunopharmacol. 2017;52):253-60.
7. Kak G, Raza M and Tiwari BK. Interferon-gamma (IFN-γ): exploring its implications in infectious diseases. Biomol Concepts. 2018;9(1):64-79.

8. Hemann EA, Gale Jr M and Savan R. Interferon lambda genetics and biology in regulation of viral control. Front Immunol. 2017;8):1707.

9. Schneider WM, Chevillotte MD and Rice CM. Interferon-stimulated genes: a complex web of host defenses. Annu Rev Immunol. 2014;32):513-45.

10. Kotenko SV and Durbin JE. Contribution of type III interferons to antiviral immunity: location, location, location. J Biol Chem. 2017;292(18):7295-303.

11. Fensterl V and Sen GC. Interferons and viral infections. Biofactors. 2009;35(1):14-20.

12. Lazear HM, Schoggins JW and Diamond MS. Shared and distinct functions of type I and type III interferons. Immunity. 2019;50(4):907-23.

13. Takaoka A and Yanai H. Interferon signalling network in innate defence. Cell Microbiol. 2006;8(6):907-22.

14. Stark GR and Darnell Jr JE. The JAK-STAT pathway at twenty. Immunity. 2012;36(4):503-14.

15. Schoggins JW. Interferon-stimulated genes: what do they all do? Annu Rev Virol. 2019;6):567-84.

16. Aso H, Ito J, Koyanagi Y and Sato K. Comparative description of the expression profile of interferon-stimulated genes in multiple cell lineages targeted by HIV-1 infection. Front Microbiol. 2019;10):429.

17. Dang W, Xu L, Yin Y, Chen S, Wang W, Hakim MS, et al. IRF-1, RIG-I and MDA5 display potent antiviral activities against norovirus coordinately induced by different types of interferons. Antiviral Res. 2018;155):48-59.

18. Masola V, Bellin G, Gambaro G and Onisto M. Heparanase: A multitasking protein involved in extracellular matrix (ECM) remodeling and intracellular events. Cells. 2018;7(12):236.

19. Schoggins JW. Recent advances in antiviral interferon-stimulated gene biology. F1000Research. 2018;7

20. Spence JS, He R, Hoffmann H-H, Das T, Thinon E, Rice CM, et al. IFITM3 directly engages and shuttles incoming virus particles to lysosomes. Nat Chem Biol. 2019;15(3):259-68.

21. Haller O, Staeheli P, Schwemmle M and Kochs G. Mx GTPases: dynamin-like antiviral machines of innate immunity. Trends Microbiol. 2015;23(3):154-63.

22. García-Sastre A. Ten strategies of interferon evasion by viruses. Cell Host Microbe. 2017;22(2):176-84.

23. Giotis ES, Robey RC, Skinner NG, Tomlinson CD, Goodbourn S and Skinner MA. Chicken interferome: avian interferon-stimulated genes identified by microarray and RNA-seq of primary chick embryo fibroblasts treated with a chicken type I interferon (IFN-α). Vet Res. 2016;47(1):1-12.

24. Rusinova I, Forster S, Yu S, Kannan A, Masse M, Cumming H, et al. Interferome v2. 0: an updated database of annotated interferon-regulated genes. Nucleic Acids Res. 2012;41(D1):D1040-D6.

25. OhAinle M, Helms L, Vermeire J, Roesch F, Humes D, Basom R, et al. A virus-packageable CRISPR screen identifies host factors mediating interferon inhibition of HIV. Elife. 2018;7):e39823.

26. Zhang Y, Burke CW, Ryman KD and Klimstra WB. Identification and characterization of interferon-induced proteins that inhibit alphavirus replication. J Virol. 2007;81(20):11246-55.

27. Stark R, Grzelak M and Hadfield J. RNA sequencing: the teenage years. Nature Reviews Genetics. 2019;20(11):631-56.

28. Pamela C, Kanchwala M, Liang H, Kumar A, Wang L-F, Xing C, et al. The IFN response in bats displays distinctive IFN-stimulated gene expression kinetics with atypical RNASEL induction. The Journal of Immunology. 2018;200(1):209-17.

29. Feld JJ, Nanda S, Huang Y, Chen W, Cam M, Pusek SN, et al. Hepatic gene expression during treatment with peginterferon and ribavirin: Identifying molecular pathways for treatment response. Hepatology. 2007;46(5):1548-63.

30. Dalman MR, Deeter A, Nimishakavi G and Duan Z-H. Fold change and p-value cutoffs significantly alter microarray interpretations. In: *BMC Bioinformatics* 2012, pp.1-4. BioMed Central.

31. Trilling M, Bellora N, Rutkowski AJ, de Graaf M, Dickinson P, Robertson K, et al. Deciphering the modulation of gene expression by type I and II interferons combining 4sU-tagging, translational arrest and in silico promoter analysis. Nucleic Acids Res. 2013;41(17):8107-25.

32. O'Leary NA, Wright MW, Brister JR, Ciufo S, Haddad D, McVeigh R, et al. Reference sequence (RefSeq) database at NCBI: current status, taxonomic expansion, and functional annotation. Nucleic Acids Res. 2016;44(D1):D733-D45.

33. Bragg JG, Potter S, Bi K and Moritz C. Exon capture phylogenomics: efficacy across scales of divergence. Mol Ecol Resour. 2016;16(5):1059-68.

34. Kondrashov FA, Rogozin IB, Wolf YI and Koonin EV. Selection in the evolution of gene duplications. Genome Biol. 2002;3(2):1-9.

35. Esposito M and Moreno-Hagelsieb G. Non-synonymous to synonymous substitutions suggest that orthologs tend to keep their functions, while paralogs are a source of functional novelty. bioRxiv. 2018):354704.

36. MacFarland TW and Yates JM. Mann–whitney u test. Introduction to nonparametric statistics for the biological sciences using R. Springer; 2016. p. 103-32.

37. Van den Eynden J and Larsson E. Mutational signatures are critical for proper estimation of purifying selection pressures in cancer somatic mutation data when using the dN/dS metric. Front Genet. 2017;8):74.

38. Song H, Bremer BJ, Hinds EC, Raskutti G and Romero PA. Inferring protein sequence-function relationships with large-scale positive-unlabeled learning. Cell Syst. 2020;

39. Pessia E, Popa A, Mousset S, Rezvoy C, Duret L and Marais GA. Evidence for widespread GC-biased gene conversion in eukaryotes. Genome Biol Evol. 2012;4(7):675-82.

40. Zhou Z, Dang Y, Zhou M, Li L, Yu C-h, Fu J, et al. Codon usage is an important determinant of gene expression levels largely through its effects on transcription. Proceedings of the National Academy of Sciences. 2016;113(41):E6117-E25.

41. Sievers A, Bosiek K, Bisch M, Dreessen C, Riedel J, Froß P, et al. K-mer content, correlation, and position analysis of genome DNA sequences for the identification of function and evolutionary features. Genes. 2017;8(4):122.

42. Lee NK, Li X and Wang D. A comprehensive survey on genetic algorithms for DNA motif prediction. Inf Sci. 2018;466):25-43.

43. Noble WS. How does multiple testing correction work? Nat Biotechnol. 2009;27(12):1135-7.

44. Di Rienzo L, Miotto M, Bò L, Ruocco G, Raimondo D and Milanetti E. Characterizing hydropathy of amino acid side chain in a protein environment by investigating the structural changes of water molecules network. Front Mol Biosci. 2021;8

45. Bhadra P, Yan J, Li J, Fong S and Siu SW. AmPEP: Sequence-based prediction of antimicrobial peptides using distribution patterns of amino acid properties and random forest. Sci Rep. 2018;8(1):1-10.

46. Pommié C, Levadoux S, Sabatier R, Lefranc G and Lefranc MP. IMGT standardized criteria for statistical analysis of immunoglobulin V- REGION amino acid properties. J Mol Recognit. 2004;17(1):17-32.

47. Kumar M, Gouw M, Michael S, Sámano-Sánchez H, Pancsa R, Glavina J, et al. ELM—the eukaryotic linear motif resource in 2020. Nucleic Acids Res. 2020;48(D1):D296-D306.

48. Pfleger CM and Kirschner MW. The KEN box: an APC recognition signal distinct from the D box targeted by Cdh1. Genes Dev. 2000;14(6):655-65.

49. Fehr AR and Yu D. Control the host cell cycle: viral regulation of the anaphase-promoting complex. J Virol. 2013;87(16):8818-25.

50. Bösl K, Ianevski A, Than TT, Andersen PI, Kuivanen S, Teppor M, et al. Common nodes of virus–host interaction revealed through an integrated network analysis. Front Immunol. 2019;10):2186.

51. Wright PE and Dyson HJ. Intrinsically disordered proteins in cellular signalling and regulation. Nat Rev Mol Cell Biol. 2015;16(1):18-29.

52. Mészáros B, Erdős G and Dosztányi Z. IUPred2A: context-dependent prediction of protein disorder as a function of redox state and protein binding. Nucleic Acids Res. 2018;46(W1):W329-W37.

53. Hagai T, Azia A, Babu MM and Andino R. Use of host-like peptide motifs in viral proteins is a prevalent strategy in host-virus interactions. Cell Rep. 2014;7(5):1729-39.

54. Michael S, Travé G, Ramu C, Chica C and Gibson TJ. Discovery of candidate KEN-box motifs using cell cycle keyword enrichment combined with native disorder prediction and motif conservation. Bioinformatics. 2008;24(4):453-7.

55. Alanis-Lobato G, Andrade-Navarro MA and Schaefer MH. HIPPIE v2.0: enhancing meaningfulness and reliability of protein–protein interaction networks. Nucleic Acids Res. 2016):gkw985.

56. Abedi M and Gheisari Y. Nodes with high centrality in protein interaction networks are responsible for driving signaling pathways in diabetic nephropathy. PeerJ. 2015;3):e1284.

57. Ozato K, Shin D-M, Chang T-H and Morse HC. TRIM family proteins and their emerging roles in innate immunity. Nat Rev Immunol. 2008;8(11):849-60.

58. Yates AD, Achuthan P, Akanni W, Allen J, Allen J, Alvarez-Jarreta J, et al. Ensembl 2020. Nucleic Acids Res. 2020;48(D1):D682-D8.

59. Shaw AE, Rihn SJ, Mollentze N, Wickenhagen A, Stewart DG, Orton RJ, et al. The antiviral state has shaped the CpG composition of the vertebrate interferome to avoid self-targeting. PLoS Biol. 2021;19(9):e3001352.

60. Chang C-C and Lin C-J. LIBSVM: A library for support vector machines. ACM Trans Intell Syst Technol. 2011;2(3):1-27.

61. Chicco D and Jurman G. The advantages of the Matthews correlation coefficient (MCC) over F1 score and accuracy in binary classification evaluation. BMC Genomics. 2020;21(1):1-13.

62. Yeom S, Giacomelli I, Fredrikson M and Jha S. Privacy risk in machine learning: Analyzing the connection to overfitting. In: *2018 IEEE 31st Computer Security Foundations Symposium (CSF)* 2018, pp.268-82. IEEE.

63. Ali J, Khan R, Ahmad N and Maqsood I. Random forests and decision trees. International Journal of Computer Science Issues (IJCSI). 2012;9(5):272.

64. Zhang M-L and Zhou Z-H. ML-KNN: A lazy learning approach to multi-label learning. Pattern recognition. 2007;40(7):2038-48.

65. Cheng D, Zhang S, Deng Z, Zhu Y and Zong M. kNN algorithm with data-driven k value. In: *International Conference on Advanced Data Mining and Applications* 2014, pp.499-512. Springer.

66. Zhang J, Chai H, Gao B, Yang G and Ma Z. HEMEsPred: Structure-based ligand-specific heme binding residues prediction by using fast-adaptive ensemble learning scheme. IEEE/ACM Trans Comput Biol Bioinform. 2016;15(1):147-56.

67. Liu X-Y, Wu J and Zhou Z-H. Exploratory undersampling for class-imbalance learning. IEEE Trans Syst Man Cybern. 2008;39(2):539-50.

68. Lonsdale J, Thomas J, Salvatore M, Phillips R, Lo E, Shad S, et al. The genotype-tissue expression (GTEx) project. Nat Genet. 2013;45(6):580-5.

69. Papatheodorou I, Moreno P, Manning J, Fuentes AM-P, George N, Fexova S, et al. Expression Atlas update: from tissues to single cells. Nucleic Acids Res. 2020;48(D1):D77-D83.

70. Jeong H, Mason SP, Barabási A-L and Oltvai ZN. Lethality and centrality in protein networks. Nature. 2001;411(6833):41-2.

71. Hahn MW and Kern AD. Comparative genomics of centrality and essentiality in three eukaryotic protein-interaction networks. Mol Biol Evol. 2005;22(4):803-6.

72. Batada NN, Hurst LD and Tyers M. Evolutionary and physiological importance of hub proteins. PLoS Comput Biol. 2006;2(7):e88.

73. Pérez-Martínez D. Innate immunity in vertebrates: an overview. Immunology. 2016;148(2):125-39.

74. Jopling CL. Mutations: Stop that nonsense! Elife. 2014;3):e04300.

75. Zhu X, Pribis JP, Rodriguez PC, Morris Jr SM, Vodovotz Y, Billiar TR, et al. The central role of arginine catabolism in T-cell dysfunction and increased susceptibility to infection after physical injury. Ann Surg. 2014;259(1):171-8.

76. Morris CR, Hamilton- Reeves J, Martindale RG, Sarav M and Ochoa Gautier JB. Acquired amino acid deficiencies: a focus on arginine and glutamine. Nutr Clin Pract. 2017;32):30S-47S.

77. Levring TB, Hansen AK, Nielsen BL, Kongsbak M, Von Essen MR, Woetmann A, et al. Activated human CD4+ T cells express transporters for both cysteine and cystine. Sci Rep. 2012;2(1):1-6.

78. Sikalidis AK. Amino acids and immune response: a role for cysteine, glutamine, phenylalanine, tryptophan and arginine in T-cell function and cancer? Pathol Oncol Res. 2015;21(1):9-17.

79. Yin C, Zheng T and Chang X. Biosynthesis of S-Adenosylmethionine by magnetically immobilized Escherichia coli cells highly expressing a methionine adenosyltransferase variant. Molecules. 2017;22(8):1365.

80. Feld JJ, Modi AA, El–Diwany R, Rotman Y, Thomas E, Ahlenstiel G, et al. S-adenosyl methionine improves early viral responses and interferon-stimulated gene induction in hepatitis C nonresponders. Gastroenterology. 2011;140(3):830-9.

81. Li S-W, Lai C-C, Ping J-F, Tsai F-J, Wan L, Lin Y-J, et al. Severe acute respiratory syndrome coronavirus papain-like protease suppressed alpha interferon-induced responses through downregulation of extracellular signal-regulated kinase 1-mediated signalling pathways. J Gen Virol. 2011;92(5):1127-40.

82. Flo TH, Smith KD, Sato S, Rodriguez DJ, Holmes MA, Strong RK, et al. Lipocalin 2 mediates an innate immune response to bacterial infection by sequestrating iron. Nature. 2004;432(7019):917-21.

83. Tissot C, Rebouissou C, Klein B and Mechti N. Both human α/β and γ interferons upregulate the expression of CD48 cell surface molecules. J Interferon Cytokine Res. 1997;17(1):17-26.

84. Noçon AL, Ip JP, Terry R, Lim SL, Getts DR, Müller M, et al. The bacteriostatic protein lipocalin 2 is induced in the central nervous system of mice with West Nile virus encephalitis. J Virol. 2014;88(1):679-89.

85. Zarama A, Perez-Carmona N, Farre D, Tomic A, Borst EM, Messerle M, et al. Cytomegalovirus m154 hinders CD48 cell-surface expression and promotes viral escape from host natural killer cell control. PLoS Pathog. 2014;10(3):e1004000.

86. Martínez-Vicente P, Farré D, Engel P and Angulo A. Divergent Traits and Ligand-Binding Properties of the Cytomegalovirus CD48 Gene Family. Viruses. 2020;12(8):813.

87. Ricquier D. UCP1, the mitochondrial uncoupling protein of brown adipocyte: a personal contribution and a historical perspective. Biochimie. 2017;134):3-8.

88. Yu X, Liu H, Hamel KA, Morvan MG, Yu S, Leff J, et al. Dorsal root ganglion macrophages contribute to both the initiation and persistence of neuropathic pain. Nat Commun. 2020;11(1):1-12.

89. Chen Y, Lun AT and Smyth GK. From reads to genes to pathways: differential expression analysis of RNA-Seq experiments using Rsubread and the edgeR quasi-likelihood pipeline. F1000Research. 2016;5

90. Herrero J, Muffato M, Beal K, Fitzgerald S, Gordon L, Pignatelli M, et al. Ensembl comparative genomics resources. Database. 2016;2016):bav096.

91. Li HD, Menon R, Omenn GS and Guan Y. Revisiting the identification of canonical splice isoforms through integration of functional genomics and proteomics evidence. Proteomics. 2014;14(23-24):2709-18.

92. Wang ET, Sandberg R, Luo S, Khrebtukova I, Zhang L, Mayr C, et al. Alternative isoform regulation in human tissue transcriptomes. Nature. 2008;456(7221):470-6.

93. Sieber P, Platzer M and Schuster S. The definition of open reading frame revisited. Trends Genet. 2018;34(3):167-70.

94. Pan Q, Shai O, Lee LJ, Frey BJ and Blencowe BJ. Deep surveying of alternative splicing complexity in the human transcriptome by high-throughput sequencing. Nat Genet. 2008;40(12):1413-5.

95. Guéguen L and Duret L. Unbiased estimate of synonymous and nonsynonymous substitution rates with nonstationary base composition. Mol Biol Evol. 2018;35(3):734-42.

96. Takata MA, Gonçalves-Carneiro D, Zang TM, Soll SJ, York A, Blanco-Melo D, et al. CG dinucleotide suppression enables antiviral defence targeting non-self RNA. Nature. 2017;550(7674):124-7.

97. Yu C-H, Dang Y, Zhou Z, Wu C, Zhao F, Sachs MS, et al. Codon usage influences the local rate of translation elongation to regulate co-translational protein folding. Mol Cell. 2015;59(5):744-54.

98. Tufarelli C, Ahmad A, Strohbuecker S, Scotti C and Sottile V. In Silico Identification of SOX1 Post-Translational Modifications Highlights a Shared Protein Motif. 2020;

99. Yoon J, Blumer A and Lee K. An algorithm for modularity analysis of directed and weighted biological networks based on edge-betweenness centrality. Bioinformatics. 2006;22(24):3106-8.

100. Friedel CC and Zimmer R. Influence of degree correlations on network structure and stability in protein-protein interaction networks. BMC Bioinformatics. 2007;8(1):1-10.

101. Ravasz E, Somera AL, Mongru DA, Oltvai ZN and Barabási A-L. Hierarchical organization of modularity in metabolic networks. Science. 2002;297(5586):1551-5.

Figure 1

Click here to access/download;Figure;Figure_1.eps

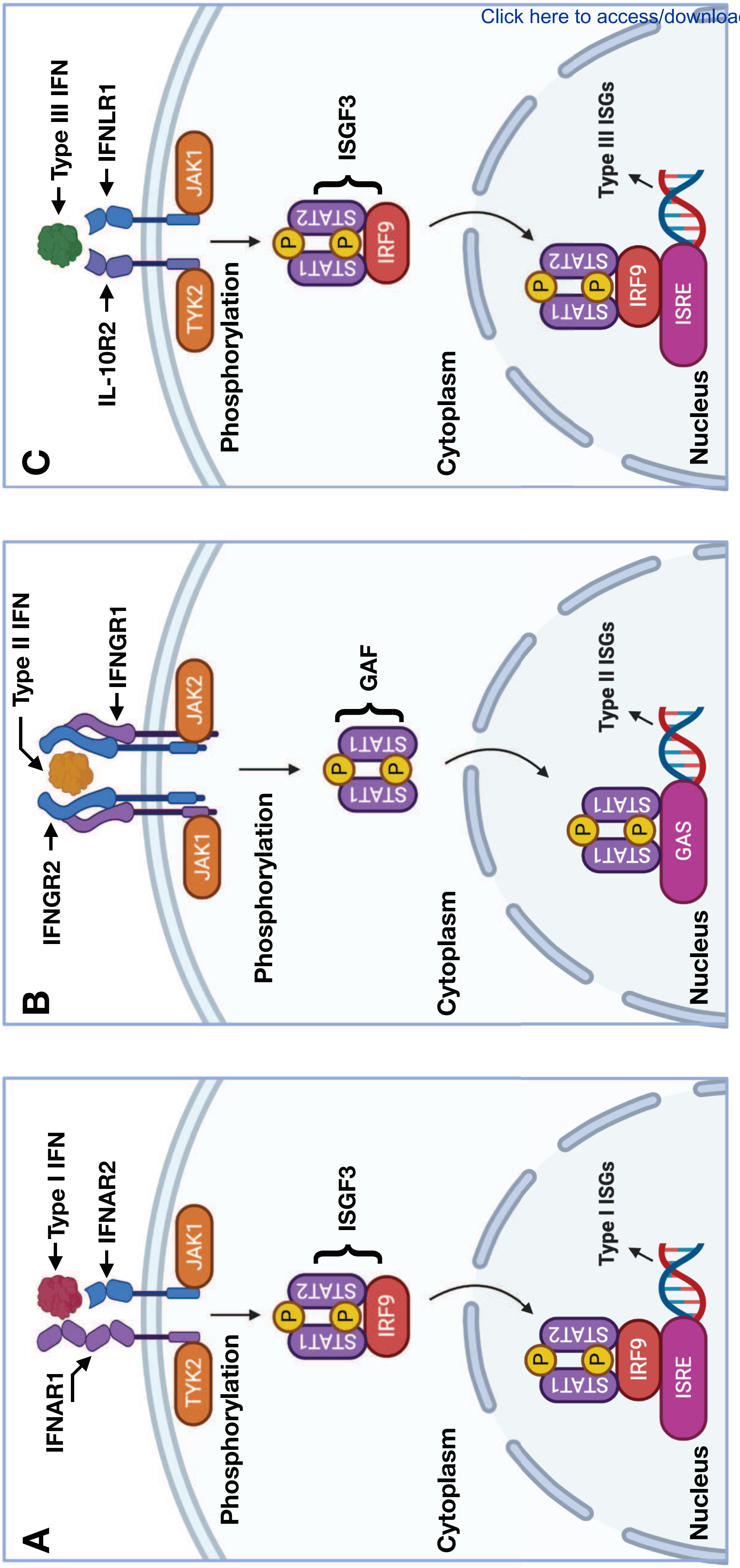

Click here to access/download;Figure;Figure_2.eps

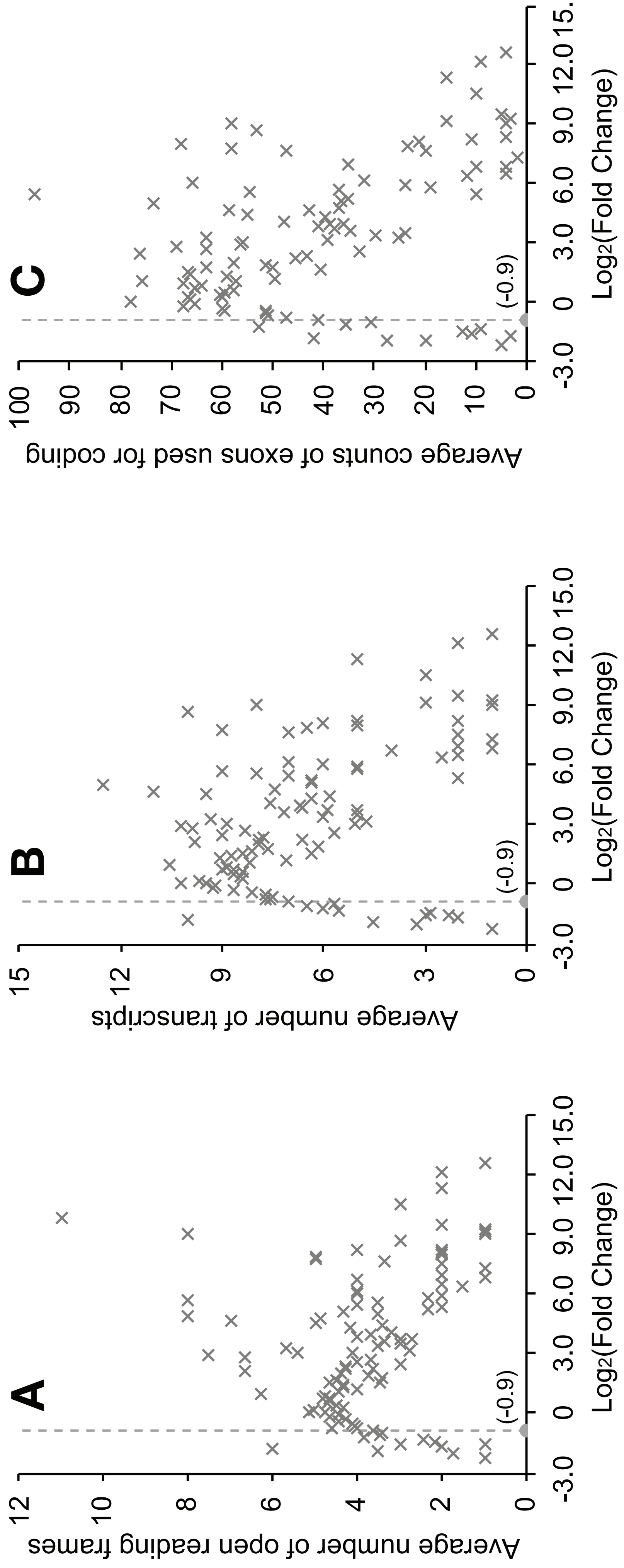

Figure 3

Click here to access/download;Figure;Figure_3.eps

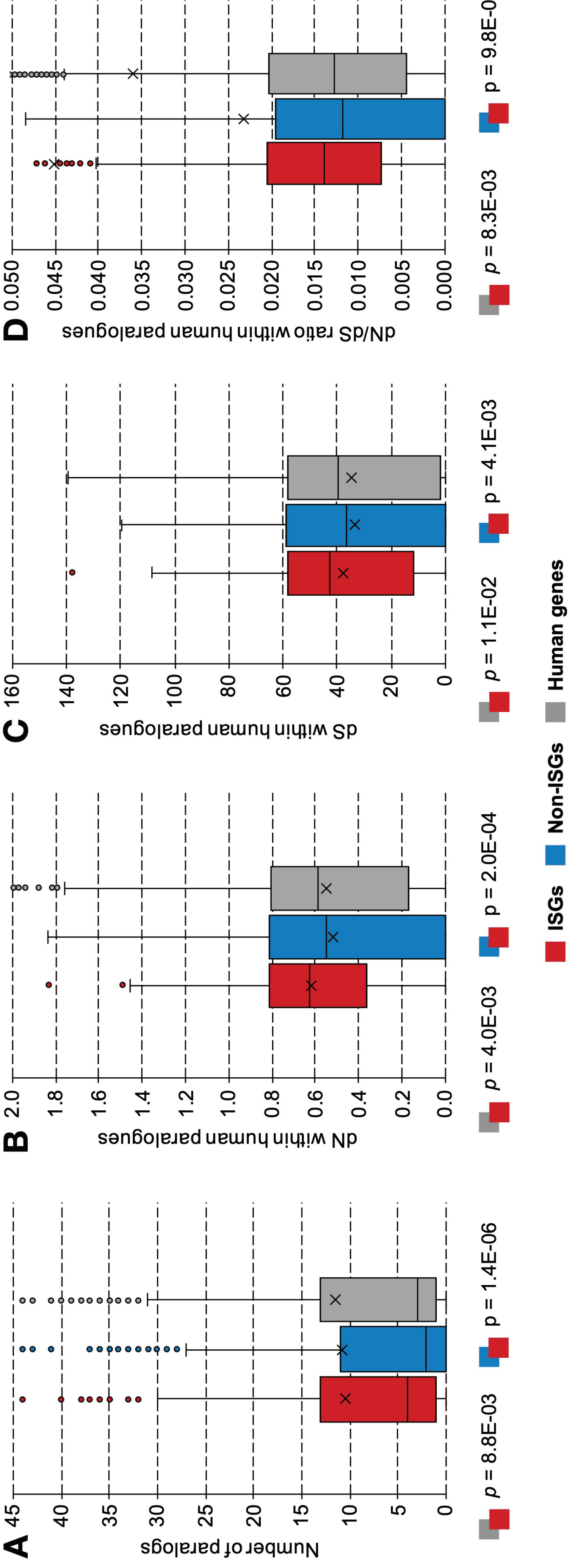

Figure 4

Click here to access/download;Figure;Figure_4.eps

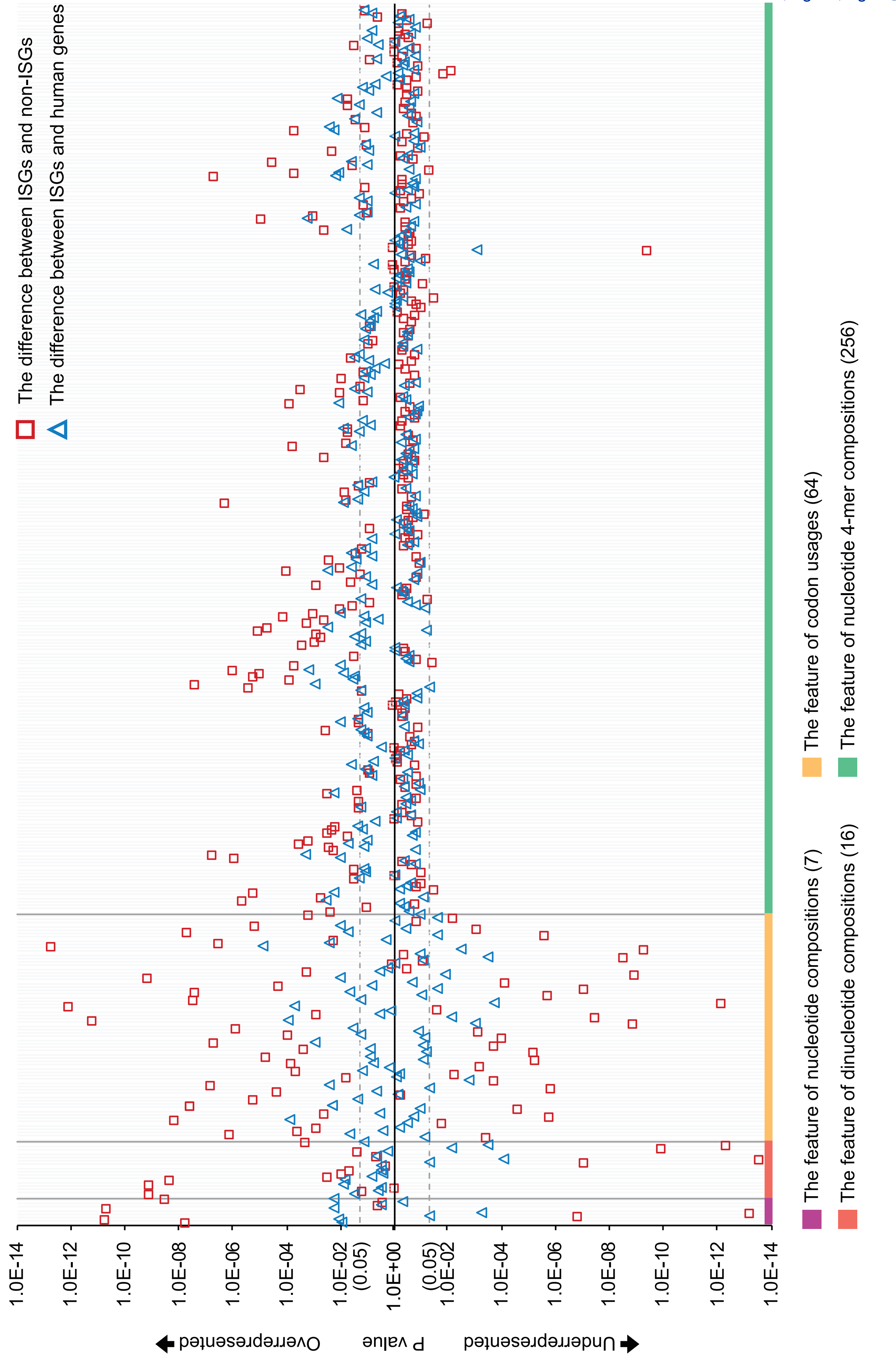

Figure 5

Click here to access/download;Figure;Figure_5.eps

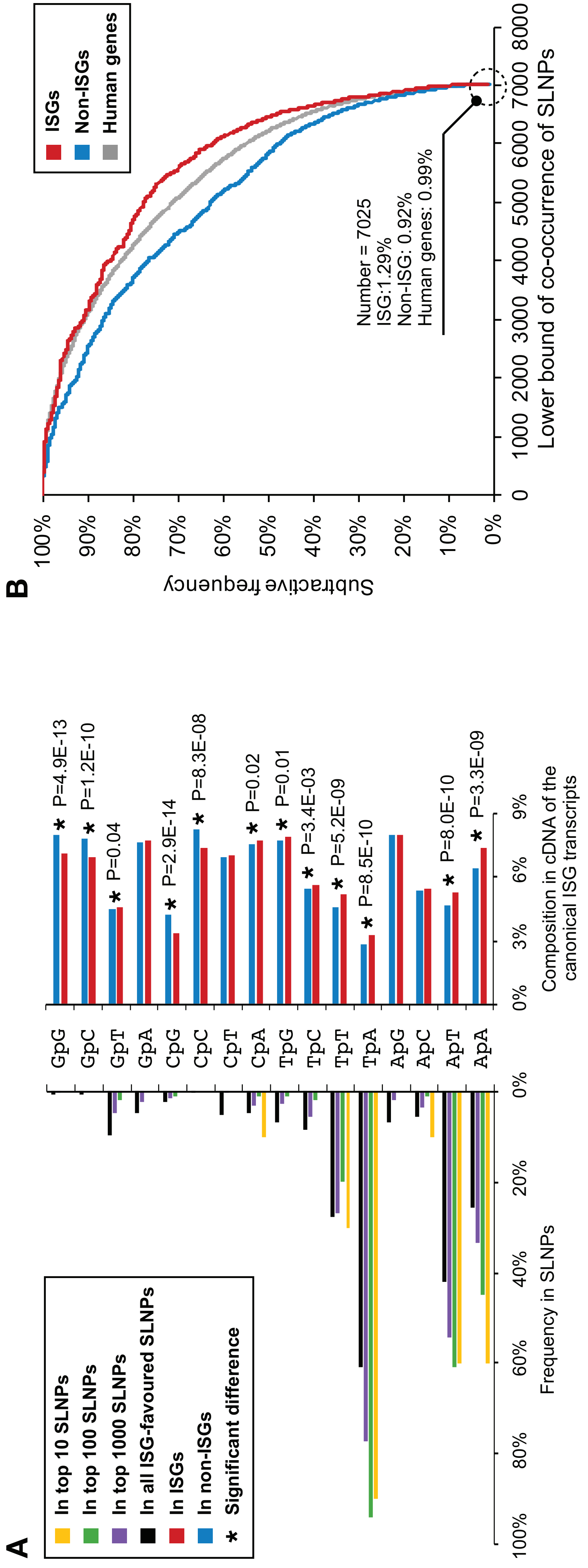

Figure 6

Click here to access/download;Figure;Figure_6.eps

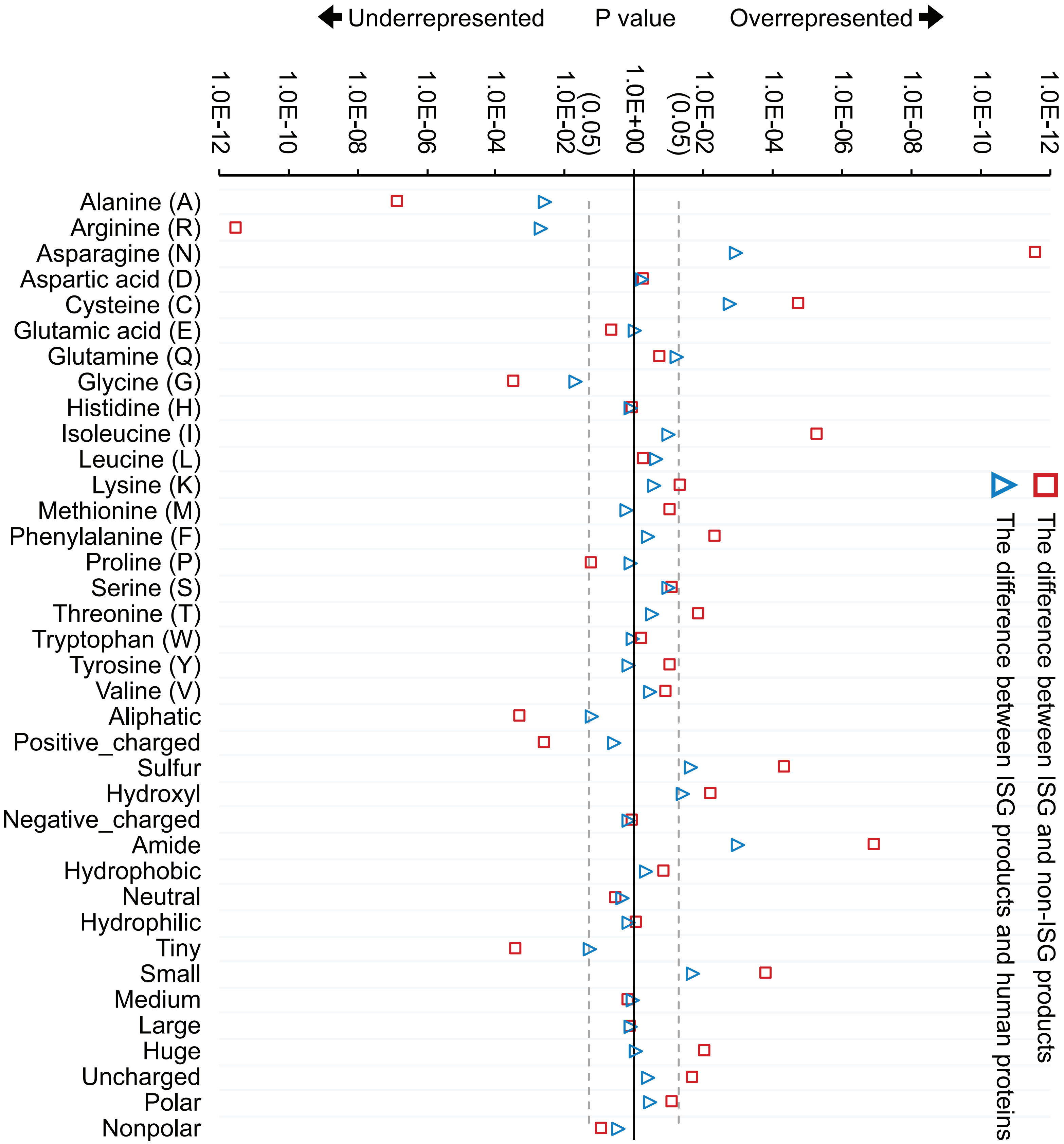

Figure 7

Click here to access/download;Figure;Figure_7.eps

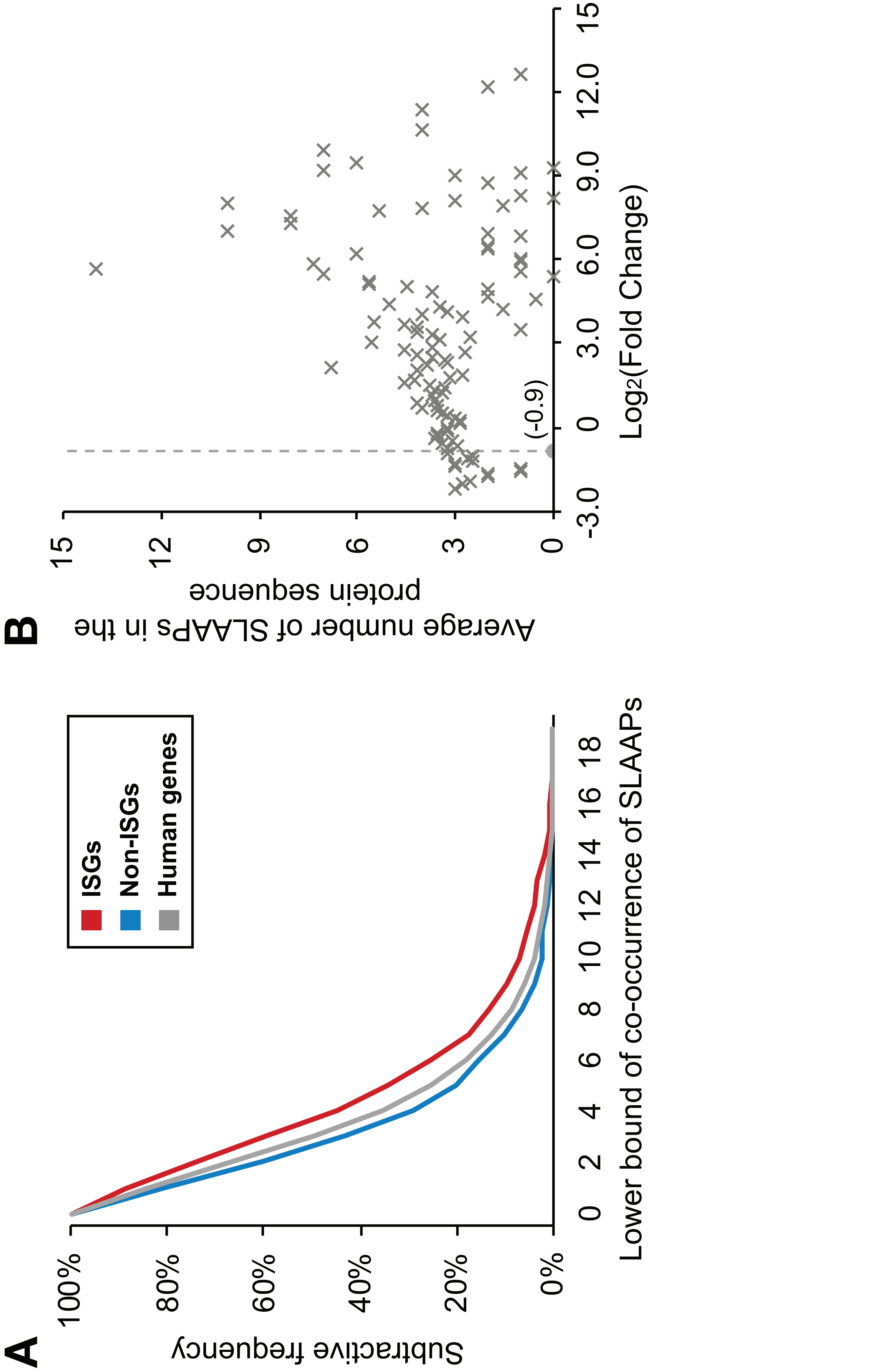

Figure 8

Click here to access/download;Figure;Figure_8.eps

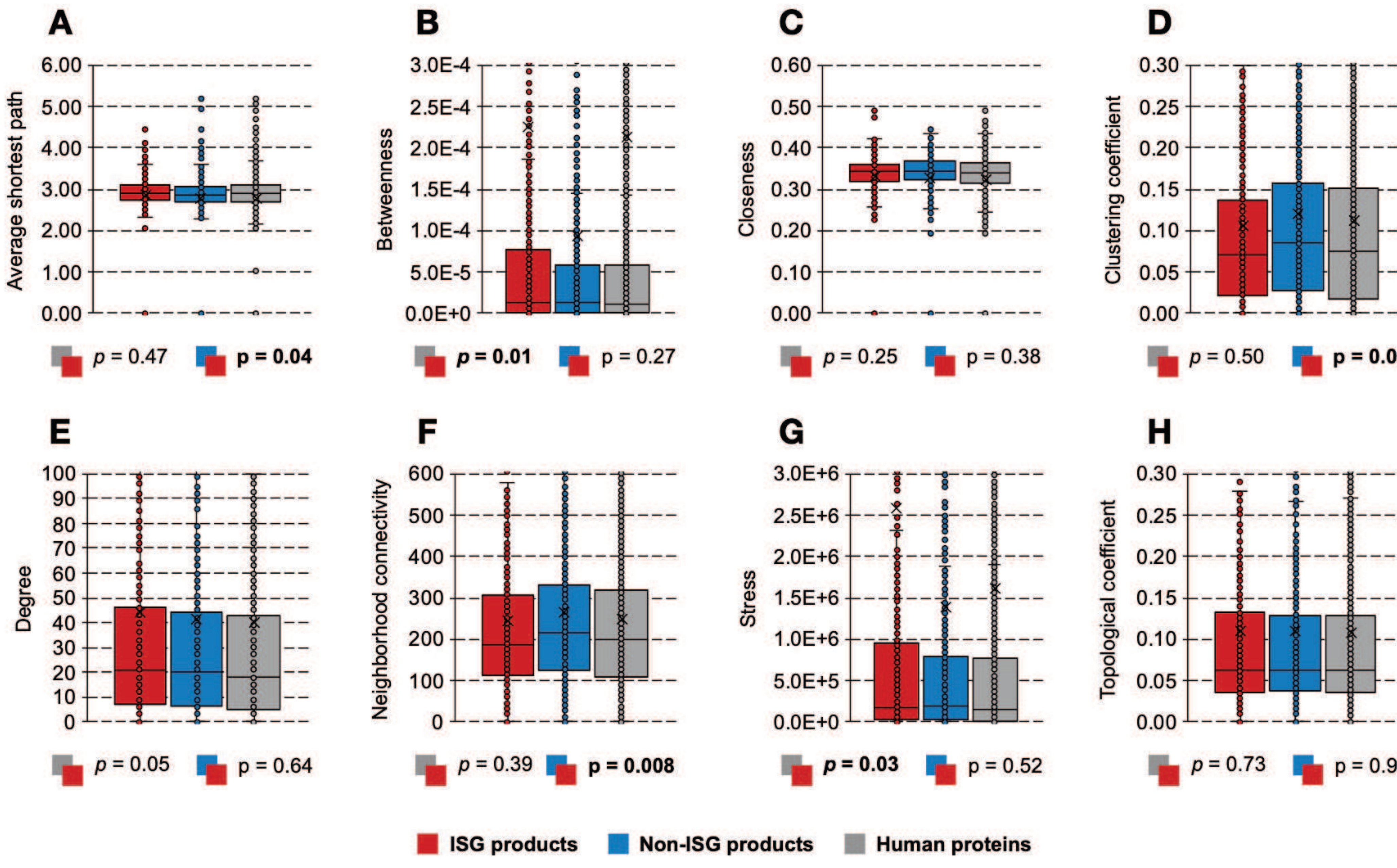

Figure 9

Click here to access/download;Figure;Figure_9.eps

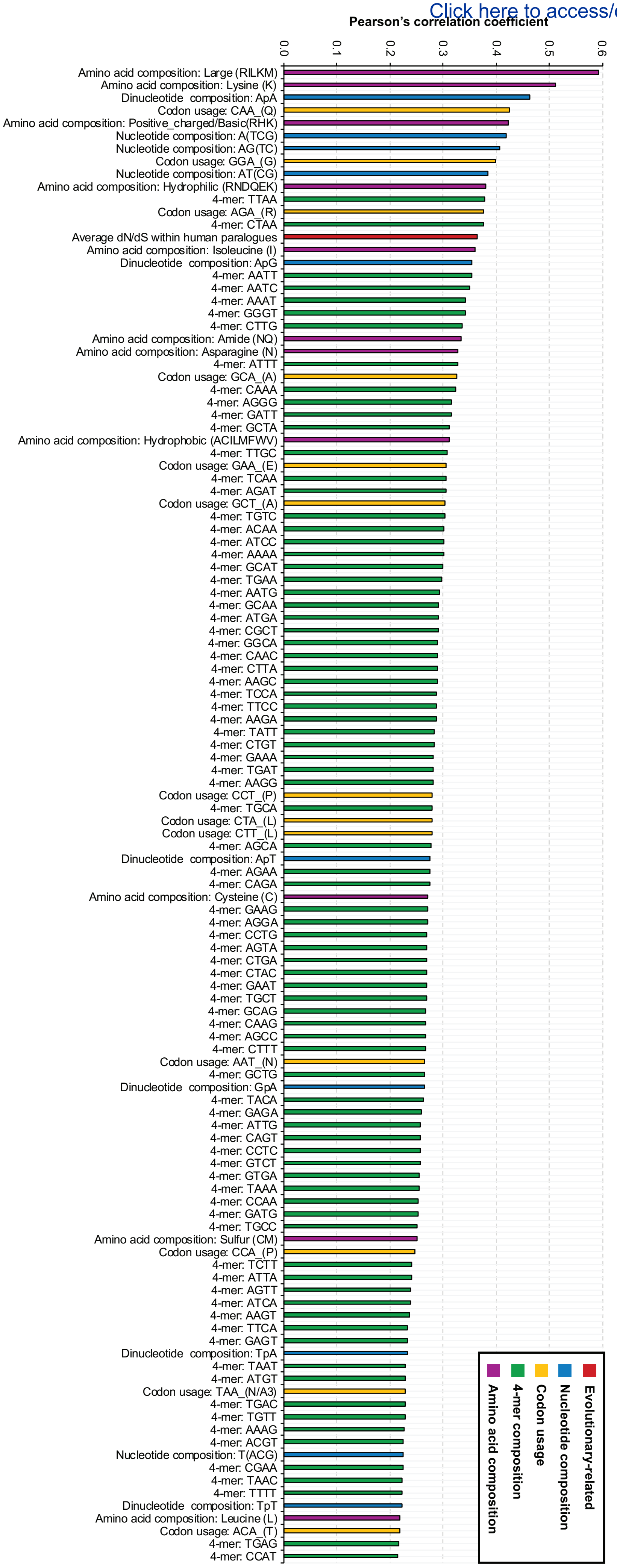

Figure 10

Click here to access/download;Figure;Figure_10.eps

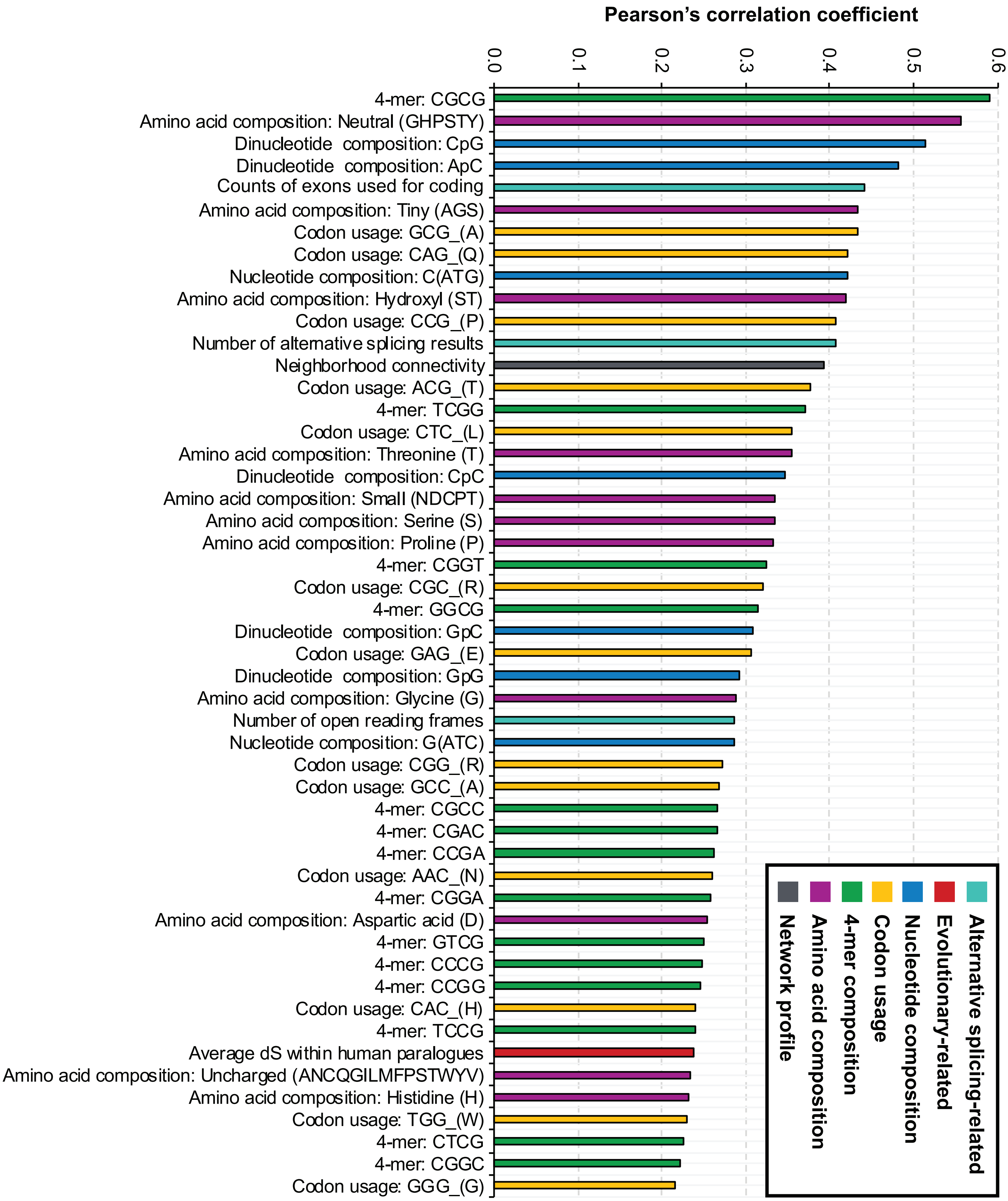

Figure 11

Click here to access/download;Figure;Figure_11.eps

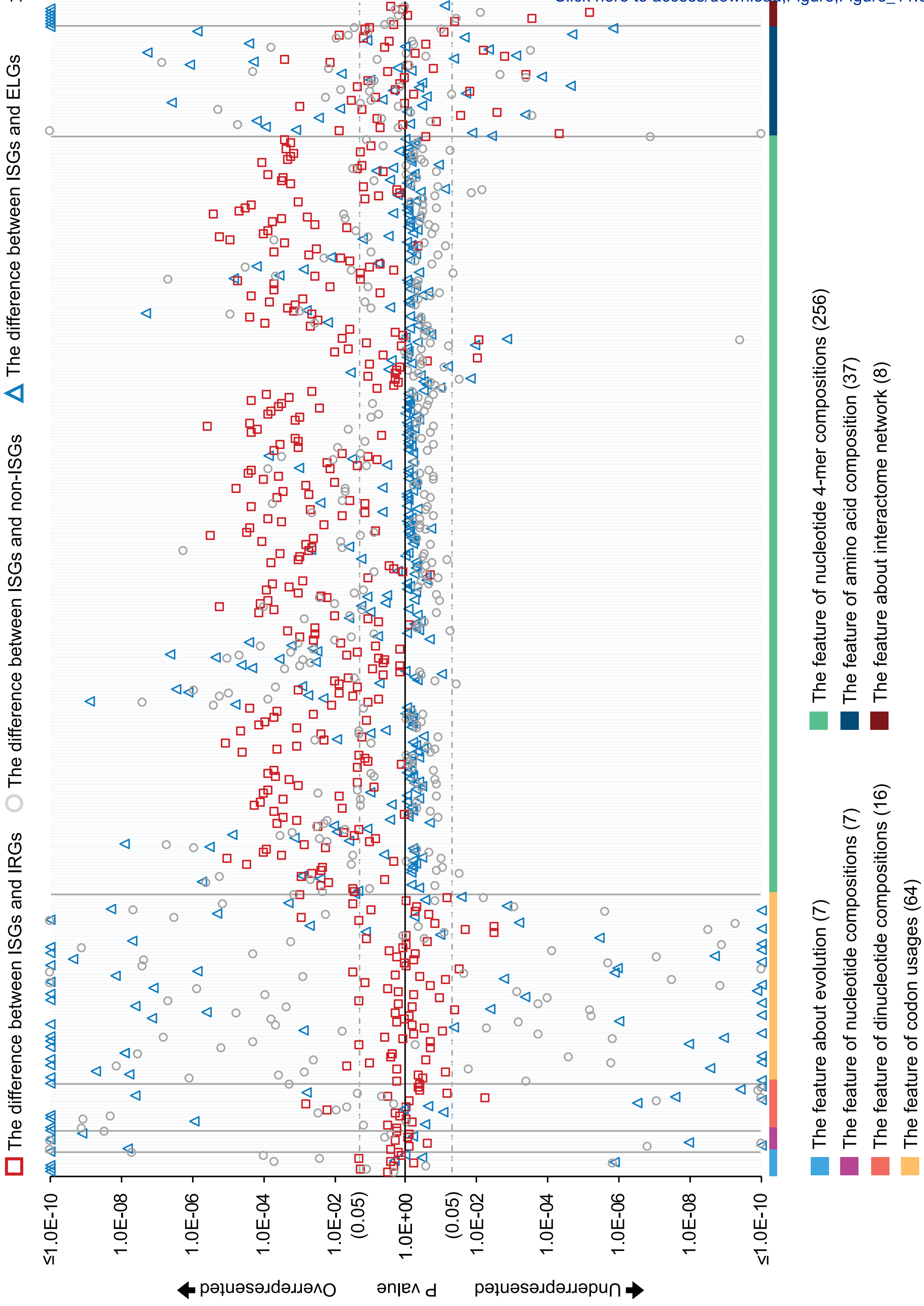

Figure 12

Click here to access/download;Figure;Figure_12.eps

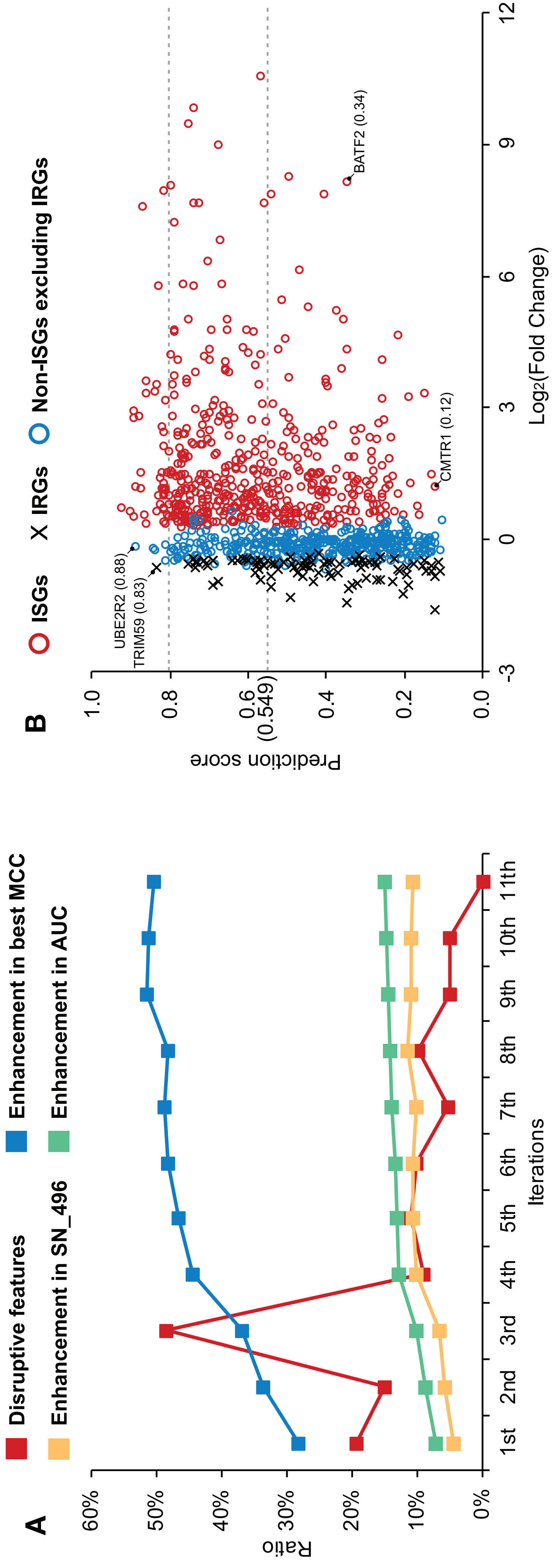

Figure 13

Click here to access/download;Figure;Figure_13.eps

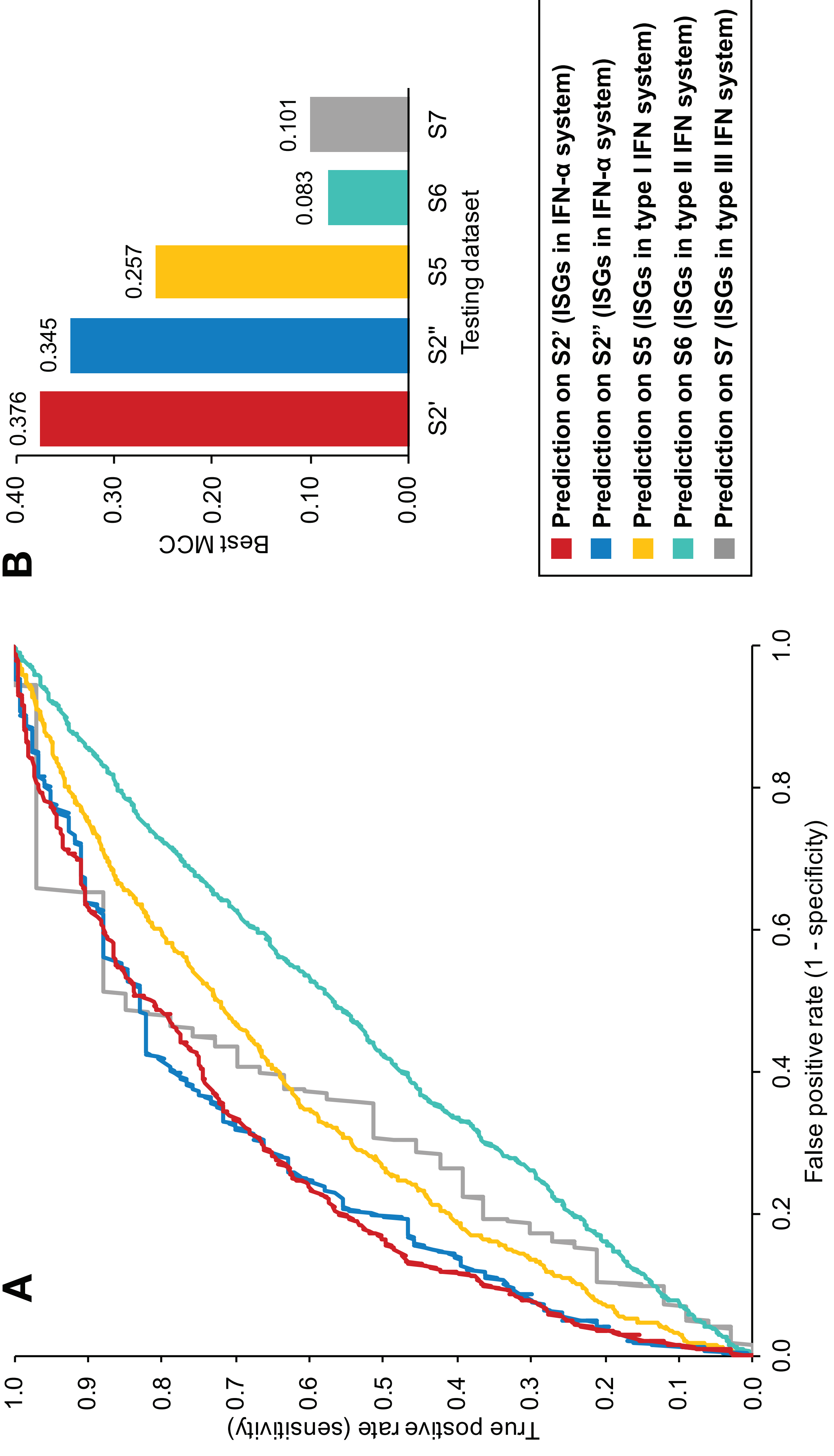

Figure 14

Click here to access/download;Figure;Figure_14.eps

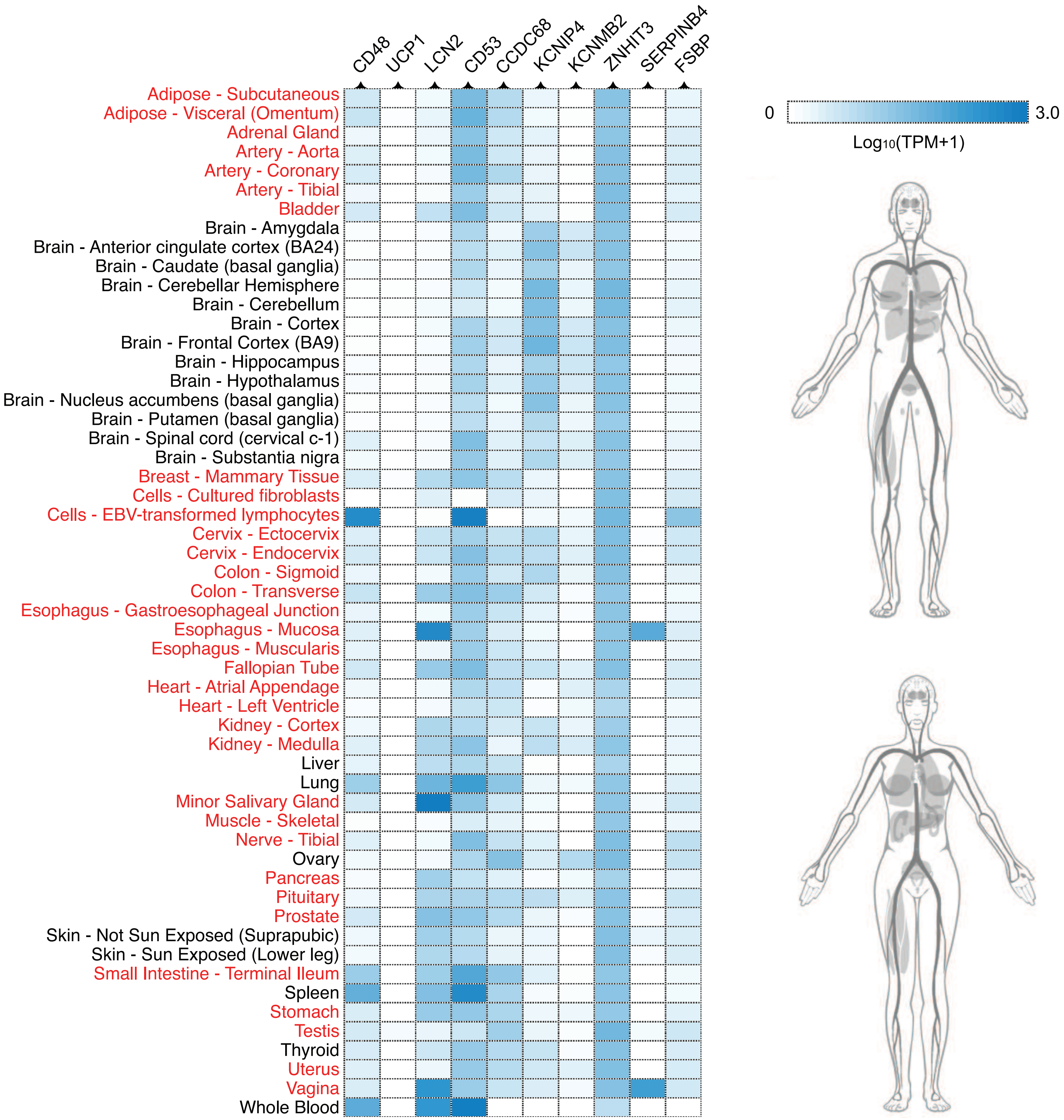

Figure 15

Click here to access/download;Figure;Figure_15.eps

BEGIN

Initialisation: Balanced dataset $S_0 = \{(1, v_1^0), .. (1, v_n^0), (0, v_{n+1}^0) ... (0, v_{2n}^0)\}$, dimension of the feature vector D_0, machine learning algorithm A, number of disruptive feature $d_0 = D_0$, and iteration round $i = 0$.

While $d_0 > 0$ (i^{th} iteration):

1) Use five-fold cross validation on dataset S_i, prediction $P_i = A(S_i)$;
2) Evaluate the P_i with the criterion of AUC;
3) Remove one feature from feature vector v^i and generate a temporary dataset T_i;
4) Use five-fold cross validation on dataset T_i, prediction $P'_i = A(T_i)$;
5) Evaluate the P'_i with the criterion of AUC;
6) Repeat 4) and 5) for the traversal of D_i features;
7) Traverse v^i and remove m features helpful to improve AUC of P'_i, $d_i = m$;
8) Update dataset $S_{i+1} = \{(1, v_1^{i+1}), .. (1, v_n^{i+1}), (0, v_{n+1}^{i+1}) ... (0, v_{2n}^{i+1})\}$, $D_{i+1} = D_i - m$.

End

Output: dataset S_{i-1} encoded by D_{i-1} features.

END

Click here to access/download
Supplementary Material
Supplementary_Data_S1.csv

Supplementary Material 2

Click here to access/download
Supplementary Material
Supplementary_Data_S2.csv

Supplementary Material 3

Click here to access/download
Supplementary Material
Supplementary_Data_S3.csv

Supplementary Material 4

Click here to access/download
Supplementary Material
Supplementary_Data_S4.csv

Click here to access/download

Supplementary Material

Supplementary_Data_S5.txt

Personal Cover Click here to access/download;Personal Cover;Cover_letter.docx

Editors

GigaScience

24th Feb 2022

Dear Editors

On behalf of my co-authors please consider our research article entitled 'Defining the characteristics of interferon-alpha-stimulated human genes: insight from expression data and machine learning' for consideration in your journal. We present systematic data analyses on large-scale features to characterise the association between the response of human genes to interferons-α (IFN-α) and their inherent properties. Our results show that the up-regulated interferon-α stimulated genes (ISGs) differentially represent many features that make them distinguishable from those not significantly up-regulated (non-ISGs) in the presence of IFN-α. We find that the IFN-α repressed human genes (IRGs) have some shared properties with the ISGs. We apply machine learning ideas with an original feature selection strategy to prove the predictability of the ISGs. Our prediction method is implemented as a web server at http://isgpre.cvr.gla.ac.uk/. The source code, prediction model, and all feature profiles are released at https://github.com/HChai01/ISGPRE for reproducible use. We believe our article will be of interest to the international research community, and thus will be of interest to your readership. We confirm that this manuscript has not been published elsewhere, is not under consideration by any other journal, and that all authors have read and approved the submission of the manuscript.

Yours Sincerely,

Joseph Hughes

Medical Research Council-University of Glasgow Centre for Virus Research
Sir Michael Stoker Building, Garscube Campus, 464 Bearsden Road, Glasgow G61 1QH
cvr.ac.uk @CVRInfo
The University of Glasgow, charity number SC004401.
