# Supplementary material for: Defining the characteristics of interferon-alpha–stimulated human genes: insight from expression data and machine learning
Source: Gigascience. 2022 Nov 18;11:giac103. doi: 10.1093/gigascience/giac103 (PMC9673497; doi:10.1093/gigascience/giac103)
Supplement: giac103_Response_to_Reviewer_Comments_Original_Submission [file giac103_response_to_reviewer_comments_original_submission.pdf]

Author's Response To Reviewer Comments

Close

Comments from Reviewer #1:
In this manuscript, the authors analyzed different characteristics that are potentially related to the expression of human genes under IFN-a stimulation. A classification model is built to predict ISG (genes that are upregulated following IFN-a stimulation) from the human fibroblast cell. The model also performs feature selection, and the authors used different test sets (on different types of IFN) to validate their model. The authors provide a web server that implemented this machine learning model.

Key comment 1: I liked the introduction, the background and motivation were clear. However, the Results section was a bit hard to follow, in particular the implementation of the machine learning models, with different classifiers applied inconsistently across distinct feature sets.

Our reply: Thanks for the suggestions. We have changed the structure of the results section and added some appendices to enhance the readability of our manuscript. Table 3 now includes two main parts:
1) comparison of different machine learning methods (KNN, RF and SVM);
2) comparison of SVM classifiers optimised by different feature selection strategies (FFS and ASI);
Due to the rationale behind random forest, the final number of features shown in Table 3 is not 518. The result of the first comparison proves the effectiveness of SVM. The result of the second comparison proves the effectiveness of our feature selection strategy (see Figure 15). We have amended the structure of Table 3 to make it more understandable.

Key comment 2: Regarding reproducibility, the authors provide a Github repository with source code, the model trained and data. From the documentation and notes in the manuscript (lines 1015-1023), looks like this can only be run on mac OS, which makes it very hard for me to test (I'm a Linux user). I recommend the authors to read and follow the article "Reproducibility standards for machine learning in the life sciences" (https://doi.org/10.1038/s41592-021-01256-7). Having, for instance, a Docker image to download and run your analyses would be fantastic.

Our reply: Thanks for your comments. We have added a Docker implementation for our machine learning method. The image is available at https://hub.docker.com/r/hchai01/isgpre. Instructions can be found at our GitHub repository (https://github.com/HChai01/ISGPRE).

Key comment 3: The authors perform a comprehensive analysis of features that differentiate different gene classes. I wonder why didn't they use first a machine learning model to automatically find these important features, and then try to analyse which features were selected (instead of the other way around as done in the study). I think there is perhaps too much manual feature engineering in the previous steps of training an ML model.

Our reply: Thanks for the comment. The analyses and machine learning are separated in our project. In the analyses, we aim to find as many 'important' features as possible but in the machine learning, we aim to find an optimal way to classify the considered classes with limited information. It should be noted that some samples used for feature analyses were not included in the training or modelling stage as they were randomly selected for independent testing (see the newly added Figure 2). Such random sampling procedures will change the distribution of the features, especially those for the major class (non-ISGs), which means the feature processed in the machine learning stage is not the same as the one used in the analysis stage. In other word, the feature distribution of samples used for training can't truly reflect the natural distribution of the considered classes. Some key insights may be missed if we first use machine learning models to find 'important' features for later analyses. Lastly, we have optimised our machine learning pipeline to make it easy to follow.

Key comment 4: Related to the previous point, in my comments below one of my concerns is about feature correlation. The authors compare individual features regarding their ability to separate different gene classes (ISG vs background vs non-ISG). But one can imagine that some features are highly correlated. Some features might not be useful to separate gene classes from a single-feature analysis (as the authors do at the beginning), but they could be useful in combination with other features. Unless I'm missing an important point, I would leave the machine learning model to learn this and then analyze each feature individually after the model identifies them.

Our reply: Yes, you are right. The combination of some features can contribute to separate gene classes. Machine learning models do help to identify this. Features with high importance in machine learning have a higher chance to have differential distribution in nature but it is not guaranteed due to random sampling. On the other hand, features with better discrimination in analyses may have a higher chance to enhance the quality of the machine learning model but it is also not guaranteed (see Figure 9-11 & Table 4). The clues shown in both analyses and machine learning can further highlight some features that make a gene stimulated under IFN-alpha. However, it is not a good reason to ignore the contribution of some features in identifying ISGs just because they are not performing well in the machine learning stage. It is acceptable to put the machine learning before or after the feature analyses. We put feature analyses first because this paper is mainly focused on finding out what changes the expression of a human gene following IFN-alpha stimulation. Machine learning is our strategy to see if some features can be used to identify ISGs in a high-throughput way.

Key comment 5: Authors are concerned that including too many features in the support vector machine (SVM) model would complicate the prediction task. To remedy this, they manually select the features according to, in my opinion, a more subjective criterion. Why didn't the authors use a feature selection algorithm here? I know that they propose a model including feature selection, but I guess I don't understand well all the previous manual feature analyses. Using a known feature selection method here would provide a more data-driven approach to improve classification, in addition to their manual expert curation (which is also valid).

Our reply: Thanks for the suggestion. We have added the comparison among different feature selection strategies to prove the effectiveness of ours.

Key comment 6: They run several classification models, but not consistently across the same set of features. For example, only SVM is run across genetic, parametric, all features, etc, but not the other models. Why is that?

Our reply: Thanks for the comments. As previously mentioned, the comparisons shown in Table 3 first identify which base machine learning method performs best. We then use the best-performing method (SVM) to test the performance of different feature sets. We have amended the structure of Table 3 to make it easier to understand.

Key comment 7: The manuscript would really benefit from a figure with the main steps of the analyses performed, models tested, datasets employed, etc. It's hard to get the big picture as it is now.

Our reply: Thanks for your suggestion. We have added a figure to show this (see new Figure 2).

Key comment 8: I think the window size used (mentioned in the text) should be added to the Figure 2 caption.

Our reply: Thanks for your suggestion. We have added it to the caption (see new Figure 3).

Key comment 9: * What's the vertical dashed line? In the text, you say that those at the left of this line are IRGs, but I don't understand the meaning of that vertical line (-0.9 log fold change). This explanation, which I didn't see, should be added to the figure caption also.

Our reply: Thanks for the comment. In our collected data, the log fold change of IRGs are all lower than -0.9. That’s why we mentioned ‘that those at the left of this line are IRGs’. We have updated the figure

and divided each plot into three regions. All data points in the left region come from IRGs (Log2(FoldChange) < -0.871); points in the right region all come from ISGs (Log2(FoldChange) > 0.686); points in the middle region may come from ISGs or non-ISGs (including IRGs).

Key comment 10: From the text, I understand that in the subfigures in Figure 2 you have IRGs, non-ISGs and ISGs. Would it be possible, or meaningful for the reader, to add an extra vertical line to separate them?

Our reply: Thanks for the comment. Current vertical line (x=-0.871) is used to separate some but not all IRGs. We have added a new vertical line to separate some ISGs (x=0.686). However, the source of data points in the region between x=−0.871 and x=0.686 are complex. They may come from ISGs or non-ISGs (including IRGs). We have added some description in the figure caption.

Key comment 11: If GC-content is underrepresented in ISGs more than non-ISGs, the ApT and TpA should be expected to be more enriched in ISGs, right? Sounds like a redundant analysis. I would expect these two sequence-derived features to be correlated. If this is the case, maybe it would be better to highlight other features instead of a correlated/expected one?

Our reply: Thanks for the suggestion. The depletion of GC-content in ISGs has some impacts on the representation of dinucleotide composition, codon usages and amino acid composition. We expect the representations of some GC-related features may be underrepresented but we cannot tell more unless those features were analysed. For instance, it's hard to tell whether the depletion of CpG or GpC is more important to the stimulation of human genes under IFN-alpha. Therefore, these analyses are not redundant as long as they are not completely the same (e.g., GC-content, CG-content or AT-content).

Key comment 12: Figure 4: here the authors divided the parametric set of features into four categories and compared their representations among ISGs, non-ISGs and background genes. The figure shows p-values of the tests on the y-axis, and the four categories of features on the x-axis. I think it's important to run a negative control: could you please run these tests again, say, 100 times, with gene IDs/names shuffled, and check whether some of these results also appear in these null simulations? Maybe you can keep the same figure but remove those also found in the null simulations.

Our reply: Thanks for the comments. First of all, the red squares in this figure (now Figure 5) show the comparisons of some genome-based features between the stimulated class (ISGs) and non-stimulated class (non-ISGs). The blue triangles are also placed in the same figure as the restriction of filtering 'high confident' non-ISGs may also have some impacts to form a 'special' distribution differential to ISGs'. We figure that the negative control may not be helpful here as the features we analysed are all inherent thus will not change due to the impact of IFN-treatment. We do have some samples with almost invisible changes in the experiments. They are called ELGs and the comparison between ELGs and ISGs are shown in Figure 11. We have updated the caption of the figure to make it easier to understand.

Key comment 13: Is it possible that the comparison of codons frequencies (third category of features) is correlated with previous findings (like GC content or ApT/TpA enrichment)? If so, would it be possible that maybe the analysis is also expected or redundant? For example, in ISGs there is an underrepresentation of GC-content, and you also found that ISGs there is an underrepresentation of "CAG" codons. I might be missing something, but aren't these expected to be correlated?

Our reply: Yes, you are right. The codon usages are influenced by the nucleotide composition in the CDs. The analysis can be expected but is not redundant. As we mentioned in the reply to your key comment 11, we aim to have better understanding of each feature rather than expecting that they are over- or under-represented in ISGs.

Key comment 14: Figure 6: I would suggest adding the same negative control suggested before.

Our reply: Thanks for the comments. We believe the negative control may not be helpful here as the representation of features are not influenced by the IFN experiments.

Key comment 15: I think it's important to define what are all those eight features in the network analyses (closeness, betweenness, etc), otherwise it's hard to follow what comes next.

Our reply: Yes, you are right. We have already provided this information in the Method Section: 'Generation of discrete features'. Please check the last paragraph of that section for details.

Key comment 16: Figures 9 and 10: it would be good to add the sign of the correlation in the figure, in addition to mentioning it in the caption (as it is now).

Our reply: Thanks for your suggestion. We have corrected the figure about negative correlation (see new Figure 11). The sign now can be found in the y-axis. We have also added some description in the figure caption. Please check new Figure 10/11 for details.

Key comment 17: Given the unique patterns or differences between non-ISG class and IRG class, wouldn't it be better to perform different analyses excluding IRG genes? The authors also acknowledge these risks in lines 539-541.

Our reply: Thanks for your suggestion. However, the main focus of the current paper is to identify what makes a human gene stimulated in the presence of IFN-alpha. The investigation of IRGs is a side analysis to show that it does not influence the definition of a 'null stimulation'.

Key comment 18: It was hard for me to understand the workflow in this section: you used different machine learning models applied to distinct features sets, for example. Why don't you apply the same set of models to the same set of features? I think this section needs an initial paragraph with a global description of what you are trying to do.

Our reply: Thanks for your suggestion. The workflow in this section is: 1) find the best-performed base method; 2) find the optimal feature set; 3) train the machine learning model with the best-performing base method and optimal feature set. The final model is then used for testing the 7 test datasets mentioned in Table 5. We have added a global description at the beginning of this section to make it easier to follow.

Key comment 19: For example, I don't think I understand very well the concept of "disruptive feature". What does it mean?

Our reply: Thanks for the comments. A feature is identified as 'disruptive' if the overall performance of the classifier becomes worse after being added. We have changed it to 'noisy' in the hope that this is more understandable.

Key comment 20: Table 3: I don't understand the threshold selection here. I guess you refer to classification or decision threshold from a model that outputs a probability of a gene to be ISG or non-ISG. First, I think there should be a line separating each performance measure to clearly show those that are "Threshold-dependent" and "Threshold independent"

Our reply: Yes, you are right. Thanks for the suggestion. We have added a line to separate the threshold-dependent and threshold-independent criteria.

Key comment 21: I also understand that, during cross-validation, you selected for each model/feature set combination, the threshold that maximized the MCC (this is explained in Table 3 as a footnote, but it should be more explicitly mentioned in the text).

Our reply: Thanks for the suggestion. We have added some description for it.

Key comment 22: Table 3: What is the "Optimum" set of features? Why is this "Optimium set" only used

with SVM?

Our reply: Thanks for the comments. The ‘optimum’ set of features are generated via our feature selection scheme (Figure 16). The workflow in this section is first identify the best-performing machine learning method then use it (SVM) with the feature selection strategy to identify the ‘optimal’ feature set (No.=74). We have added some further description in the footnote of Table 3.

Key comment 23: How does the "AUC-driven subtractive iteration algorithm (ASI)" compare with other feature selection algorithms.

Our reply: Thanks for the comments. Our feature selection method is developed based on the ‘Backward Feature Elimination’ scheme. We have compared it with another important Sequential Feature Selection method: ‘Forward Feature Selection’ scheme. Please check Table 3 and Results section: ‘Implementation with machine learning framework’ for details.

Key comment 24: Table 5: you mention this in the text, but it would be good to have an extra column indicating which datasets were used for training and which are for testing.

Our reply: Thanks for the suggestion. We have reshuffled the structure of Table 5 to make this clear.

Key comment 25: Figure 13: it would be good to have the AUROC in the figure, not only the curves.

Our reply: Thanks for the suggestion. We have added 'ROC' note in Figure 13.

Comments from Reviewer #2:
First of all, this manuscript is well-written after a thorough research investigation. I enjoyed reading about interferons, interferon stimulating genes (ISGs), mechanisms and signalling pathways. In the introduction, the authors have highlighted the different methods (including other bioinformatics databases) available to identify ISGs and their potential pitfalls. This unmet need is addressed using in silico approaches which were used to classify interferon stimulating genes from non-stimulating ones in human fibroblast cells. Here, the authors have applied a combination of expression data and sequential/compositional features and designed a machine learning model for the prediction of ISGs from non-ISGs.

Apart from features like duplication, alternative splicing, mutation and presence of multiple ORFs, the authors extracted various sequential features and found them to be correlated well with ISG prediction. For example, ISGs are prone to GC depletion and a significant difference in the codon usage among ISGs was found. In that context, the authors claim that ISGs are evolutionarily less conserved, codon usage features, genetic composition features, proteomic composition features and sequence patterns (especially like SLNPs and SLAAPs) are optimal parameters that can cumulatively help in differentiating ISGs from non-ISGs.

When it comes to building a machine learning model, the authors faced challenges due to similarities between ISGs and IRGs. They have experimented using different algorithms for model building ranging from the decision tree, and random forest and found decent results with support vector machine.

Key comment 1: Model Prediction accuracy was close to 70% for type I and III IFN and it performed below par when it comes to predicting ISGs activated by type II IFN system. There is scope to improvise the model prediction accuracy and extend its usage to type II IFN systems. If the authors could briefly add few points on how to improve the model accuracy and also highlight the application/impact of this work in their discussion, that would help scientists from other background to resonate with this manuscript.

Our reply: Thanks for the suggestion. We have added some points on how to improve the model accuracy and highlighted the application/impact of this work in the discussion section.

Close
