# Supplementary material for: Defining the characteristics of interferon-alpha–stimulated human genes: insight from expression data and machine learning
Source: Gigascience. 2022 Nov 18;11:giac103. doi: 10.1093/gigascience/giac103 (PMC9673497; doi:10.1093/gigascience/giac103)
Supplement: giac103_Response_to_Reviewer_Comments_Revision_1 [file giac103_response_to_reviewer_comments_revision_1.pdf]

Author's Response To Reviewer Comments

Close

We believe these revisions will address all the concerns raised by you and the reviewers. We apologies for the misunderstanding with respect to the ROC curve and AUC values. We have added these in the legend for each dataset. We have also added ISGPRE to biotools and registered ISGPRE with scicrunch.com and received the RRID:SCR_022730, however it does not yet appear when browsing the scicrunch dashboard. We have also made a few additional clarifications to the text, in particular, after much discussion, we decided to change the ‘noisy’ feature terminology to poorly performing feature (this was originally called disruptive feature).

Close
