# Supplementary material for: Defining the characteristics of interferon-alpha–stimulated human genes: insight from expression data and machine learning
Source: Gigascience. 2022 Nov 18;11:giac103. doi: 10.1093/gigascience/giac103 (PMC9673497; doi:10.1093/gigascience/giac103)
Supplement: giac103_Reviewer_1_Report_Original_Submission — Milton Pividori, Ph.D. -- 3/29/2022 Reviewed [file giac103_reviewer_1_report_original_submission.pdf]

Reviewer Report

Title: Defining the characteristics of interferon-alpha-stimulated human genes: insight from expression data and machine-learning

Version: Original Submission **Date:** 3/29/2022

Reviewer name: Milton Pividori, Ph.D.

Reviewer Comments to Author:

In this manuscript, the authors analyzed different characteristics that are potentially related to the expression of human genes under IFN-a stimulation. A classification model is built to predict ISG (genes that are upregulated following IFN-a stimulation) from the human fibroblast cell. The model also performs feature selection, and the authors used different test sets (on different types of IFN) to validate their model. The authors provide a web server that implemented this machine learning model. I liked the introduction, the background and motivation were clear. However, the Results section was a bit hard to follow, in particular the implementation of the machine learning models, with different classifiers applied inconsistently across distinct features sets. At the beginning of this section, the authors perform extensive _manual_ feature analyses across different feature types (related to alternative splicing, duplication, and mutation) to build a refined dataset. These analyses basically correlate each individual feature with the expression of genes in the presence of IFN-a. I have several concerns here, related mainly to the correlation _between_ features, that I describe below.
General comments:
* Regarding reproducibility, the authors provide a Github repository with source code, the model trained and data. From the documentation and notes in the manuscript (lines 1015-1023), looks like this can only be run on mac OS, which makes it very hard for me to test (I'm a Linux user). I recommend the authors to read and follow the article "Reproducibility standards for machine learning in the life sciences" (https://doi.org/10.1038/s41592-021-01256-7). Having, for instance, a Docker image to download and run your analyses would be fantastic.
* The authors perform a comprehensive analysis of features that differentiate different gene classes. I wonder why didn't they use first a machine learning model to automatically find these important features, and then try to analyze which features were selected (instead of the other way around as done in the study). I think there is perhaps too much manual feature engineering in the previous steps of training an ML model.
* Related to the previous point, in my comments below one of my concerns is about feature correlation. The authors compare individual features regarding their ability to separate different gene classes (ISG vs background vs non-ISG). But one can imagine that some features are highly correlated. Some features might not be useful to separate gene classes from a single-feature analysis (as the authors do at the beginning), but they could be useful in combination with other features. Unless I'm missing an important point, I would leave the machine learning model to learn this and then analyze each feature individually after the model identifies them.
* Authors are concerned that including too many features in the support vector machine (SVM) model

would complicate the prediction task. To remedy this, they manually select the features according to, in my opinion, a more subjective criterion. Why didn't the authors use a feature selection algorithm here? I know that they propose a model including feature selection, but I guess I don't understand well all the previous manual feature analyses. Using a known feature selection method here would provide a more data-driven approach to improve classification, in addition to their manual expert curation (which is also valid).
* They run several classification models, but not consistently across the same set of features. For example, only SVM is run across genetic, parametric, all features, etc, but not the other models. Why is that?
* The manuscript would really benefit from a figure with the main steps of the analyses performed, models tested, datasets employed, etc. It's hard to get the big picture as it is now.
Results/Evolutionary characteristics of ISGs:
Paragraph between lines 131-148:
* I think the window size used (mentioned in the text) should be added to the Figure 2 caption
* What's the vertical dashed line? In the text, you say that those at the left of this line are IRGs, but I don't understand the meaning of that vertical line (-0.9 log fold change). This explanation, which I didn't see, should be added to the figure caption also.
* From the text, I understand that in the subfigures in Figure 2 you have IRGs, non-ISGs and ISGs. Would it be possible, or meaningful for the reader, to add an extra vertical line to separate them?
Results/Differences in the coding region of the canonical transcripts:
Paragraph between lines 193-208:
* If GC-content is underrepresented in ISGs more than non-ISGs, the ApT and TpA should be expected to be more enriched in ISGs, right? Sounds like a redundant analysis. I would expect these two sequence-derived features to be correlated. If this is the case, maybe it would be better to highlight other features instead of a correlated/expected one?
* Figure 4: here the authors divided the parametric set of features into four categories and compared their representations among ISGs, non-ISGs and background genes. The figure shows p-values of the tests on the y-axis, and the four categories of features on the x-axis. I think it's important to run a negative control: could you please run these tests again, say, 100 times, with gene IDs/names shuffled, and check whether some of these results also appear in these null simulations? Maybe you can keep the same figure, but remove those also found in the null simulations.
Paragraph between lines 209-227:
* Is it possible that the comparison of codons frequencies (third category of features) is correlated with previous findings (like GC content or ApT/TpA enrichment)? If so, would it be possible that maybe the analysis is also expected or redundant? For example, in ISGs there is an underrepresentation of GC-content, and you also found that ISGs there is an underrepresentation of "CAG" codons. I might be missing something, but aren't these expected to be correlated?
Results / Differences in the protein sequence:
Paragraph between lines 302-323:
* Figure 6: I would suggest adding the same negative control suggested before.
Results / Differences in network profiles
* I think it's important to define what are all those eight features in the network analyses (closeness,

betweenness, etc), otherwise it's hard to follow what comes next.
Results / Features highly associated with the level of IFN stimulations
* Figures 9 and 10: it would be good to add the sign of the correlation in the figure, in addition to mentioning it in the caption (as it is now).
Results / Difference in feature representation of interferon-repressed genes and genes with low levels of expression
* Given the unique patterns or differences between non-ISG class and IRG class, wouldn't it be better to perform different analyses excluding IRG genes? The authors also acknowledge these risks in lines 539-541.
Results / Implementation with machine learning framework
* It was hard for me to understand the workflow in this section: you used different machine learning models applied to distinct features sets, for example. Why don't you apply the same set of models to the same set of features? I think this section needs an initial paragraph with a global description of what you are trying to do.
* For example, I don't think I understand very well the concept of "disruptive feature". What does it mean?
* Table 3: I don't understand the threshold selection here. I guess you refer to classification or decision threshold from a model that outputs a probability of a gene to be ISG or non-ISG. First, I think there should be a line separating each performance measure to clearly show those that are "Threshold-dependent" and "Threshold independent"
* I also understand that, during cross-validation, you selected for each model/feature set combination, the threshold that maximized the MCC (this is explained in Table 3 as a footnote, but it should be more explicitly mentioned in the text).
* Table 3: What is the "Optimum" set of features? Why is this "Optimium set" only used with SVM?
* How does the "AUC-driven subtractive iteration algorithm (ASI)" compare with other feature selection algorithms.
* Table 5: you mention this in the text, but it would be good to have an extra column indicating which datasets were used for training and which are for testing.
* Figure 13: it would be good to have the AUROC in the figure, not only the curves.
Web-server:
* I think, in general, that the web application needs to be more intuitive and have more documentation. For example, the main interface says "Predict your human genes of interest", what does that mean? What does it predict?

Methods

Are the methods appropriate to the aims of the study, are they well described, and are necessary controls included? Choose an item.

Conclusions

Are the conclusions adequately supported by the data shown? Choose an item.

Reporting Standards

Does the manuscript adhere to the journal's guidelines on minimum standards of reporting? Choose an item.

Choose an item.

Statistics

Are you able to assess all statistics in the manuscript, including the appropriateness of statistical tests used? Choose an item.

Quality of Written English

Please indicate the quality of language in the manuscript: Choose an item.

Declaration of Competing Interests

Please complete a declaration of competing interests, considering the following questions:

- Have you in the past five years received reimbursements, fees, funding, or salary from an organisation that may in any way gain or lose financially from the publication of this manuscript, either now or in the future?
- Do you hold any stocks or shares in an organisation that may in any way gain or lose financially from the publication of this manuscript, either now or in the future?
- Do you hold or are you currently applying for any patents relating to the content of the manuscript?
- Have you received reimbursements, fees, funding, or salary from an organization that holds or has applied for patents relating to the content of the manuscript?
- Do you have any other financial competing interests?
- Do you have any non-financial competing interests in relation to this paper?

If you can answer no to all of the above, write 'I declare that I have no competing interests' below. If your reply is yes to any, please give details below.

I declare that I have no competing interests

I agree to the open peer review policy of the journal. I understand that my name will be included on my report to the authors and, if the manuscript is accepted for publication, my named report including any attachments I upload will be posted on the website along with the authors' responses. I agree for my report to be made available under an Open Access Creative Commons CC-BY license (http://creativecommons.org/licenses/by/4.0/). I understand that any comments which I do not wish to be included in my named report can be included as confidential comments to the editors, which will not be published.

Choose an item.

To further support our reviewers, we have joined with Publons, where you can gain additional credit to further highlight your hard work (see: https://publons.com/journal/530/gigascience). On publication of this paper, your review will be automatically added to Publons, you can then choose whether or not to claim your Publons credit. I understand this statement.

Yes Choose an item.
