# Supplementary material for: Defining the characteristics of interferon-alpha–stimulated human genes: insight from expression data and machine learning
Source: Gigascience. 2022 Nov 18;11:giac103. doi: 10.1093/gigascience/giac103 (PMC9673497; doi:10.1093/gigascience/giac103)
Supplement: giac103_Reviewer_2_Report_Original_Submission — Muthukumaran Venkatachalapathy -- 6/1/2022 Reviewed [file giac103_reviewer_2_report_original_submission.pdf]

Reviewer Report

Title: Defining the characteristics of interferon-alpha-stimulated human genes: insight from expression data and machine-learning

Version: Original Submission **Date:** 6/1/2022

Reviewer name: Muthukumaran Venkatachalapathy

Reviewer Comments to Author:

First of all, this manuscript is well-written after a thorough research investigation. I enjoyed reading about interferons, interferon stimulating genes (ISGs), mechanisms and signalling pathways. In the introduction, the authors have highlighted the different methods (including other bioinformatics databases) available to identify ISGs and their potential pitfalls. This unmet need is addressed using in silico approaches which were used to classify interferon stimulating genes from non-stimulating ones in human fibroblast cells. Here, the authors have applied a combination of expression data and sequential/compositional features and designed a machine learning model for the prediction of ISGs from non-ISGs.

Apart from features like duplication, alternative splicing, mutation and presence of multiple ORFs, the authors extracted various sequential features and found them to be correlated well with ISG prediction. For example, ISGs are prone to GC depletion and a significant difference in the codon usage among ISGs was found. In that context, the authors claim that ISGs are evolutionarily less conserved, codon usage features, genetic composition features, proteomic composition features and sequence patterns (especially like SLNPs and SLAAPs) are optimal parameters that can cumulatively help in differentiating ISGs from non-ISGs.

When it comes to building a machine learning model, the authors faced challenges due to similarities between ISGs and IRGs. They have experimented using different algorithms for model building ranging from the decision tree, and random forest and found decent results with support vector machine.

Limitation: Model Prediction accuracy was close to 70% for type I and III IFN and it performed below par when it comes to predicting ISGs activated by type II IFN system. There is scope to improvise the model prediction accuracy and extend its usage to type II IFN systems. If the authors could briefly add few points on how to improve the model accuracy and also highlight the application/impact of this work in their discussion, that would help scientists from other background to resonate with this manuscript.

Relevance: I believe there are inherent attributes (genetic, compositional, expression) with ISGs which may facilitate or even elevate their expression after IFN stimulation. On the other end, I think these properties may also be leveraged by the viruses to escape or evolve from IFN mediated antiviral response.

This study is relevant during the on-going pandemic, this bioinformatics tool can help design better drug target and may indirectly aid in developing novel antiviral compounds.

I recommend this work for publication without any changes.

Methods

Are the methods appropriate to the aims of the study, are they well described, and are necessary controls included? Choose an item.

Conclusions

Are the conclusions adequately supported by the data shown? Choose an item.

Reporting Standards

Does the manuscript adhere to the journal's guidelines on minimum standards of reporting? Choose an item.

Choose an item.

Statistics

Are you able to assess all statistics in the manuscript, including the appropriateness of statistical tests used? Choose an item.

Quality of Written English

Please indicate the quality of language in the manuscript: Choose an item.

Declaration of Competing Interests

Please complete a declaration of competing interests, considering the following questions:

- Have you in the past five years received reimbursements, fees, funding, or salary from an organisation that may in any way gain or lose financially from the publication of this manuscript, either now or in the future?
- Do you hold any stocks or shares in an organisation that may in any way gain or lose financially from the publication of this manuscript, either now or in the future?
- Do you hold or are you currently applying for any patents relating to the content of the manuscript?
- Have you received reimbursements, fees, funding, or salary from an organization that holds or has applied for patents relating to the content of the manuscript?
- Do you have any other financial competing interests?
- Do you have any non-financial competing interests in relation to this paper?

If you can answer no to all of the above, write 'I declare that I have no competing interests' below. If your reply is yes to any, please give details below.

I have no conflict of interest.

I agree to the open peer review policy of the journal. I understand that my name will be included on my report to the authors and, if the manuscript is accepted for publication, my named report including any attachments I upload will be posted on the website along with the authors' responses. I agree for my report to be made available under an Open Access Creative Commons CC-BY license (http://creativecommons.org/licenses/by/4.0/). I understand that any comments which I do not wish to be included in my named report can be included as confidential comments to the editors, which will not be published.

Choose an item.

To further support our reviewers, we have joined with Publons, where you can gain additional credit to further highlight your hard work (see: https://publons.com/journal/530/gigascience). On publication of this paper, your review will be automatically added to Publons, you can then choose whether or not to claim your Publons credit. I understand this statement.

Yes Choose an item.
